# Identification of Contractile Vacuole Proteins in *Trypanosoma cruzi*

**DOI:** 10.1371/journal.pone.0018013

**Published:** 2011-03-18

**Authors:** Paul N. Ulrich, Veronica Jimenez, Miyoung Park, Vicente P. Martins, James Atwood, Kristen Moles, Dalis Collins, Peter Rohloff, Rick Tarleton, Silvia N. J. Moreno, Ron Orlando, Roberto Docampo

**Affiliations:** 1 Center for Tropical and Emerging Global Diseases and Department of Cellular Biology, University of Georgia, Athens, Georgia, United States of America; 2 Complex Carbohydrate Research Center, University of Georgia, Athens, Georgia, United States of America; Institut national de la santé et de la recherche médicale - Institut Cochin, France

## Abstract

Contractile vacuole complexes are critical components of cell volume regulation and have been shown to have other functional roles in several free-living protists. However, very little is known about the functions of the contractile vacuole complex of the parasite *Trypanosoma cruzi*, the etiologic agent of Chagas disease, other than a role in osmoregulation. Identification of the protein composition of these organelles is important for understanding their physiological roles. We applied a combined proteomic and bioinfomatic approach to identify proteins localized to the contractile vacuole. Proteomic analysis of a *T. cruzi* fraction enriched for contractile vacuoles and analyzed by one-dimensional gel electrophoresis and LC-MS/MS resulted in the addition of 109 newly detected proteins to the group of expressed proteins of epimastigotes. We also identified different peptides that map to at least 39 members of the dispersed gene family 1 (DGF-1) providing evidence that many members of this family are simultaneously expressed in epimastigotes. Of the proteins present in the fraction we selected several homologues with known localizations in contractile vacuoles of other organisms and others that we expected to be present in these vacuoles on the basis of their potential roles. We determined the localization of each by expression as GFP-fusion proteins or with specific antibodies. Six of these putative proteins (Rab11, Rab32, AP180, ATPase subunit B, VAMP1, and phosphate transporter) predominantly localized to the vacuole bladder. TcSNARE2.1, TcSNARE2.2, and calmodulin localized to the spongiome. Calmodulin was also cytosolic. Our results demonstrate the utility of combining subcellular fractionation, proteomic analysis, and bioinformatic approaches for localization of organellar proteins that are difficult to detect with whole cell methodologies. The CV localization of the proteins investigated revealed potential novel roles of these organelles in phosphate metabolism and provided information on the potential participation of adaptor protein complexes in their biogenesis.

## Introduction

*Trypanosoma cruzi* is the etiologic agent of Chagas disease, the leading cause of heart disease in endemic areas of Latin America [1]. Living in a wide range of environments, *T. cruzi* developed ways of coping with sudden or prolonged changes in its surroundings. *T. cruzi* experiences osmotic challenges as it passes between the blood of mammalian hosts (300 mOsm) and the rectum of insect hosts (>750 mOsm) [2]. In addition, bloodstream trypomastigotes must be able to resist up to 1,400 mOsm when passing through the renal medulla and return to the isosmotic environment at the general circulation [3]. Thus, similar to erythrocytes, these parasites must have fast and efficient mechanisms to face such extreme challenges.

Previous studies of osmotic stress in *T. cruzi* demonstrated that a contractile vacuole (CV) complex contributes to regulatory volume decrease under hyposmotic stress [4]–[6]. The roles of the contractile vacuoles in protists, though, extend beyond regulation of cell volume to regulation of Ca^2+^ homeostasis [7]–[9] and transport of proteins to the plasma membrane [10]. Recently, Hasne et al. [11] demonstrated that the contractile vacuole of *T. cruzi* houses a polyamine transporter that can be transferred to the plasma membrane when the incubation media is deficient in polyamines.

Knowledge of the protein composition of the CV will facilitate understanding of the physiological roles of these organelles in *T. cruzi*. Only a few proteins have been localized to the CV of *T. cruzi* so far. Among these are vacuolar proton pyrophosphatase (TcPPase or TcVP1) [5], aquaporin 1 (TcAQP1) [5], calmodulin [5], cyclicAMP phosphodiesterease C (TcPDEC) [12], alkaline phosphatase [4], and a polyamine transporter (TcPOT1) [11]. The contribution of each to *T. cruzi* physiology is limited largely to roles in cell volume regulation. Hyposmotic stress increases cAMP concentration in *T. cruzi*, and stimulates the translocation of TcAQP1 to the CV from acidocalcisomes [4], acidic organelles containing high amounts of polyphosphate and cations [13]. A current model suggests that hydrolysis of polyphosphate osmotically drives water from the cytosol into the CV and that termination of this process occurs after hydrolysis of cAMP by the CV-localized TcPDEC [6]. However, validation of this model awaits further elucidation of the regulatory and effector proteins that populate the CV.

In this study, we used a combined proteomic and bioinformatic strategy to identify proteins of the CV. We validated their CV localization by their expression as GFP-fusion proteins and by immunofluorescence with specific antibodies. The results support the role of these organelles in osmoregulation.

## Results

### Protein identification

We identified 220 (1% false discovery rate, total protein group probability >0.95) proteins from fractions enriched in CVs from *T. cruzi* epimastigotes (see Tables S1 and S2). Seventy-four are annotated as “hypothetical” in the *T. cruzi* genome. Seventy five (38 “hypothetical”) were not represented in proteomic data available on TriTrypdb.org (downloaded 4/10/2009) or the ribosomal proteome [14]. One hundred nine were not previously identified in epimastigote data from these sources.

Of the newly identified proteins the most interesting are several members of the dispersed gene family 1 (DGF-1). The *DGF-1* is a large gene family predicted in the *T. cruzi* genome with over 500 members [15]. We identified peptides that map to at least 39 members of this family (see Table S3) providing evidence, for the first time, that many of these proteins are simultaneously expressed in epimastigotes. A second interesting group is that of the calpain-like cysteine peptidase with peptides that unambiguously map to 4 different pseudogenes (see Table S2). Calpain-like proteins are related to Ca^2+^ dependent cytosolic cysteine peptidases (calpains) but lack the Ca^2+^-binding EF-hand domain motif of the domain IV of conventional calpains [16]. Another important finding was the identification of 2 amastins in the subcellular proteome of epimastigotes. Amastins are transmembrane glycoproteins encoded by a large gene family found predominantly on the cell surface of *T. cruzi* and *Leishmania* spp. amastigotes [17].

**Figure 1:**
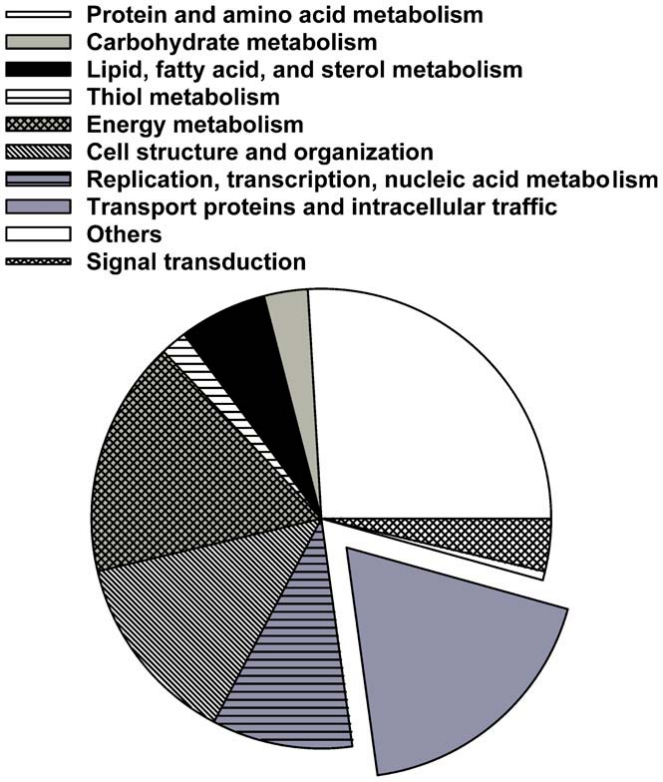
Annotated proteins in the contractile vacuole proteome belong to a variety of metabolic groups.

Approximately 29% (70) of the 220 proteins we identified in our subcellular fraction have predicted transmembrane domains (see Table S4), consistent with estimates of representation in other organisms [18]. Fifty-five proteins (22.9%) possessed putative transmembrane domains but no signal peptide. Annotated proteins in the subcellular proteome analyzed span a broad range of metabolic groups (Fig. 1, and Table S5). Transport-related and intracellular proteins accounted for ∼21%. Among these were small G proteins (Rabs), transporters, and channels. *T. cruzi* vacuolar-H^+^-pyrophosphatase, an acidocalcisomal marker also found in the CV [5], was clearly identified in our dataset (total protein probability  =  1). Other well-represented metabolic groups in our dataset were energy metabolism (19%), protein and amino acid metabolism (29%), and cell structure and organization (15%).

Subcellular localizations of each protein were predicted (Table S6). Targeting predictions based on consensus of at least two algorithms indicated that ∼24% of the proteins are mitochondrial, ∼19% are cytosolic, and ∼5.4% are nuclear. Plasma membrane, Golgi, and secretory localizations represented a small minority. Sixty proteins (29.5%) had one or more predicted transmembrane domains, and 26 (11.8%) proteins had predicted signal peptides.

Of the proteins identified by proteomic analysis of the subcellular fraction we selected a number of proteins with homologues that localize to the CV in other organisms, or properties that could justify CV localization for further validation (Table 1). *T. cruzi* Rab11, while unidentified in our dataset, is included in Table 1 because it localizes in CV bladders of *Dictyostelium discoideum* [19]. The Rab11 gene we cloned and sequenced is identical to a gene (Tc00.1047053511407.60) annotated in the TriTrypDB database but differs from a gene previously named Rab11 [20]. The Rab11 sequence previously described contains insertions of two bases that shift the reading frame. VAMP1 is included because of its similarity (2.8e^−12^) with PtSyb2-2, a *Paramecium tetraurelia* synaptobrevin that localizes to the contractile vacuole [21]. Calmodulin is included in Table 1 because it was shown to be localized in the CV of *T. cruzi* by immunofluorescence analysis using human antibodies [5] and is present in the CV of *D. discoideum* [22] and *Paramecium multimicronucleatum* [23]. A homologue to a vacuolar phosphate transporter from yeast (Pho91) [24] annotated as a sodium/sulphate symporter is also included because orthophosphate (Pi) was shown to be abundant in the CV of *T. cruzi* [4].

**Table 1:** Proteins identified as potentially present in the contractile vacuole complex, showing localizations confirmed in this study or in other organisms.

Protein name	TriTrypDB Gene ID(GenBank ID)	Peptides	Mascot score	CVC localization(this work)	Protists with homologous CV protein	References
V-H^+^-ATPase subunit B (vacuolar synthase subunit B)	Tc00.1047053506025.50(EAN86474.1)	AIQSGYSVKPHLEYTTIR	25	bladder	*D. discoideum* *A. proteus* *C. reinhardtii*	[33]–[35]
V-H^+^-ATPase subunit a	Tc00.1047053509601.70(EAN92926.1)	ERVPILER	18		*D. discoideum* *A. proteus* *C. reinhardtii*	[33]–[35]
V-H^+^-ATPase subunit D (vacuolar ATP synthase subunit D)	Tc00.1047053509017.30(EAN86631.1)	EALAR	17		*D. discoideum* *A. proteus* *C. reinhardtii*	[33]–[35]
V-H^+^-ATPase subunit G (H^+^-ATPase G subunit)	Tc00.1047053510993.10(EAN81810.1)	AQQLSGADENLELAR	23		*D. discoideum* *A. proteus* *C. reinhardtii*	[33]–[35]
SNARE 2.1 (hypothetical protein)	Tc00.1047053507625.183(EAN94402.1)	TAPVR	16	spongiome	*P. tetraurelia*	[21]
SNARE 2.2 (hypothetical protein)	Tc00.1047053506715.50(EAN84023.1)	EVEMFNDK; KILANIKRPLVER	37	spongiome	*P. tetraurelia*,	[21]
Rab32 (small GTP-binding protein)	Tc00.1047053506289.80(EAN96691.1)	NTSGK	17	bladder		N/A
Rab11*	Tc00.1047053511407.60(EAN88612.1)	N/A	N/A	bladder	*D. discoideum*	[19]
Calmodulin*	Tc00.1047053507483.39(EAN86242.1)	N/A	N/A	spongiome	*D. discoideum*,*P. multimicronleatum*	[22], [23]
Phosphate transporter (Pho1)(sodium/sulphatesymporter)*	Tc00.1047053508831.60(EAN97069.1)	N/A	N/A	bladder		
AP180 (clathrin coat assembly protein)	Tc00.1047053503449.30(EAN83025.1)	LSSIPR	18	bladder	*D. discoideum*	[28], [38]
Golvesin-1 (methyltransferase)	Tc00.1047053509805.40(EAN98089.1)	VPIAK	16		*D. discoideum*	[25]
Golvesin-2 (hypothetical protein)	Tc00.1047053503455.30(EAN84949.1)	TCESIGVVSDPVR	15		*D. discoideum*	[25]
myosin heavy chain (myosin V-1)	Tc00.1047053511527.70(EAN87803.1)	MGFPR	20		*D. discoideum* *A. castellani*	[26], [27]
myosin IB heavy chain (myosin V-2)	Tc00.1047053507739.110(EAN89650.1)	ACVDFK	17		*D. discoideum* *A. castellani*	[26], [27]
clathrin heavy chain	Tc00.1047053506167.50(EAN87927.1)	TWTAVNIACIEANEIK	17		*D. discoideum*	[28], [29]
V-H^+^-PPase	Tc00.1047053510773.20(EAN91609.1)	AADVGADLVGK; EITDALDAAGNTTAAIGK; NVYVISR	205		*D. discoideum*	[5]
neurobeachin/beige protein	Tc00.1047053511159.7(EAN84625.1)	EVVENK	16		*D. discoideum*	[30]
IP_3_/ryanodine receptor (hypothetical protein)	Tc00.1047053509461.90(EAN89926.1)	SSRQEIVQDVMFLR;LLGSIDLFMR	15		*P. tetraurelia*	[31]
Calcium channel protein	Tc00.1047053504105.130(EAN97848.1)	ALTGGRTPQELEDKNR; DDNAMYEEALLFDR; LPGLYQPAIDEK	18			
Disgorgin (rab-like GTPase activating protein, RabGAP)	Tc00.1047053508723.80(EAN91303.1)	EKHDLPAK	20		*D. discoideum*	[32]
VAMP (vesicle-associated membrane protein)	Tc00.1047053511627.60(EAN87604.1)	N/A	N/A	bladder	*D. discoideum*	

References of contractile vacuole homologue proteins present in other protists are indicated. The proteins denoted with an asterisk were not present in the MS data. Protein names from the annotated genome, if different from usages in the text, are provided in parenthesis.

Table 1 also shows other proteins that were present in our proteomic analysis and that have homologues in other organisms that localize to the CV, such as a golvesins [25], myosins [26], [27], clathrin heavy chain [28], [29], neurobeachin [30], IP_3_/ryanodine receptor [31], and disgorgin [32]. A putative calcium channel was also detected although there are no reports of this type of channels in the CV.

### V-H^+^-ATPase subunit B localizes mainly to the bladder of the CV

**Figure 2:**
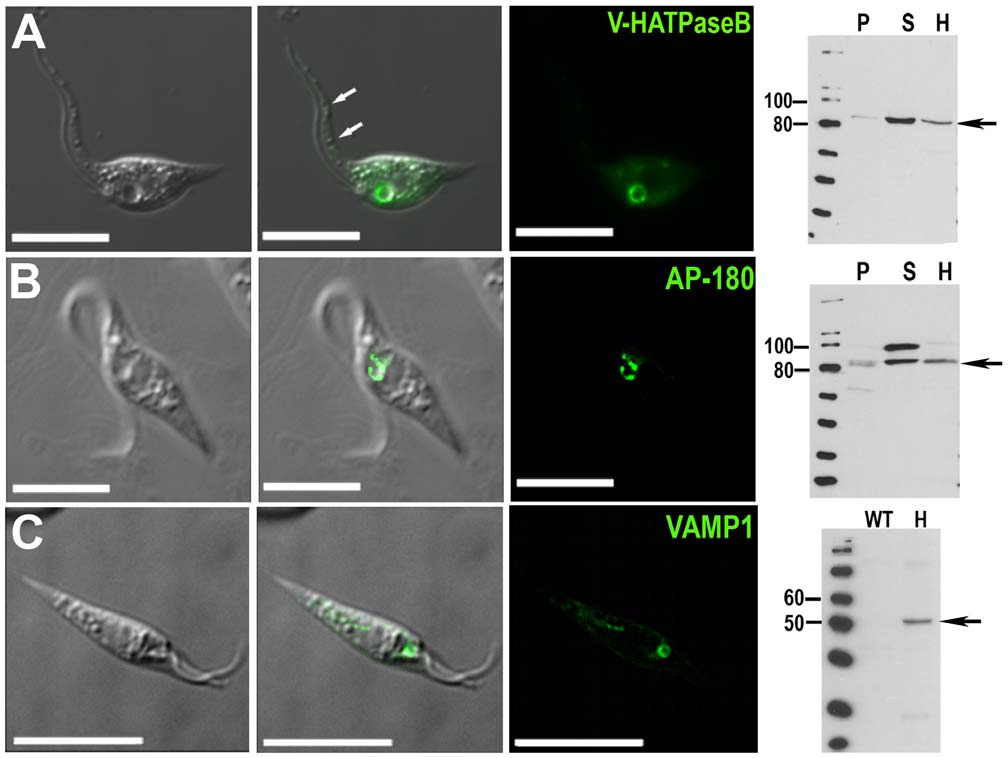
Fluorescence microscopy and western blot analysis of V-H^+^-ATPase subunit B-, AP180-, and VAMP1-GFP fusion proteins in live *T. cruzi* epimastigotes. V-H^+^-ATPase subunit B (**A**), AP180 (**B**), and VAMP1 (**C**) localize to the bladder under hyposmotic conditions. Brightness and contrast of panels was adjusted, and fluorescence images in **C** were deconvolved. Scale bars: 10 µm. Confirmation of tagging by western blot analyses with polyclonal anti-GFP (dilution 1∶5,000-1∶10,000, Invitrogen) in epimastigotes. HRP-conjugated goat anti-rabbit was used as a secondary antibody. Magic Mark XP (Invitrogen) was used as a molecular weight marker. Arrows indicate bands of interest. **A**, V-H^+^-ATPase subunit B, expected size of fusion protein  =  82 kDa. **B**, AP-180, expected size of fusion protein =  81 kDa. A 100 kDa cross-reacting band is only detected in the supernatant. **C**, VAMP1 expected size =  52 kDa. **P**, membrane pellet, **S**, soluble fraction, **H**, homogenate of whole parasites, **WT**, wild-type epimastigotes (negative control).

Vacuolar-H^+^-ATPase is a multisubunit complex that is a marker for the CV in *D. discoideum* [33], *Amoeba proteus* [34], and *Chlamydomonas reinhardtii* [35]. Several subunits were detected in our proteomic analysis (a, B, D, and G, Table 1). In early work we detected co-localization of this V-H^+^-ATPase with a plasma membrane-type Ca^2+^-ATPase (PMCA) to the acidocalcisomes of *T. cruzi* [36]. To investigate the localization of this proton pump, we generated a V-H^+^-ATPase subunit B-GFP fusion expression construct and transfected epimastigotes with it. Subunit B of *T. cruzi* V-H^+^-ATPase is 71% identical to its homologous in *D. discoideum*. After several weeks of selection, expression of the fusion protein in the transfectants was analyzed by direct fluorescence analysis. Although detectable under isosmotic conditions (when the CV was collapsed in most cells), V-H^+^-ATPase subunit B strongly delineated the outer margin of the enlarged CV bladder under hyposmotic conditions (150 mOsm) (Fig. 2A). Additional labeling was detected in smaller vacuoles, which are visible in the differential interference contrast (DIC) images (Fig. 2A, arrows) and could correspond to acidocalcisomes, which are known to increase in volume under hyposmotic stress [4]. We confirmed C-terminal tagging of *T. cruzi* V-H^+^-ATPase subunit B with GFP by western blot analysis (Fig. 2A). Although this subunit was present in the total cell homogenate and in the 100,000 *g* pellet, it was also detected in the 100,000 *g* supernatant. The presence of subunit B in the soluble fraction is due to the well-known dissociation and loss of peripheral subunits of the V-H^+^-ATPase that occurs during cell fractionation of *T. cruzi* [37]. This subunit associated with but did not co-localize with calmodulin (CaM), a protein that localizes to a compartment proximal to the bladder [4],[22] (data not shown).

### AP180 localizes to the bladder

AP180 is a protein that promotes assembly of clathrin triskelia and is localized in the plasma membrane and contractile vacuole of *D. discoideum* [38]. The *T. cruzi* orthologue (annotated “clathrin coat assembly protein”) contains a conserved ANTH (AP180 N-terminal homology) domain. Within the ANTH domain of *T. cruzi* AP180 is a region similar to the domain consensus sequence [KG]A[TI]xxxxxx[PLV]KxK[HY] [39]. Additionally, *T. cruzi* AP180 contains a clathrin box motif (LVAVE) and a tyrosine-based sorting motif (YAAL, detected with the ELM server [40]), which may mediate clathrin and adaptor protein 2 (AP-2) complex interactions, respectively [41], [42].

**Figure 3:**
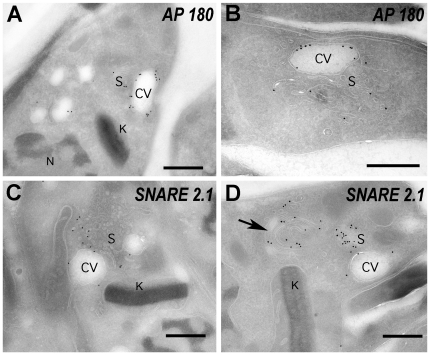
AP180 and SNARE 2.1 immuno-electron microscopy. GFP fusion proteins were detected in epimastigotes with anti-GFP polyclonal antibodies and gold-conjugated anti-rabbit secondary antibody. AP180 localizes mainly in the bladder of the CV (**A** and **B**) while SNARE 2.1 clearly localizes in the vesicular structures of the spongiome (**C** and **D**) although, some labeling can be observed is Golgi-like structures (arrow in **D**). CV: contractile vacuole bladder; S: spongiome; K: kinetoplast; N: nucleus. Bars: 0.5 µm.

AP180 fused to GFP (*N*-terminal tag) was present in the CV bladder (Fig. 2B) and detected by western blot analysis (Fig. 2B). To investigate whether AP180 resides in the bladder, we observed live cells under hyposmotic conditions (Fig. 2B) and localized AP180 with antibodies against GFP and CaM in fixed cells (see Fig. S1). AP180 was also present in a structure adjacent to the bladder stained by antibodies against CaM (see Fig. S1). The vesicular structure of the bladder was not evident because fixation collapses the CV. Immunogold electron microscopy confirmed the predominant localization of AP180 in the bladder of the CV and to some tubules and vesicles that form the spongiome (Fig. 3A,B), a network of collecting ducts connected to the bladder.

### VAMP1 localizes to the bladder

Soluble N-ethylmaleimide-sensitive factor (NSF) adaptor proteins (SNAPs) receptors (SNAREs) are key components of the intracellular vesicle-mediated transports that take place in eukaryotic cells and are distinguished by the presence of a common motif (SNARE motif). These proteins can be classified as Q- and R-SNAREs according to the residue present in the center of the motif [43]. The Q group can be further divided into three subgroups according to their overall homology in the SNARE domain: Qa (or syntaxins), Qb (or SNAP N-terminal) and Qc (or SNAP C-terminal) [44]. The vesicle associated membrane proteins (VAMPs) belong to the R-SNAREs group. *Paramecium tetraurelia* R-SNARE PtSyb2-2 has been shown to localize to the entire contractile vacuole complex [21], and its orthologue in *T. cruzi* (VAMP1) fused to GFP (N-terminal) was detected mainly in the CV bladder of epimastigotes submitted to hyposmotic stress (Fig. 2C). Western blot analysis of homogenates revealed the presence of the fusion protein of the expected apparent molecular mass (52 kDa) (Fig. 2C).

### Rab11 and Rab32 localize to the bladder

**Figure 4:**
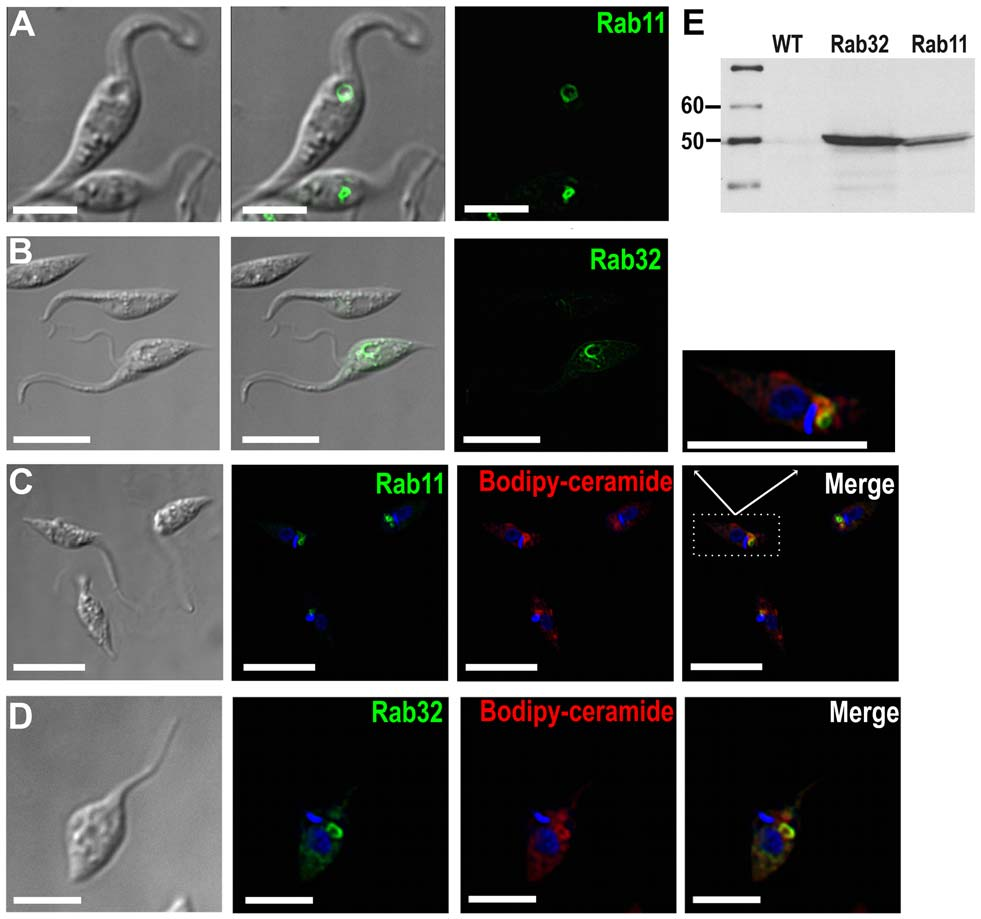
Rab-GFP fusion proteins localize in *T. cruzi* contractile vacuole. Rab11 (green) (**A**) land Rab32 (green) (**B**) localize to the bladder under hyposmotic conditions. Rab11 (**C**) and Rab32 (**D**) partially co-localize with BODIPY-ceramide (red). DNA is stained with DAPI (blue). Brightness and contrast of panels was adjusted, and fluorescence images in **C**-**D** were deconvolved. Inset in C shows one cell (dotted rectangle) at higher magnification. Scale bars: **A**, **B** and **C** =  10 µm; **D** =  5 µm. **E**, confirmation of tagging by western blot analyses with anti GFP shows the expected size for both fusion proteins (50 kDa). Wild-type epimastigotes were used as negative control (WT).

Rab (“Ras-related in brain”) GTPases have emerged as central regulators of vesicle budding, motility, and fusion. They typically localize to the cytosolic face of distinct intracellular membrane [45]. Rab11 has been reported to associate with and regulate the structure and function of the contractile vacuole complex of *D. discoideum* [19]. On the other hand, Rab32 has been reported to function as an A-kinase anchoring protein in mitochondria [46] and melanosomes [47] of mammals. Orthologues to these Rabs are present in *T. cruzi*, although Rab32 orthologues have not been found in other trypanosomatids. GFP-Rab11 and GFP-Rab32 (*N*-terminal fusions) localized to the CV bladder membrane in epimastigotes (Figs. 4A, and 4B). Hyposmotic stress improved direct visualization of GFP-Rab11 fluorescence in the CV bladder (Fig. 4A). In addition to the bladder, GFP-Rab32 commonly appeared in rod-like structures near the CV region. Immunofluorescence microscopy of GFP-Rab11 using antibodies against GFP and CaM confirmed that GFP-Rab11 was associated with a compartment distinguishable from the spongiome of the CV (results not shown). We confirmed tagging of Rab32 and Rab11 by western blot analyses (Fig. 4E). We also investigated the co-localization of these Rabs with BODIPY-ceramide conjugated to BSA, which labels the Golgi stacks in mammalian cells [48] and trypanosomes [49] as well as the contractile vacuole of *P. tetraurelia* [50]. GFP-Rab32 strongly co-localized with BODIPY-ceramide (Fig 4D and Supplementary Movie S2). GFP-Rab11 demonstrated only partial co-localization (Fig. 4C and Supplementary Movie S1).

### SNARE2.1 and SNARE2.2 associate with the spongiome

**Figure 5:**
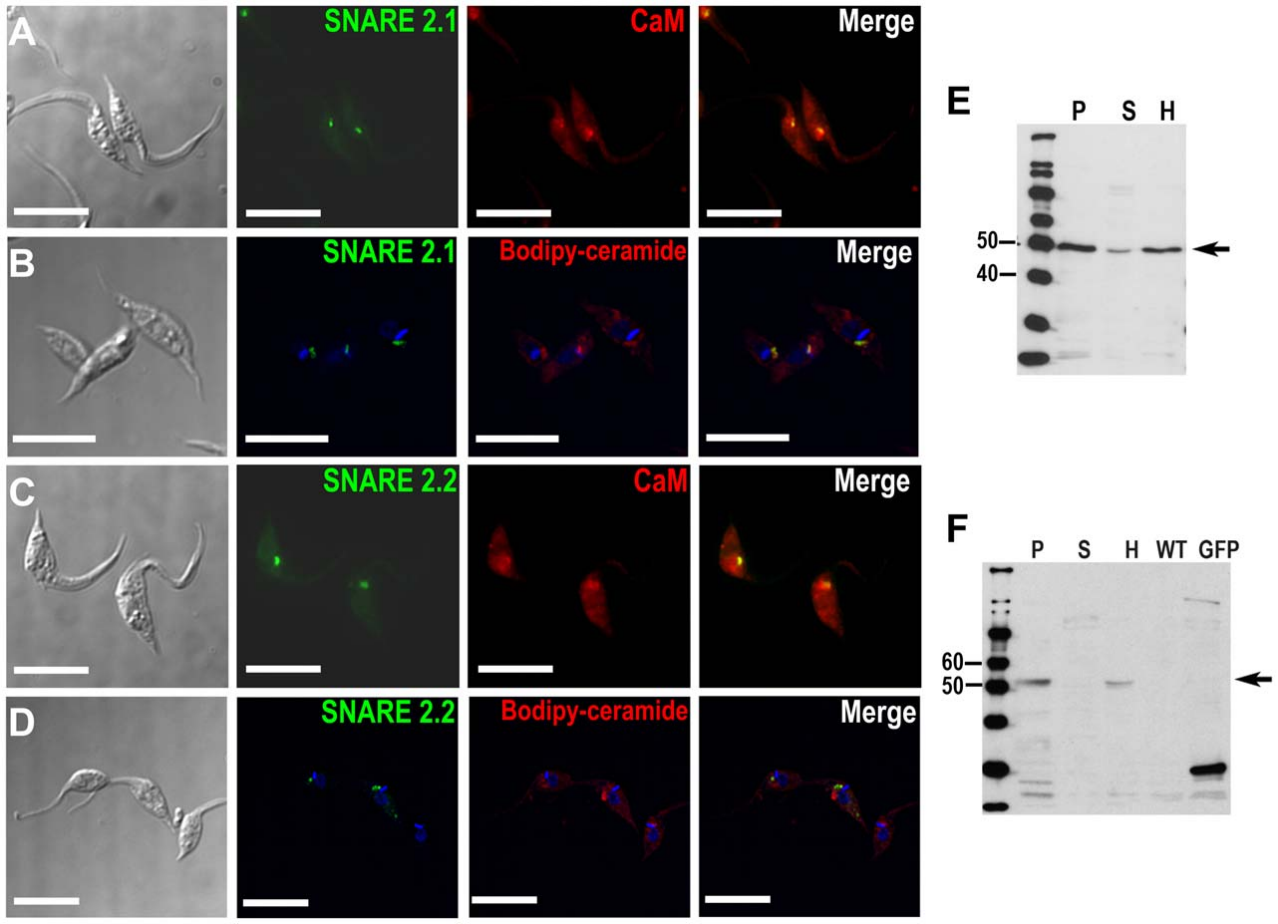
SNARE-GFP fusion proteins localize in the contractile vacuole spongiome. SNARE2.1-GFP co-localizes with calmodulin (CaM) (**A**) and BODIPY-ceramide (**B**). SNARE2.2-GFP co-localizes with CaM (**C**) but localizes to a compartment that does not stain with BODIPY-ceramide (**D**). DNA is stained with DAPI (blue). Brightness and contrast of panels was adjusted, and fluorescence images in **B** and **D** were deconvolved. Scale bars =  10 µm. **E**, **F**, western blot analyses reveal the expected size for GFP tagged SNARE proteins (50 kDa). **P**, membrane pellet; **S**, soluble fraction, **H**, homogenate of whole parasites; **WT**, wild-type epimastigotes (negative control); **GFP**, epimastigotes overexpressing GFP (positive control).

Peptides corresponding to two other R-SNARES (SNARES2.1 and SNARE2.2) were present in our proteomic data of CV-enriched fractions (Table 1). TcSNARE2.1-GFP and TcSNARE2.2-GFP were detected in the CV under iso-osmotic conditions while hyposmotic shock did not alter their localization (data not shown). We co-localized TcSNARE2.1-GFP and TcSNARE2.2-GFP with calmodulin in a region proximal to the bladder of the CV (Figs. 5A, and 5C). TcSNARE2.1-GFP also co-localized with BODIPY-ceramide (Fig. 5B), but TcSNARE2.2-GFP very weakly co-localized with this fluorescent stain (Fig. 5D). Tagging of TcSNARE2.1-GFP and TcSNARE2.2-GFP was confirmed by western blot analyses (Fig. 5E, and 5F). Both TcSNAREs were present mainly in the membrane fraction. The localization of SNARE2.1 to the spongiome of the CV was confirmed by immunogold electron microscopy (Fig. 3C, D).

### Calmodulin (CaM) associates with the spongiome and cytosol

**Figure 6:**
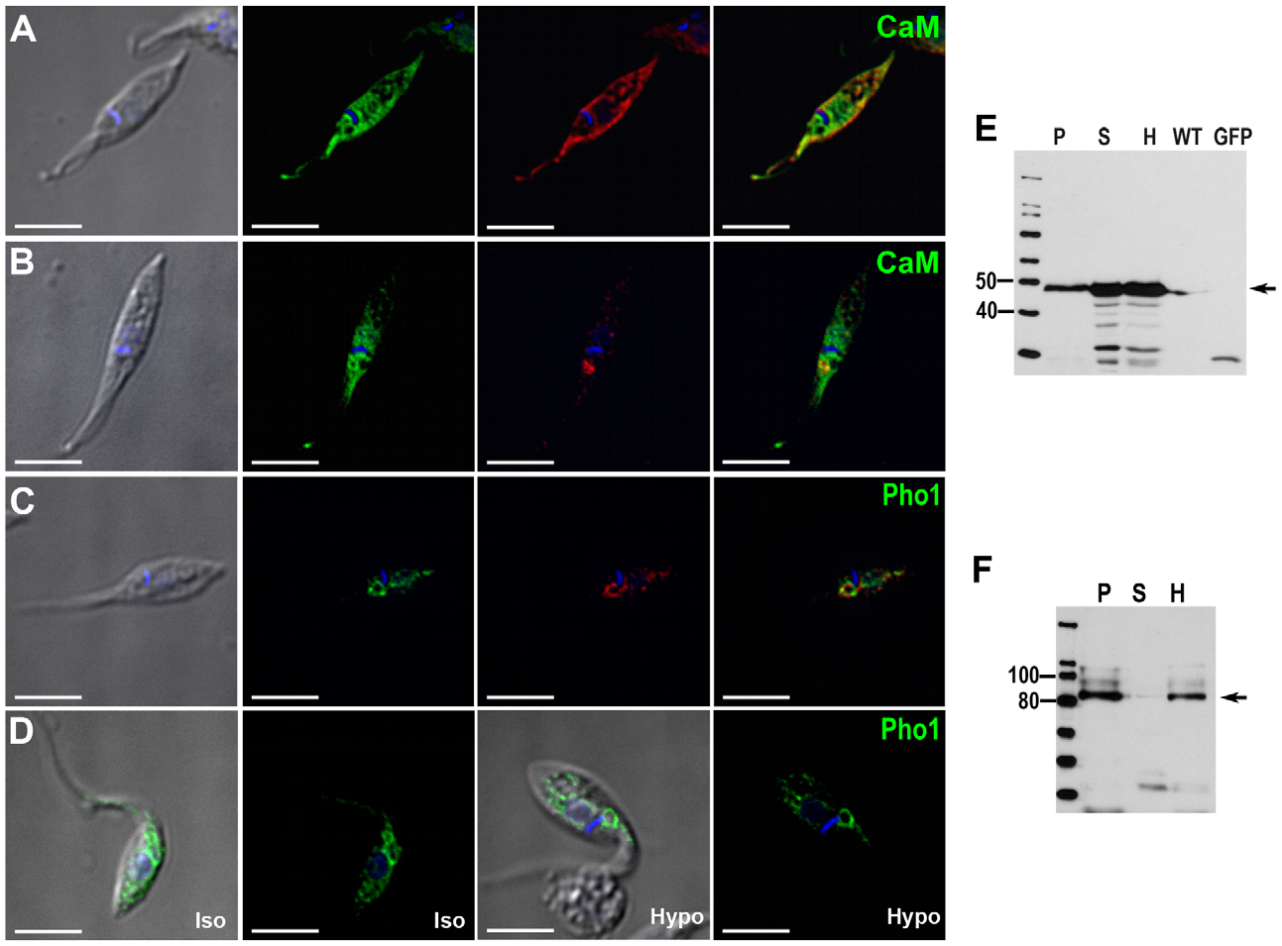
CaM- and TcPho1-GFP fusion proteins localization. CaM-GFP overexpressing parasites (**A**) showed a localized signal to the contractile vacuole (green) but also cytosolic distribution. This localization was confirmed with anti-GFP (**A**, in red). A monoclonal antibody against human CaM was used to perform IFA (**B**). Green corresponds to the CaM-GFP overexpressed protein and in red the specific localization for the anti-human CaM can be observed in the spongiome of the CV. TcPho1 is localized at the bladder of the CV (**C**) both by direct GFP signal (green) and labeled by anti-GFP (red). Under hyposmotic conditions (**D**, hypo), TcPho1 bladder localization becomes more evident. The expected molecular weight for fusion proteins is 44 kDa for CaM (**E**) and 107 kDa for TcPho1 (**F**). **P**, membrane pellet; **S**, soluble fraction; **H**, homogenate whole parasites; **WT**, wild-type epimastigotes (negative control); **GFP**, epimastigotes overexpressing GFP (positive control).

As described above antibodies against human CaM (>92% identical to TcCaM) localize to the same compartment labeled with SNARE2.1-GFP (spongiome, Fig. 3C, D) and to the cytosol (see Fig. S1, Figs. 5A, and 5C). When CaM was expressed as a fusion construct with GFP, the protein also showed cytosolic staining in addition to CV localization (Fig. 6A). Tagging of CaM-GFP was confirmed by western blot analysis (Fig. 6E). In epimastigotes overexpressing CaM-GFP fusion proteins, immunolocalization with anti-human CaM antibody only showed partial co-localization in the CV spongiome (Fig. 6B).

### Phosphate transporter localizes to the bladder

Pho91 is a vacuolar phosphate transporter that regulates phosphate and polyphosphate metabolism in *Saccharomyces cerevisiae* [24]. The *T. cruzi* orthologue, when expressed as a fusion with GFP, strongly localized to the bladder of the CV and also showed some granular localization in the cytosol (Fig. 6C). Under hyposmotic stress, the localization of the phosphate transporter becomes more evidently associated with the CV bladder, which is clearly delineated by the GFP-labeled protein (Fig. 6D). Western blot analysis confirmed the expression of this construct (Fig. 6F). The recognized polypeptide had an apparent molecular mass of 87 kDa. Since *T. cruzi* Pho1 has 12 transmembrane domains, a size discrepancy between the expected (107 kDa) and the observed molecular mass could be attributed to the usual anomalous migration of hydrophobic protein on SDS gels [51] or to partial degradation. In agreement with these results, the protein could not be detected if the samples were boiled before SDS gel electrophoreses.

## Discussion

We report here the proteomic analysis of a subcellular fraction enriched in CV from *T. cruzi*. Previous work [4] indicated that this fraction is enriched in the contractile vacuole markers alkaline phosphatase [52] and bafilomycin A_1_-sensitive vacuolar H^+^-ATPase [33]–[35], basic amino acids, and phosphate. Additionally, this protocol yields fractions well resolved from organelle markers for mitochondria (alanine aminotransferase), glycosomes (hexokinase) and lysosomes (α-mannosidase) [4].

We identified 220 proteins (1% false discovery rate, protein group probability >0.95, (see Table S2) in this dataset. Among the annotated proteins, we identified vacuolar-H^+^-pyrophosphatase (VP1), a proton pump that co-localizes with aquaporin found in the *T. cruzi* CV [5]. VP1 is a major component of acidocalcisomes, acidic organelles that fuse with the CV in response to osmotic challenge [4].

Many proteins we identified likely belong to different cellular compartments, but the relatively high representation of membrane proteins (65 proteins, 29.5%) is notable, given that membrane proteins are challenging for proteomic analysis. In comparison, the recently published plasma membrane proteome of *Trypanosoma brucei* contains a lower proportion of membrane proteins (16.1% of 1536 proteins, [53]). Thus we feel that our fractionation successfully enriched proteins with potential membrane-related functions.

A comparison with previous whole cell protein expression studies carried out in *T. cruzi* revealed that our proteomic analysis resulted in the identification of 75 previously undetected proteins. This confirms the validity of subcellular proteomics as a method of choice for the identification of larger number of proteins than with whole cell proteomics [54]. Our identification of DGF-1 proteins illustrates this clearly. Only one glycopeptide that maps to several members of the DGF-1 was previously identified in a glycoproteomic study of *T. cruzi* trypomastigotes [55]. We recently reported that these proteins localize to a population of organelles which do not co-localize with markers of acidocalcisomes, glycosomes, reservosomes, lipid droplets, or endocytic vesicles in different stages of *T. cruzi* [56]. We also demonstrated that these proteins are released from trypomastigotes during their differentiation into amastigotes. Proteomic analysis of supernatants from these incubations contained peptides mapping to at least 22 DGF-1 members [56]. Here we report the detection of peptides that map to at least 39 DGF-1 members in *T. cruzi*, providing definitive evidence of simultaneous expression of many of these proteins in epimastigotes.

The detection of peptides mapping to several calpain-like cysteine peptidases pseudogenes suggests that these were inaccurately annotated in the genome. Finally, the presence of peptides that map to amastin proteins indicates that these proteins are not exclusively expressed in amastigotes but are also present in the epimastigote stage of *T. cruzi*.

While mass spectrometry is exquisitely sensitive, subcellular fractionations only provide partial enrichment of cellular components from contaminants. Thus, complementation of MS analysis with *in vivo* expression of tagged proteins validates proteomic data within a cellular context. Few studies adopt this approach despite the fact that contaminant proteins may represent 20% of proteins or more in subcellular MS datasets [57]. Medium-throughput tagging of proteins to validate data is growing more popular [58], but few studies to date have implemented such to verify proteomes of trypanosomatid parasites [54].

We validated our dataset by expressing a number of proteins identified in the CV proteome as GFP-fusion proteins in *T. cruzi*. We complemented this set of proteins with selected proteins with known localizations to the CV in other protists and with proteins that could potentially be present in the CV on the basis of our knowledge of the organelle. Four proteins not present in any of our mass spectrometry data (Rab11, calmodulin, VAMP1, and a phosphate transporter), were added due to roles in CV physiology in *D. discoideum* (Rab11) [19], previously suggested presence in the CV of epimastigotes (CaM) [4], or *P. tetraurelia* (VAMP1) [21], and the abundance of phosphate in the CV of epimastigotes (Pho1) [4], respectively.

The proteins we localized to the CV belong to two groups: membrane transporters and intracellular traffic regulators. V-H^+^-ATPase, which we observed in the CV bladder, is thought to play a role in water uptake by facilitating an osmotic gradient of bicarbonate in other protists [33], [35]. In *D. discoideum* it associates with the tubular network and bladder of the CV [33]. We previously reported the co-localization of antibodies against the V-H^+^-ATPase with antibodies against *T. cruzi* PMCA Ca^2+^-ATPase (Tca1) to large vacuoles and acidocalcisomes [36]. In view of our present results, those large vacuoles could correspond to the CV bladders. The localization of Tca1 [36] in the CV would suggest a role of the organelle in Ca^2+^ homeostasis, as occurs with the CV of *D. discoideum*, which also has a PMCA-type Ca^2+^-ATPase (pat1) [8], [59]. The confirmation of such a role for the CV of *T. cruzi* will necessitate further study, but it may be significant given that we also identified peptides corresponding to a calcium channel and an IP_3_/ryanodine receptor in this subcellular fraction (Table 1).

The phosphate transporter of *T. cruzi* (TcPho1) is similar to Pho91, a vacuolar phosphate transporter of *S. cerevisiae* [24]. TcPho1 is 27% identical to *S. cerevisiae* Pho91, and contains 12 transmembrane domains (TopPred II). The localization of this putative phosphate transporter to the CV is in agreement with the presence of large amounts of Pi in these organelles [4]. It is also compatible with the postulated importance of polyphosphate hydrolysis and Pi accumulation needed to increase osmotic pressure of the CV during hyposmotic stress [6]. The Pi accumulated in the CV needs to be removed during regulatory volume decrease, and the presence of a Pi/H^+^ symporter could fulfill such a role.

Proteins with putative roles in vesicle fusion and trafficking comprise the majority of the proteins for which we confirmed CV localization. The adaptor-associated protein AP180 localizes to the bladder. AP180 is a clathrin assembling protein [60]. Clathrin assembly proteins belong to one of two families, the tetrameric AP family and the monomeric AP family. Four tetrameric APs have been described and designated AP-1, AP-2, AP-3, and AP-4. Orthologues of each of these are present in the genome of *T. cruzi* [15]. AP180 is one of the members of the monomeric AP family and localizes to clathrin-coated vesicles budding from presynaptic plasma membranes [61] and the CV of *D. discoideum* [38]. In *D. discoideum* AP180 was proposed to play a role in recycling Vamp7B (which has 29% identity and 49% similarity with *T. cruzi* VAMP1) from the contractile vacuole [38]. Without AP180, Vamp7B would accumulate on CVs resulting in increased homotypic fusion with the formation of abnormally large CVs [38].

SNARE proteins are found throughout the eukaryotes and are important for vesicular fusion [62]. Two *T. cruzi* R-SNARE proteins co-localize with calmodulin to a compartment proximal to the bladder (spongiome, see below) while another R-SNARE (VAMP1) localizes to the CV bladder. These SNAREs could direct fusion of the spongiome with bladder membranes or acidocalcisomes during hyposmotic stress [4], [6]. While ceramide conjugates are typically used as markers for the Golgi complex in other systems [48], [49], we observed that SNARE2.1 co-localized with BSA-conjugated BODIPY-ceramide in *T. cruzi*. In *P. tetraurelia*, ceramide labels CV complexes and acidosomes [50]. Acidosomes have been postulated to be part of the spongiome of the contractile vacuole complex [52] or fragmented contractile vacuole membranes in *D. discoideum* [33]. Fig. 3D (arrow) also shows labeling of Golgi-like structures with antibodies against SNARE2.1-GFP, which could suggest some link between these two structures.

Rab proteins regulate CV function in *D. discoideum* [19], [32]. Of these, Rab11 is particularly important. We report that *T. cruzi* Rab11 and Rab32 are present in the CV bladder. Rab11 may mediate CV discharge in *T. cruzi* via interaction with drainin, a Rab11A effector that regulates CV discharge in *D. discoideum* [32], [63]. Both Rab32 and Rab11 partially co-localized with BODIPY-ceramide in CV bladders. Rab32 has been reported to function as an A-kinase anchoring protein in mitochondria [46] and melanosomes [47]. Rab32 may be involved in the signaling pathway leading to regulatory volume decrease in *T. cruzi* [6]. The localization of the putative phosphate transporter together with Rab32 adds two novel proteins to the protein complement of CV of all organisms.

Calmodulin (CaM) has been defined as a cytosolic Ca^2+^ receptor. TcCaM was purified from epimastigotes [64], [65] and can stimulate the PMCA Ca^2+^-ATPase [65] and cyclic AMP phosphodiesterase [64]. It has four calcium-binding sites (EF-hand domains), is 92% identical to human CaM, and is encoded by several copies in the genome [66]. Antibodies against human CaM localize to the CV [4] and to the cytosol, and this was confirmed in this work using GFP-tagged CaM.

The identification of these novel CV proteins provides useful insights into the biogenesis of these organelles. A common feature of all the validated CV proteins identified in this study is the presence of one or more tyrosine-based sorting signals with the YXXØ consensus motif (see Table S7). This sequence binds to the µ subunits of the four AP complexes [67]. In this regard, AP-1 is required for the biogenesis of the CV in *D. discoideum* [68], and AP-2 is known to interact with AP180 in bovine brain [60]. These motifs are also present in the proteins previously identified in the CV of *T. cruzi* (see Table S7). Except for AQP1, all of these proteins also have casein kinase 2 (CK2) and glycogen synthase kinase β (GSK3β) phosphorylation sites. Excepting CaM, all the proteins possess generic N-glycosylation motifs (see Table S7). It is known that a variety of kinases localize to the Golgi and regulate post-Golgi membrane trafficking [69]. These findings will help guiding future studies on the biogenesis of these organelles.

In summary, in addition to validate the expression at the protein level of a number of important genes (*DGF-1*, *calpain-like cysteine peptidases*, *amastins*) in epimastigotes, we identified nine CV proteins using a strategy complementing subcellular proteomics and bioinformatics with *in vivo* localization. Two of these proteins (Rab32, Pho1) are newly identified CV proteins, and their identification will facilitate further studies to elucidate the roles of this organelle in *T. cruzi* physiology.

## Materials and Methods

### Cell culture

*T. cruzi* epimastigotes (CL strain) were grown at 28°C in liver infusion tryptose (LIT) medium [70] supplemented with 10% heat-inactivated newborn calf serum. GFP-expressing cell lines were maintained in LIT medium supplemented with 10% heat-inactivated fetal bovine serum and G418 (Calbiochem).

### Subcellular fractionation of contractile vacuoles and 1-D gel electrophoresis

Fractions enriched in CVs were isolated as described [4] using differential and gradient centrifugation. Briefly, epimastigotes (1.4 g wet weight) were washed twice with Buffer A (116 mM NaCl, 5.4 mM KCl, 0.8 mM MgSO_4_, 50 mM HEPES, pH 7.2) with 5.5 mM glucose. The parasites were washed once in cold lysis buffer (120 mM sucrose, 50 mM KCl, 4 mM MgCl, 0.5 mM EDTA, 20 mM HEPES, 5 mM DTT, 0.2% Sigma mammalian protease inhibitor cocktail, pH 7.2) prior to lysis with silicon carbide in lysis buffer. Silicon carbide and cell debris was eliminated by a series of low speed centrifugations (38 *g*, 144 *g*, and 1,200 *g*). The supernatant was centrifuged at 100,000 *g* for 60 min, and the pellet was resuspended in 2 ml lysis buffer and applied to the 25% step of a discontinuous gradient of iodixanol, with 4 ml steps of 15, 20, 25, 30, 34, 37 and 40% iodixanol, diluted in lysis buffer. The gradient was centrifuged at 50,000 g in a Beckman JS-24.38 rotor for 65 min and fractions were collected from the top. Two hundred microliters were reserved for quantification of protein by Bradford assay and marker enzyme assays, as described before [4]. The first 4.5 ml from the top of the gradient were centrifuged at 100,000 g for 30 min and the pellet used for proteomic analysis. The final pellet was resuspended in Laemmli buffer (Sigma-Aldrich) and heated at 80°C for 15 min. Solubilized proteins were separated on a Nu-PAGE 4-12% Bis-Tris (Invitrogen) gradient gel at 150 V for 2 h.

### In-gel digestion

The gel lane was washed twice in ddH_2_O for 15 min and cut into 7 equal slices. Proteins were reduced with 10 mM DTT/100 mM Ambic (ammonium bicarbonate) solution at 57°C for 1 h and carboxyamidomethylated with 55 mM iodoacetamide and 100 mM Ambic for 1 h at room temperature in the dark. Enzymatic digestion was performed with porcine trypsin (1∶50, Promega, Madison, WI) at 37°C overnight. Tryptic peptides were extracted three times with 200 µl of 50% ACN (1∶1 in water). Combined extracts were dried in a speed vacuum, resuspended in 50 µl 0.1% formic acid, and stored at −20°C.

### Mass spectrometry

Prior to LC-MS/MS, trypsin was removed by centrifugal ultrafiltration (MWCO, 30 kDa; Millipore), and peptides were analyzed on an Agilent 1100 capillary LC (Palo Alto, CA) coupled to a LTQ linear ion trap mass spectrometer (Thermo Electron). Mobile phases A and B were H_2_O/0.1% formic acid and ACN/0.1% formic acid, respectively. Fractions were loaded for 1 h onto a PicoFrit 8 cm ×50 µm column (New Objective) packed with 5 µm C18 beads under positive N_2_ pressure. Peptides were desalted for 10 min with 0.1% formic acid using positive N_2_ pressure and eluted into the mass spectrometer during a 70 min linear gradient from 5–45% B at a flow rate of 200 nL min^−1^. The spectrometer acquired MS/MS spectra on the nine most abundant precursor ions from each scan with a repeat count of three and repeat duration of 15 sec. Dynamic exclusion was enabled for 160 sec. Raw tandem mass spectra were converted into mzXML format and peak lists using ReAdW and mzMXL2Other [71]. Peak lists were searched using Mascot v1.9 software (Matrix Science, Boston, MA) and two databases were constructed. The first database (normal) was composed of *T. cruzi* gene annotations provided by GeneDB (GeneDB.org). A second decoy (random) database was constructed by reversing sequences from the normal database. Database searches were performed against normal and random databases using the following parameters: full tryptic enzymatic cleavage with three possible missed cleavages, peptide tolerance of 1,000 ppm, fragment ion tolerance of 0.6 Da, and variable carboxyamidomethylation (+57 Da) modification. Peptide matches were extracted from the normal and reverse databases. Protein false-discovery rates (PRO-FDR) were calculated using the ProValt algorithm in ProteoIQ (BioInquire, Athens, GA) [72]. ProValt parsimoniously clusters nonredundant peptides falling within user-defined criteria to protein homology groups based upon sequence homology. ProValt returns the protein with highest sequence coverage as the top scoring protein. Proteins identified below a 1% false protein discovery rate were considered significant. Prior to additional refinement, we manually screened all protein hits to ensure we would not overlook potentially valuable data (i.e. hits with homology to known CV proteins in other protists) that could be filtered out by more conservative analysis. To increase confidence in protein identification, the dataset (1% false protein discovery rate) was filtered by a protein group probability of 0.95 using the ProteinProphet algorithm [73].

### Bioinformatic analysis of mass spectrometry results

Though subcellular enrichment increases representation of organellar proteins, organellar proteomic datasets invariably include contaminants from other compartments due to high sensitivity of mass spectrometry and limitations of fractionation protocols. While no known motifs or structures target proteins to the CV of *T. cruzi*, we used the following localization prediction algorithms to identify contaminant proteins from other cellular compartments: targetP 1.1 [74], pTARGET [75], WoLfPsort [76], SLP-LOCAL [77], PA-SUB [78]. Perl scripts were used to filter data with prediction confidence thresholds of 80%. Final predictions of localization were made where two or more algorithms agreed. Signal peptides and membrane topology were predicted with SignalP3 [79], TMHMM2.0c [80], HMMTOP2.1 [81] and PolyPhobius [82], [83] (accessed April 10, 2009). Empirical localization data from literature were collated for annotated proteins.

### Confirmation of localization with GFP-fusion proteins

Over-expression constructs for genes encoding putative CV proteins were subcloned from *T. cruzi* CL Brener. Primers (see Table S8) were designed to insert sequences into C-terminal or N-terminal GFP-fusion plasmids derived from the *T. cruzi* shuttle vectors pTEX [84] or pTREX [85]. We designed a new vector (pTEX-GFPN) for expressing N-terminal GFP fusion proteins in *T. cruzi* by insertion of GFP into pTEX between the SpeI and BamHI restriction sites (see Fig. S2). All *T. cruzi* sequences were verified by sequencing (Yale DNA Analysis Facility, Yale University, New Haven, Connecticut). *T. cruzi* CL or Y strain epimastigotes (10^8^) were transfected in cytomix (120 mM KCl, 0.15 mM CaCl_2_, 10 mM K_2_HPO_4_, 2 mM EDTA, 5 mM MgCl_2_, pH 7.6) containing 80 µg of each plasmid construct in 2 mm electroporation cuvettes with 3 pulses (300 V, 500 µF) delivered by a Gene Pulser II (Bio-Rad), and expression of GFP-fusion proteins was verified by western blot analyses. Stable cell lines were established under drug selection with G418 at 250 µg ml^−1^.

GFP-Rab32, GFP-Pho1, GFP-CaM and GFP-VAMP1 cell lines were established as above with some modifications. Electroporation was performed in cytomix supplemented with 25 mM HEPES using 50–100 µg plasmid DNA in a 4 mm cuvette. The cuvette was cooled on ice for 10 min and pulsed 3 times (1.5 kV, 25 µF) with a Gene Pulser Xcell™ (Bio-Rad). Cuvettes were kept at room temperature for 15 min, and the parasites transferred to 5 ml of LIT medium containing 20% newborn calf serum. Stable cell lines were established under drug selection with G418 at 200 µg ml^−1^. Enrichment of GFP-fluorescent parasites was performed with a high speed cell sorter when needed (MoFlo Legacy; Beckman-Coulter, Hialeah, FL).

### Western blot analyses

For western blot analyses of *T. cruzi* soluble and membrane fractions, *T. cruzi* epimastigotes (∼10^8^) were washed twice with PBS (pH 7.4) and resuspended in 50 mM Tris-HCl (pH 7.4) containing protease inhibitors (Sigma P8340, diluted 1∶250), 2 mM EDTA, 2 mM PMSF, 2 mM TPCK and 0.1 mM E64. The cells were lysed with three cycles of freezing (5 min, liquid N_2_) and thawing (1 min, 37°). Lysed cells were centrifuged for 1 h at 100,000 *g* at 4°C to separate soluble (supernatant) and membrane-associated (pellet) fractions. The membrane-associated protein was resuspended in modified RIPA buffer (150 mM NaCl, 20 mM Tris-Cl pH 7.5, 1 mM EDTA, 1% SDS and 0.1% Triton x-100). For the detection of *T. cruzi* Pho1, the samples were loaded directly without incubation at 95°C. Proteins were separated by SDS-PAGE and transferred to nitrocellulose. Western blot analysis was performed in PBS-T (PBS plus 0.1% Tween 20). Membranes were blocked overnight in 5% nonfat dry milk prior to blotting with polyclonal anti-GFP antibody (diluted 1∶10,000, Invitrogen) and horseradish peroxidase-labeled anti-rabbit IgG. The blots were developed with ECL reagent (Pierce). Epimastigote whole homogenates were prepared in modified RIPA buffer, incubated on ice for 1 h and electrophoresed as described before.

GFP-Rab32 and GFP-Rab11 cell lines were prepared similarly but with the following modifications. Epimastigotes were harvested, washed 3 times in cold PBS, and lysed for 4 hours at 4°C in 500 µl radioimmunoprecipitation analysis (RIPA) buffer (50 mM Tris-HCl, pH 7.4, 150 mM NaCl, 0.5% Nonidet P-40, 0.5% sodium deoxycholate, 0.1% SDS 1 mM EGTA, and 1 mM MgCl_2_) containing protease inhibitors (Sigma P8340, diluted 1∶250). Protein (50 µg) was separated using 4%–20% gradient Ready Gels (Bio-Rad) and blotted onto nitrocellulose. Subsequent processing steps were done in PBS containing 0.1% Tween 20. Blots were blocked overnight at 4°C with 2% BSA prior to labeling with polyclonal anti-GFP antibody (diluted 1∶7500) and horseradish peroxidase-labeled anti-rabbit IgG.

### Fluorescence Microscopy

We directly observed subcellular localization of GFP-fusion proteins in epimastigotes under isosmotic (300 mOsm) and hyposmotic conditions (150 mOsm). We prepared cells for observation under hyposmotic stress by washing twice in PBS (pH 7.4) and resuspending them in PBS or isotonic chloride buffer (iso-Cl[86]). The osmolality of iso-Cl buffer was adjusted to 300 mOsm using a 3D3 osmometer (Advanced Instruments, Norwood). Hyposmotic stress (150 mOsm) was induced by addition of an equal volume of deionized water to cell suspensions.

For immunofluorescence microscopy, cells were fixed in PBS (pH 7.4) with 4% paraformaldehyde, adhered to poly-lysine coverslips, and permeabilized for 5 min with PBS (pH 7.4) containing 0.3% Triton X-100. Permeabilized cells were blocked 1 hr in PBS (pH 7.4) containing 3% bovine serum albumin, 1% fish gelatin (Sigma), 5% goat serum, and 50 mM NH_4_Cl. GFP was labeled with a monoclonal anti-GFP antibody (3E6, 1∶300 dilution, Invitrogen) and goat anti-mouse Alexa conjugated secondary antibody (1∶2,000 dilution, Invitrogen). Calmodulin (CaM) was labeled with goat anti-CaM antibody (1∶500 dilution, Santa Cruz Biotechnology) and rabbit α-goat Alexa conjugated secondary antibody (1∶2,000 dilution, Invitrogen). For Pho1, CaM and VAMP1 cell lines, the parasites were fixed and permeabilized as described. After blocking overnight at 4°C in 3% bovine serum albumin (PBS pH 8), the cells were incubated with rabbit polyclonal anti-GFP antibody (1∶2000 dilution, Invitrogen) and goat anti-rabbit Alexa conjugated secondary antibody (1∶2000 dilution, Invitrogen).Specimens were imaged using a Delta Vision deconvolution microscope (Applied Precision).

To label cells with BODIPY®-ceramide complexed to BSA (Invitrogen), ∼10^7^ mid-log phase epimastigotes were washed 3 times in 1 ml of cold PBS and resuspended in 150 µl of cold LIT medium without serum. The cells were incubated on ice for 1 hour with 5 µM BODIPY® TR C_5_-ceramide complexed to BSA (Invitrogen). After incubation with the dye, cells were washed 3 times with 1 ml of cold LIT medium without serum and resuspended in 150 µl of the same medium pre-warmed to 37°C. Cells were incubated 1 hour at 37°C to allow for dye uptake prior to fixation in PBS containing 4% paraformaldehyde. Following fixation, cells were washed in PBS, adhered to poly-lysine coverslips, and imaged using an Olympus IX-71 fluorescence microscope coupled with a Photometrix CoolSnap_HQ_ CCD (charge-coupled device) camera driven by Delta Vision software (Applied Precision), and images were deconvolved when indicated in the figure legends.

### Electron Microscopy

*T.cruzi* epimastigotes over-expressing AP180-GFP or SNARE 2.1-GFP were washed twice in 0.1 M sodium cacodylate buffer, pH 7.4, and fixed for 1 h on ice with 0.1% glutaraldehyde, 4% paraformaldehyde and 0.1 M sodium cacodylate buffer, pH 7.4. Samples were processed for cryo-immunoelectron microscopy at the Molecular Microbiology Imaging Facility, Washington University School of Medicine. GFP-fusion protein localization was detected with a polyclonal antibody against GFP (Invitrogen) and anti-rabbit gold conjugated as a secondary antibody.

## Supporting Information

Table S1 ***Trypanosoma cruzi*** **Proteins identified from a fraction enriched in contractile vacuoles.** This Table lists all proteins identified with a 1% false discovery rate and a total protein probabilities >0.95. The gel slice in which each protein was identified is indicated by A–G. The approximate MW ranges of each slice correspond to; (A) 35–145, (B) 45–132, (C) 56–100, (D) 71–112, (E) 24.5–49, (F) 19–50, and (G) 12–36, as calculated by the MW of the proteins identified at the 25^th^ and 75^th^ percentile (ranked by calculated MW) in each slice. The appearance of the same protein in multiple bands presumably results from these proteins being partially degraded and thus appearing at a lower MW than expected, or being modified in some manner to give them a higher MW than that calculated based solely on amino acid composition.
(PDF)

Table S2 **Peptide list for all proteins matched to mass spectra from the contractile vacuole data set.** This Table includes all proteins with above 1% false discovery rate and a total protein probability > 0.95.
(PDF)

Table S3 **Peptides identified in the proteomic analysis of the subcellular fraction that map to DGF-1 proteins in epimastigotes.** This Table lists the peptides identified in the proteomic analysis that map to DGF-1 proteins in epimastigotes.
(PDF)

Table S4 **Signal peptide (SP) and transmembrane domain (TM) predictions for proteins in the T. cruzi CV dataset.** This Table lists SP and TM predictions for proteins in the CV dataset.
(PDF)

Table S5 **Annotated proteins identified by mass spectrometry in enriched CV fractions.** This Table lists the known proteins identified in CV fractions according to their potential function.
(PDF)

Table S6 **Predicted subcellular locations for proteins in the T. cruzi CV fraction from five targeting prediction servers.** This Table includes a list of high confidence (1% false discovery rate, protein group probability >0.95) and low confidence spectral matches curated from 1% false discovery rate dataset guided by CV literature.
(PDF)

Table S7 **Common features of CV proteins identified by the ELM server.** This Table list common features of proteins identified in the CV fraction.
(PDF)

Table S8 Primers used to generate expression constructs of contractile vacuole proteins. This Table includes all primers used in this work.
(PDF)

Figure S1 **Immunofluorescence microscopy of AP180.** **A.** DIC. **B.** AP180-GFP. **C.** Calmodulin. **D.** Merge. AP180-GFP is shown in green, calmodulin in red, and DAPI in blue. Scale bars  =  5 µm.
(PDF)

Figure S2 **Map of vector GFP-pTEX, an N-terminal GFP fusion vector for** ***T. cruzi***. This figure shows that the GFP gene was inserted between SpeI and BamHI. GFP is in frame with both SpeI and BamHI.
(PDF)

Movie S1 **Rab11 and BODIPY-ceramide co-localization.** Images of labeled cells (Fig. 4C) were captured in the channels blue, red and green by Z-sectioning in order to obtain 25 optical sectioned images with 0.2 µm of optical section space between each one, covering 5 µm of sample thickness. Images were then deconvolved using the softWorx toolbar. Deconvolved sections were grouped to give a volume perspective of the labeling using the volume viewer tool (softWorx tool bar) and finally saved as a movie showing a rotation of 180 degrees around the Y axis. Imunofluorescence techniques are described in material & methods section. Scale bar  =  10 µm.
(MOV)

Movie S2 **Rab32 and BODIPY-ceramide co-localization**. Images of labeled cells (Fig. 4D) were captured and processed as in Movie S1.
(MOV)
