# Supplementary material for: Identification of Contractile Vacuole Proteins in Trypanosoma cruzi
Source: PLoS One. 2011 Mar 18;6(3):e18013. doi: 10.1371/journal.pone.0018013 (PMC3060929; doi:10.1371/journal.pone.0018013)
Supplement: Table S1 — Trypanosoma cruzi Proteins identified from a fraction enriched in contractile vacuoles. This Table lists all proteins identified with a 1% false discovery rate and a total protein probabilities >0.95. The gel slice in which each protein was identified is indicated by A–G. The approximate MW ranges of each slice correspond to; (A) 35–145, (B) 45–132, (C) 56–100, (D) 71–112, (E) 24.5–49, (F) 19–50, and (G) 12–36, as calculated by the MW of the proteins identified at the 25th and 75th percentile (ranked by calculated MW) in each slice. The appearance of the same protein in multiple bands presumably results from these proteins being partially degraded and thus appearing at a lower MW than expected, or being modified in some manner to give them a higher MW than that calculated based solely on amino acid composition. (PDF) [file pone.0018013.s001.pdf]

Supplementary Table 1. *Trypanosoma cruzi* proteins identified from a fraction enriched in contractile vacuoles. This Table lists all proteins identified with a 1% false discovery rate and a total protein probabilities >0.95. The gel slice in which each protein was identified is indicated by A-G. The approximate MW ranges of each slice correspond to: (A) 35-145, (B) 45-132, (C) 56-100, (D) 71-112, (E) 24.5-49, (F) 19-50, and (G) 12-36, as calculated by the MW of the proteins identified at the 25th and 75th percentile (ranked by calculated MW) in each slice. The appearance of the same protein in multiple bands presumably results from these proteins being partially degraded and thus appearing at a lower MW than expected, or being modified in some manner to give them a higher MW than that calculated based solely on amino acid composition.

GeneID	Name	Length	Size	Gel Slice	Probability	Total Score	Total Spectra	Total Total Peptides	Total Peptides Paris Guidelines	% Coverage
Tc00.1047053510101.140	pyruvate phosphate dikinase, putative [8305.t00014]	914	100.77	A-C	1.00	2265.54	384	40	40	49
Tc00.1047053506297.190	pyruvate phosphate dikinase, putative [6890.t00019]	914	100.75	A-C,E,G	1.00	2179.24	382	39	39	48
Tc00.1047053507029.30	heat shock 70 kDa protein, mitochondrial precursor, putative [7180.t00003]	656	70.93	A-C,F	1.00	1391.92	81	20	20	37
Tc00.1047053507641.290	chaperonin HSP60, mitochondrial precursor [7414.t00029]	560	59.11	C	1.00	1167.04	59	16	16	44
Tc00.1047053506839.30	hypothetical protein, conserved [7112.t00003]	1080	119.41	B	1.00	814.13	40	13	13	17
Tc00.1047053506585.40	glucose-regulated protein 78, putative [7009.t00004]	652	71.25	A-C	1.00	719.57	51	9	9	21
Tc00.1047053506247.220	histidine ammonia-lyase, putative [6869.t00022]	535	58.03	C	1.00	644.87	31	10	10	28
Tc00.1047053510215.10	NADH-dependent fumarate reductase, putative [5865.t00001]	1143	124.70	B	1.00	627.77	42	10	10	11
Tc00.1047053508741.229	hypothetical protein, conserved [7853.t00045]	273	29.60	A-B,F	1.00	624.18	42	11	11	50
Tc00.1047053511215.119	69 kDa paraflagellar rod protein, putative [8623.t00012]	601	69.50	C	1.00	611.68	24	9	9	25
Tc00.1047053505763.19	P-type H+-ATPase, putative [6697.t00002]	646	70.69	A-C	1.00	560.76	34	8	8	16
Tc00.1047053508737.210	hypothetical protein, conserved [7851.t00021]	273	29.60	A,F-G	1.00	556.34	36	10	10	45
Tc00.1047053506943.50	glyceraldehyde 3-phosphate dehydrogenase, putative [7148.t00005]	360	38.99	A,F-G	1.00	518.08	19	9	9	36
Tc00.1047053509445.39	glutamate dehydrogenase, putative [8102.t00004]	384	43.23	A-C	1.00	512	41	10	10	28
Tc00.1047053505843.10	glutamate dehydrogenase, putative [6721.t00001]	212	24.09	A	1.00	506.38	45	11	11	49
Tc00.1047053511903.40	hypothetical protein, conserved [8822.t00004]	500	54.70	C	1.00	492.03	34	9	9	24
Tc00.1047053503849.60	NADH-dependent fumarate reductase, putative [4783.t00006]	790	85.42	B	1.00	481.23	21	7	7	13
Tc00.1047053509237.130	cytoskeleton-associated protein CAP5.5, putative [8046.t00013]	798	88.59	A-C	1.00	479.13	13	7	7	14
Tc00.1047053507547.90	glycosomal phosphoenolpyruvate carboxykinase, putative [7378.t00009]	526	58.82	C,F	1.00	467.77	22	8	8	20
Tc00.1047053411235.9	alpha tubulin, putative [11788.t00001]	452	49.75	B,F-G	1.00	464.4	42	5	5	17
Tc00.1047053510943.50	delta-1-pyrroline-5-carboxylate dehydrogenase, putative [8550.t00005]	562	62.42	A-C	1.00	464.03	14	7	7	17
Tc00.1047053509129.10	hypothetical protein, conserved [8009.t00001]	273	29.59	A,E-F	1.00	454.51	30	8	8	36
Tc00.1047053504087.20	hypothetical protein [4902.t00002]	2984	323.02	A-B,F	1.00	449.58	17	8	8	4
Tc00.1047053508535.10	NADH-dependent fumarate reductase, putative [7767.t00001]	1216	132.28	B	1.00	447.62	16	8	8	10
Tc00.1047053511277.290	aconitase, putative [8643.t00029]	899	98.63	C	1.00	440.73	10	7	7	11
Tc00.1047053509683.10	hypothetical protein [8167.t00001]	2984	323.75	A,F-G	1.00	431.53	16	8	8	4
Tc00.1047053509617.20	paraflagellar rod protein 3, putative [8152.t00002]	590	68.60	A-C	1.00	418.31	25	6	6	13
Tc00.1047053508555.60	cytoskeleton-associated protein CAP5.5, putative [7777.t00006]	807	89.73	A-C	1.00	418.13	12	6	6	10
Tc00.1047053506211.160	ADP,ATP carrier protein 1, mitochondrial precursor, putative [6853.t00016]	315	34.91	A,E-F	1.00	400.64	20	6	6	26
Tc00.1047053510149.80	ABC transporter, putative [8319.t00008]	1866	206.41	A	1.00	398.92	10	8	8	7
Tc00.1047053509351.10	delta-1-pyrroline-5-carboxylate dehydrogenase, putative [5692.t00001]	266	29.48	A-C	1.00	394.81	11	6	6	31
Tc00.1047053506491.20	myosin heavy chain, putative [6968.t00002]	3544	398.66	A,F-G	1.00	391.44	23	8	8	2
Tc00.1047053504153.310	heat shock protein, putative [4935.t00031]	751	83.84	A-C	1.00	386.52	19	6	6	11
Tc00.1047053511909.40	succinate dehydrogenase flavoprotein, putative [6223.t00004]	610	66.84	C	1.00	385.23	8	6	6	14
Tc00.1047053507089.270	dihydrolipoyl dehydrogenase, putative [7204.t00027]	478	50.46	A,E-F	1.00	385.07	13	7	7	23
Tc00.1047053506337.70	2-oxoglutarate dehydrogenase E1 component, putative [6903.t00007]	1007	112.28	A-C	1.00	379.93	5	5	5	8
Tc00.1047053509999.90	carnitine/choline acetyltransferase, putative [5825.t00009]	611	66.93	C	1.00	359.55	12	5	5	14
Tc00.1047053510837.20	glutamamyl carboxypeptidase, putative [8516.t00002]	397	43.49	A,F-G	1.00	347.65	31	6	6	21
Tc00.1047053507689.30	glutamamyl carboxypeptidase, putative [7435.t00003]	397	43.52	A-C, E-G	1.00	347.49	31	6	6	21
Tc00.1047053509551.30	mitochondrial phosphate transporter, putative [5738.t00003]	317	34.32	A-B	1.00	344.88	12	6	6	22
Tc00.1047053508153.340	6-phospho-1-fructokinase, putative [7617.t00034]	486	53.51	A,F-G	1.00	343.44	7	5	5	13
Tc00.1047053511289.70	ADP,ATP carrier protein 1, mitochondrial precursor, putative [8647.t00007]	315	34.93	A,F-G	0.96	328.51	18	6	6	25
Tc00.1047053509499.14	tryparedoxin peroxidase, putative [8115.t00003]	227	25.47	A	1.00	326.85	9	5	5	25
Tc00.1047053503899.119	trypanothione/tryparedoxin dependent peroxidase 2, putative [4808.t00014]	178	19.70	A,C,E-F	1.00	323.86	14	6	6	34
Tc00.1047053509961.70	dispersed gene family protein 1 (DGF-1, pseudogene), putative [8255.t00007]	3017	311.64	A,F-G	1.00	322.89	10	8	8	3
Tc00.1047053509585.10	dynein heavy chain, putative [8142.t00001]	3707	423.67	A,C	1.00	321.59	8	5	5	2
Tc00.1047053510679.40	hypothetical protein [8462.t00004]	456	52.17	A	1.00	298.88	39	4	4	13
Tc00.1047053508209.100	10 kDa heat shock protein, putative [7639.t00010]	101	10.68	G	1.00	291.7	14	4	4	50
Tc00.1047053508209.120	10 kDa heat shock protein, putative [7639.t00012]	101	10.67	G	1.00	284.36	10	4	4	50
Tc00.1047053507187.9	dispersed gene family protein 1 (DGF-1), putative [7243.t00001]	1748	182.88	A,F-G	1.00	271.15	6	5	5	4
Tc00.1047053506249.70	ABC transporter, putative [6870.t00007]	666	74.56	A	1.00	268.34	6	4	4	9
Tc00.1047053508827.40	acyl-CoA dehydrogenase, putative [7887.t00004]	625	68.61	A-C	1.00	267.84	5	3	3	6
Tc00.1047053508741.170	hypothetical protein, conserved [7853.t00017]	526	56.06	C	1.00	244.87	6	3	3	10
Tc00.1047053509463.30	3-ketoacyl-CoA thiolase, putative [8107.t00003]	438	46.36	A,F-G	1.00	244.29	7	4	4	15
Tc00.1047053510797.30	hypothetical protein, conserved [8501.t00003]	760	87.03	C	1.00	242.21	9	4	4	8
Tc00.1047053508177.10	hypothetical protein, conserved [7626.t00001]	631	67.82	A	1.00	237.84	5	4	4	8
Tc00.1047053511441.10	calpain cysteine peptidase, putative [6119.t00001]	1276	145.12	A	1.00	237.21	17	6	6	5
Tc00.1047053504949.30	succinate dehydrogenase, putative [6451.t00003]	187	21.32	B,F-G	1.00	233.38	17	5	5	34
Tc00.1047053506445.60	mitochondrial DNA topoisomerase II, putative [6947.t00006]	1231	138.29	B	1.00	233.32	5	3	3	3
Tc00.1047053511531.50	glucosamine-6-phosphate isomerase, putative [6143.t00005]	280	31.40	A,F-G	1.00	230.89	5	4	4	20
Tc00.1047053506563.40	beta tubulin, putative [6998.t00004]	443	49.65	A-C	1.00	230.87	17	4	4	11
Tc00.1047053507009.10	Gim5A protein, putative [7172.t00001]	227	24.49	A,C,E-F	1.00	226.1	13	4	4	22
Tc00.1047053509701.10	trifunctional enzyme alpha subunit, mitochondrial precursor-like protein, putative [8174.t00001]	688	75.62	C	1.00	226.06	11	3	3	8
Tc00.1047053510395.10	ATP synthase, alpha chain, mitochondrial precursor, putative [5909.t00001]	248	25.82	A	1.00	216.41	10	3	3	15
Tc00.1047053507927.20	mitochondrial oligo_U binding protein TBRGG1, putative [7531.t00002]	890	97.19	A-C	1.00	211.69	4	4	4	8
Tc00.1047053509777.130	hypothetical protein, conserved [8200.t00013]	598	66.83	A	1.00	210.32	8	3	3	7
Tc00.1047053509215.40	cyclophilin, putative [8039.t00004]	211	22.89	B,F-G	1.00	206.46	28	5	5	24
Tc00.1047053511575.130	folate/pteridine transporter, putative [8726.t00013]	630	69.57	A,F-G	1.00	205.67	4	3	3	7
Tc00.1047053510773.20	vacuolar-type proton translocating pyrophosphatase 1, putative [8493.t00002]	815	85.26	A	1.00	204.71	12	3	3	5

Tc00.1047053508707.310	hypothetical protein, conserved [7839.t00031]	915	101.77	A,F-G	1.00	197.42	7	5	5	8
Tc00.1047053506577.120	sterol C-24 reductase, putative [7005.t00012]	489	57.72	A	1.00	197.36	5	4	4	9
Tc00.1047053508737.100	ATP-dependent Clp protease subunit, heat shock protein 78, putative [7851.t00010]	803	89.99	C	1.00	196.61	7	4	4	7
Tc00.1047053510003.20	hypothetical protein, conserved [8269.t00002]	1246	137.81	B	1.00	194.72	17	4	4	3
Tc00.1047053508815.179	dynein heavy chain, putative [7882.t00018]	2460	282.16	A,C,E	1.00	194.34	5	4	4	2
Tc00.1047053508503.20	cytochrome c oxidase subunit V, putative [7754.t00002]	197	22.22	B,F-G	1.00	190.87	4	3	3	18
Tc00.1047053506275.20	hslvu complex proteolytic subunit-like, putative [6881.t00002]	210	22.85	A,F-G	1.00	188.39	7	4	4	20
Tc00.1047053509799.140	hypothetical protein, conserved [5786.t00014]	589	65.46	A,C,E	1.00	188.32	8	4	4	8
Tc00.1047053511389.150	thiolase protein-like protein, putative [8680.t00015]	448	47.99	A	1.00	183.02	5	4	4	13
Tc00.1047053506223.80	ATP-dependent zinc metallopeptidase, putative [6858.t00008]	684	75.74	A-C	1.00	179.89	6	3	3	7
Tc00.1047053506563.79	calpain-like cysteine peptidase (pseudogene), putative [6998.t00023]	132	14.87	B,F-G	1.00	167.26	12	3	3	22
Tc00.1047053509911.74	hypothetical protein, conserved [8242.t00014]	196	21.75	B,F-G	1.00	163.45	5	3	3	18
Tc00.1047053511277.170	ATP-dependent zinc metallopeptidase, putative [8643.t00017]	565	61.91	C	1.00	162.95	3	2	2	8
Tc00.1047053507941.150	histone H4, putative [7536.t00015]	101	11.15	G	1.00	160.47	8	3	3	31
Tc00.1047053508981.39	trifunctional enzyme alpha subunit, mitochondrial precursor-like protein [7949.t00004]	692	75.40	A-C	0.99	159.49	3	2	2	5
Tc00.1047053506989.190	lipophosphoglycan biosynthetic protein, putative [7164.t00019]	763	86.95	C	1.00	157.14	5	3	3	5
Tc00.1047053511217.90	hypothetical protein, conserved [8624.t00009]	1828	206.10	B	1.00	151.15	6	4	4	3
Tc00.1047053507501.10	retrotransposon hot spot (RHS) protein, putative [7361.t00001]	914	103.51	A-C	1.00	151.09	5	3	3	3
Tc00.1047053508231.40	hypothetical protein, conserved [7648.t00004]	259	29.34	A,C,E	1.00	145.1	10	2	2	9
Tc00.1047053506551.10	hypothetical protein, conserved [5375.t00001]	618	68.53	A	1.00	144.76	3	2	2	4
Tc00.1047053506563.170	calpain-like cysteine peptidase (pseudogene), putative [6998.t00017]	131	14.64	G	1.00	143.51	9	3	3	20
Tc00.1047053508241.130	hypothetical protein, conserved [7652.t00013]	1084	120.46	B	1.00	141.78	3	3	3	4
Tc00.1047053508999.250	calpain-like cysteine peptidase (pseudogene), putative [7958.t00025]	141	15.80	B,F-G	1.00	141.76	11	3	3	28
Tc00.1047053511211.160	heat shock protein 70 (HSP70), putative [8621.t00016]	655	70.87	C	1.00	139.65	5	3	3	8
Tc00.1047053504147.70	hypothetical protein, conserved [4932.t00007]	163	17.77	F	1.00	139.01	4	3	3	26
Tc00.1047053507093.260	ABC transporter, putative [7206.t00026]	662	72.71	A	1.00	135.6	6	2	2	3
Tc00.1047053505945.20	ribonuclease mar1, putative [6745.t00002]	197	21.40	B,F-G	1.00	134.99	19	2	2	13
Tc00.1047053510533.210	hypothetical protein, conserved (pseudogene) [8421.t00021]	2179	255.02	A	1.00	134.21	17	3	3	2
Tc00.1047053509203.40	glycosomal membrane protein, putative [8035.t00004]	219	23.84	F	1.00	132.89	5	3	3	12
Tc00.1047053507711.200	hypothetical protein, conserved [7442.t00020]	1547	167.96	B	1.00	130.78	3	3	3	2
Tc00.1047053510353.30	paraflagellar rod component Par4, putative [5902.t00003]	581	68.17	A-C	1.00	127.58	3	2	2	5
Tc00.1047053508723.70	hypothetical protein, conserved [7844.t00007]	2201	248.56	A,C,E	1.00	126.4	4	3	3	2
Tc00.1047053511003.190	hypothetical protein, conserved [8565.t00019]	292	32.49	A	1.00	126.02	7	3	3	12
Tc00.1047053506147.80	hypothetical protein, conserved [6829.t00008]	169	18.98	F	1.00	125.19	1	1	1	13
Tc00.1047053457251.10	3-oxo-5-alpha-steroid 4-dehydrogenase, putative [3262.t00001]	264	29.57	A	1.00	125	8	3	3	13
Tc00.1047053509797.40	isoleucyl-tRNA synthetase, putative [8206.t00004]	1157	132.28	B	1.00	120.13	2	2	2	3
Tc00.1047053506755.20	paraflagellar rod component, putative [7083.t00002]	593	68.21	C	1.00	118.91	2	2	2	7
Tc00.1047053508647.200	triosephosphate isomerase, putative [7814.t00020]	252	27.27	E	1.00	117.26	2	2	2	9
Tc00.1047053506839.70	NADH dehydrogenase, putative [7112.t00007]	513	56.38	A,C,E	1.00	116.16	5	2	2	5
Tc00.1047053506201.170	hypothetical protein, conserved [6849.t00017]	839	96.75	A,C,E	0.97	115.32	4	3	3	4
Tc00.1047053506977.60	hypothetical protein, conserved [7160.t00006]	116	12.37	G	1.00	112.17	1	1	1	16
Tc00.1047053503837.10	hypothetical protein, conserved [4777.t00001]	605	66.40	A,E	1.00	111.92	2	1	1	3
Tc00.1047053507711.60	hypothetical protein, conserved [7442.t00006]	872	101.81	B	1.00	111.45	1	1	1	2
Tc00.1047053509051.20	amastin, putative [7979.t00002]	179	19.56	A,C,E	1.00	110.2	3	2	2	13
Tc00.1047053503793.10	2-oxoglutarate dehydrogenase subunit, putative [4755.t00001]	338	37.98	B	1.00	106.17	1	1	1	6
Tc00.1047053504069.80	ATP synthase F1 subunit gamma protein, putative [4893.t00008]	307	34.59	B,F	1.00	105.97	2	1	1	5
Tc00.1047053509233.180	ATPase beta subunit, putative [8045.t00018]	519	55.68	C	1.00	102.35	2	1	1	3
Tc00.1047053504089.50	flagellar radial spoke component, putative [4903.t00005]	597	67.59	A-C	1.00	101.58	4	2	2	5
Tc00.1047053509965.394	amastin, putative [8257.t00048]	186	20.30	A,C,E	0.99	97.96	10	3	3	15
Tc00.1047053509679.9	leucyl-tRNA synthetase, putative [5766.t00001]	270	30.97	B	1.00	97	3	2	2	10
Tc00.1047053506355.10	hexose transporter, putative [6911.t00001]	545	58.58	A,C-D	1.00	96.94	3	2	2	4
Tc00.1047053506657.40	ADP/ATP translocase, putative [7042.t00004]	344	38.05	A,C,E	1.00	96.94	3	2	2	7
Tc00.1047053506587.70	hypothetical protein, conserved [7010.t00007]	282	31.34	A	1.00	95.33	6	2	2	9
Tc00.1047053511809.130	40S ribosomal protein S15, putative [8793.t00013]	153	17.49	G	1.00	94.95	1	1	1	11
Tc00.1047053503893.30	hypothetical protein, conserved [4805.t00003]	170	20.20	B,F-G	0.98	94.95	26	3	3	17
Tc00.1047053511733.90	hypothetical protein, conserved [8767.t00009]	117	13.03	G	1.00	93.05	1	1	1	15
Tc00.1047053503769.40	cytochrome c oxidase VII, putative [4743.t00004]	166	19.02	F	1.00	92.27	6	2	2	11
Tc00.1047053508173.264	hypothetical protein, conserved [7624.t00033]	312	33.71	A,C,E	1.00	89.45	3	2	2	7
Tc00.1047053510855.10	peptide methionine sulfoxide reductase, putative [8524.t00001]	178	20.06	G	1.00	89.13	1	1	1	8
Tc00.1047053503413.4	hypothetical protein, conserved [4565.t00002]	999	108.74	B	1.00	88.98	1	1	1	2
Tc00.1047053511773.110	retrotransposon hot spot (RHS) protein, putative [6198.t00011]	824	93.04	C	1.00	88.58	2	1	1	2
Tc00.1047053506025.14	ribosomal protein S29, putative [5326.t00008]	58	6.62	G	1.00	87.69	4	2	2	34
Tc00.1047053508045.70	hypothetical protein, conserved [7579.t00007]	458	49.98	A	1.00	87.24	1	1	1	3
Tc00.1047053509053.70	p22 protein precursor, putative [7980.t00007]	226	25.81	F	1.00	87.11	3	1	1	5
Tc00.1047053503815.10	alkyl-dihydroxyacetone phosphate synthase, putative [4766.t00001]	614	69.25	B	1.00	86.88	1	1	1	3
Tc00.1047053445777.10	retrotransposon hot spot (RHS) protein, putative [2627.t00001]	260	29.51	C	0.98	86.82	5	2	2	11
Tc00.1047053511151.90	glycerol-3-phosphate dehydrogenase, putative [8609.t00009]	604	67.07	A,C	1.00	86.6	2	1	1	3
Tc00.1047053509153.120	acyl-CoA dehydrogenase, putative [8016.t00012]	402	43.98	A,C,E	1.00	85.24	3	2	2	11
Tc00.1047053506219.40	hypothetical protein, conserved [5346.t00004]	311	34.11	A	1.00	85.1	7	2	2	8
Tc00.1047053504103.20	hypothetical protein, conserved [4910.t00002]	1006	109.72	A	1.00	80.05	1	1	1	2
Tc00.1047053509317.80	hypothetical protein, conserved [8069.t00008]	144	16.17	A,C,E	1.00	79.95	3	1	1	10
Tc00.1047053506893.100	hypothetical protein, conserved [7130.t00010]	262	29.39	F	1.00	79.81	1	1	1	5
Tc00.1047053506583.60	mitochondrial elongation factor G, putative [7008.t00006]	747	84.53	C	1.00	77.67	2	1	1	2
Tc00.1047053511071.130	basal body component, putative [8589.t00013]	1423	165.55	B	1.00	74.47	1	1	1	1
Tc00.1047053503571.19	hypothetical protein, conserved [4644.t00002]	709	79.18	A	1.00	74.27	1	1	1	2
Tc00.1047053504125.50	mitochondrial carrier protein, putative [4921.t00005]	294	31.50	A	1.00	74.08	6	1	1	5
Tc00.1047053506519.130	inosine-5'-monophosphate dehydrogenase, putative [6982.t00013]	492	52.26	A,C,E	1.00	73.59	3	1	1	3

Tc00.1047053509733.170	transitional endoplasmic reticulum ATPase, putative [8185.t00017]	779	86.04	A	1.00	73.49	1	1	1	2
Tc00.1047053509109.30	hypothetical protein, conserved [8002.t00003]	827	94.58	A-C	1.00	72.85	4	1	1	2
Tc00.1047053503959.10	hypothetical protein, conserved [4838.t00001]	569	63.56	A	1.00	72.82	1	1	1	3
Tc00.1047053503887.40	hypothetical protein, conserved [4802.t00004]	121	13.41	G	1.00	72.38	1	1	1	12
Tc00.1047053508275.9	dynein heavy chain, putative [7666.t00001]	1750	199.46	A	1.00	71.88	1	1	1	1
Tc00.1047053511635.10	histone H2B, putative [8741.t00001]	113	12.33	B,F-G	1.00	70.93	4	1	1	12
Tc00.1047053506445.110	nucleobase transporter, putative [6947.t00011]	441	47.51	A	1.00	70.77	1	1	1	3
Tc00.1047053507053.180	hypothetical protein, conserved [7189.t00018]	810	88.04	A	1.00	69.89	1	1	1	2
Tc00.1047053433273.10	dynein heavy chain, putative [2037.t00001]	423	48.39	A	1.00	69.28	1	1	1	3
Tc00.1047053504153.250	hypothetical protein, conserved [4935.t00025]	393	42.87	A-B	1.00	68.65	2	1	1	3
Tc00.1047053508153.1100	MP99, putative [7617.t00110]	915	100.27	C	1.00	66.04	4	1	1	2
Tc00.1047053503843.40	chaperone DnaJ protein, putative [4780.t00004]	635	71.56	A	1.00	65.28	1	1	1	2
Tc00.1047053506679.100	40S ribosomal protein S18, putative [7052.t00010]	154	17.52	B	1.00	64.04	1	1	1	8
Tc00.1047053510089.210	hypothetical protein, conserved [8300.t00021]	145	17.04	F	1.00	61.94	1	1	1	7
Tc00.1047053511029.20	kinetoplast DNA-associated protein, putative [6032.t00002]	208	23.72	A-B	1.00	61.54	2	1	1	7
Tc00.1047053508719.40	kinetoplast DNA-associated protein, putative [5609.t00004]	129	14.63	F	1.00	61.52	1	1	1	11
Tc00.1047053504147.120	60S ribosomal protein L22, putative [4932.t00012]	131	15.25	F	1.00	61.22	1	1	1	8
Tc00.1047053507047.150	hypothetical protein, conserved [5430.t00015]	506	56.77	B	1.00	61	1	1	1	3
Tc00.1047053506503.140	extracellular receptor, putative [6974.t00014]	889	97.75	A	1.00	60.27	1	1	1	1
Tc00.1047053507777.20	hypothetical protein, conserved [5516.t00002]	102	11.74	G	1.00	60.06	8	1	1	12
Tc00.1047053508153.270	heat shock protein 20, putative [7617.t00027]	143	15.81	F	1.00	59.76	3	1	1	8
Tc00.1047053504037.30	60S ribosomal protein L12, putative [4877.t00003]	165	17.69	F	1.00	59.44	1	1	1	10
Tc00.1047053507713.30	heat shock protein 85, putative [7443.t00003]	705	80.66	C	1.00	59.22	1	1	1	2
Tc00.1047053509793.50	hypothetical protein, conserved [5785.t00005]	193	21.27	A	1.00	57.89	1	1	1	6
Tc00.1047053503903.60	hypothetical protein, conserved [4810.t00006]	105	12.63	G	1.00	57.8	1	1	1	10
Tc00.1047053510119.20	elongation factor 1-alpha (EF-1-alpha), putative [8309.t00002]	450	49.07	E	1.00	57.52	16	1	1	3
Tc00.1047053509561.20	flagellum-adhesion glycoprotein, putative [8133.t00002]	580	62.21	B	1.00	57.27	1	1	1	2
Tc00.1047053510529.30	hypothetical protein, conserved [8419.t00003]	1153	128.75	B,G	1.00	56.99	3	1	1	1
Tc00.1047053506933.60	mitochondrial RNA binding protein, putative [7145.t00006]	143	14.89	G	1.00	56.85	3	1	1	9
Tc00.1047053407477.50	cytochrome c oxidase VIII (COX VIII), putative [10960.t00005]	158	18.57	G	1.00	56.81	8	1	1	7
Tc00.1047053508413.68	kinetoplastid membrane protein KMP-11 [7719.t00008]	92	10.86	A-B,G	1.00	56.78	3	1	1	11
Tc00.1047053506295.70	hypothetical protein, conserved [6889.t00007]	121	14.88	A-B,F-G	1.00	56.47	4	1	1	9
Tc00.1047053504427.64	hypothetical protein, conserved [6288.t00029]	89	10.35	G	0.99	56.03	3	1	1	13
Tc00.1047053511529.160	enoyl-CoA hydratase/isomerase family protein, putative [6142.t00016]	363	40.55	A	1.00	55.47	1	1	1	3
Tc00.1047053503999.30	hypothetical protein, conserved [4858.t00003]	1381	151.60	B	1.00	55.27	3	1	1	1
Tc00.1047053506963.14	40S ribosomal protein S27, putative [7154.t00016]	87	9.54	B	1.00	55.17	1	1	1	16
Tc00.1047053508719.60	kinetoplast DNA-associated protein, putative [5609.t00006]	128	14.33	F	1.00	54.87	4	1	1	10
Tc00.1047053506195.110	malate dehydrogenase, putative [6847.t00011]	319	33.31	C	1.00	54.75	1	1	1	4
Tc00.1047053508153.130	enoyl-CoA hydratase, mitochondrial precursor, putative [7617.t00013]	268	28.75	B,E	1.00	54.61	1	1	1	5
Tc00.1047053508699.130	cation transporter, putative [7835.t00013]	198	20.42	E	1.00	54.51	2	1	1	6
Tc00.1047053503449.14	hypothetical protein, conserved [4583.t00005]	181	20.30	F	0.98	52.99	1	1	1	6
Tc00.1047053510155.70	heat shock protein 70 (HSP70), putative [8320.t00007]	884	96.90	B	1.00	52.35	1	1	1	1
Tc00.1047053504153.160	carboxypeptidase, putative [4935.t00016]	504	57.61	B	1.00	52.22	1	1	1	3
Tc00.1047053507715.34	hypothetical protein, conserved [7444.t00011]	144	16.46	F	1.00	51.86	1	1	1	7
Tc00.1047053506579.10	ABC transporter, putative [7006.t00001]	701	78.13	A,C	1.00	51.65	2	1	1	2
Tc00.1047053508719.30	hypothetical protein, conserved [5609.t00003]	193	21.24	E	0.99	49.98	3	1	1	6
Tc00.1047053507711.300	hypothetical protein, conserved [7442.t00030]	106	11.94	D	1.00	49.69	1	1	1	11
Tc00.1047053508547.160	hypothetical protein, conserved [7773.t00016]	793	90.48	C	0.99	49.68	1	1	1	2
Tc00.1047053511071.190	hypothetical protein, conserved [8589.t00019]	172	19.27	A	0.99	48.61	1	1	1	8
Tc00.1047053504163.60	hypothetical protein, conserved [4940.t00006]	190	22.42	E	0.99	48.39	1	1	1	6
Tc00.1047053504069.50	peroxin 14, putative [4893.t00005]	370	39.99	F	0.99	48.26	1	1	1	4
Tc00.1047053506163.50	hypothetical protein, conserved [6836.t00005]	397	42.63	F	0.99	47.89	1	1	1	3
Tc00.1047053455721.9	cytochrome c oxidase subunit 10, putative [3181.t00003]	116	13.82	G	0.99	46.55	2	1	1	10
Tc00.1047053506779.120	hypothetical protein, conserved [7094.t00012]	211	23.34	A-B	0.99	46.33	4	1	1	5
Tc00.1047053506789.240	hypothetical protein, conserved [7098.t00024]	241	25.73	F	0.99	46.15	2	1	1	5
Tc00.1047053506789.140	hypothetical protein, conserved [7098.t00014]	772	87.03	B	0.99	46.11	1	1	1	2
Tc00.1047053506949.50	cytochrome c, putative [5417.t00005]	115	12.21	G	0.99	45.81	1	1	1	11
Tc00.1047053506983.39	calpain-like cysteine peptidase, putative [5423.t00007]	115	12.82	G	0.99	45.65	1	1	1	10
Tc00.1047053506755.260	mitochondrial RNA-binding protein 2, putative [7083.t00026]	227	25.48	F	0.99	45.47	2	1	1	5
Tc00.1047053504427.60	hypothetical protein, conserved [6288.t00006]	315	33.67	A	1.00	45.23	1	1	1	6
Tc00.1047053508999.260	calpain-like cysteine peptidase (pseudogene), putative [7958.t00026]	131	14.62	G	0.99	45.16	1	1	1	8
Tc00.1047053506753.240	hypothetical protein, conserved [7082.t00024]	193	21.70	F	0.97	44.1	1	1	1	5
Tc00.1047053509611.170	hypothetical protein, conserved [8149.t00017]	633	71.97	A	0.99	43.98	1	1	1	2
Tc00.1047053504035.84	hypothetical protein, conserved [4876.t00015]	89	10.31	G	0.98	43.39	2	1	1	11
Tc00.1047053506755.250	mitochondrial import inner membrane translocase subunit Tim17, putative [7083.t00025]	153	16.12	F	0.95	43.37	2	1	1	7
Tc00.1047053509045.20	co-chaperone GrpE, putative [7978.t00002]	220	24.33	E	0.97	43.12	1	1	1	5
Tc00.1047053506629.40	hypothetical protein, conserved [7029.t00004]	523	57.65	C	0.97	42.97	1	1	1	3
Tc00.1047053504013.100	ribosomal protein S19, putative [4865.t00010]	168	18.76	F	0.97	42.78	1	1	1	7
Tc00.1047053506475.116	glutaredoxin, putative [6960.t00015]	108	12.30	G	0.98	42.64	1	1	1	10
Tc00.1047053399373.9	retrotransposon hot spot (RHS) protein, putative [9155.t00003]	658	75.12	A	0.97	41.77	1	1	1	2
Tc00.1047053505169.10	polyubiquitin (pseudogene), putative [6526.t00001]	324	36.16	B	0.97	41.65	8	1	1	3
Tc00.1047053509537.50	hypothetical protein, conserved [8127.t00005]	2518	283.76	A	0.97	41.54	4	1	1	0
Tc00.1047053511229.30	hypothetical protein, conserved [6080.t00003]	301	33.53	A	0.99	41.22	1	1	1	5
Tc00.1047053408437.20	hypothetical protein, conserved [653.t00002]	121	13.40	G	0.96	40.95	2	1	1	8
