# Supplementary material for: Identification of Contractile Vacuole Proteins in Trypanosoma cruzi
Source: PLoS One. 2011 Mar 18;6(3):e18013. doi: 10.1371/journal.pone.0018013 (PMC3060929; doi:10.1371/journal.pone.0018013)
Supplement: Table S2 — Peptide list for all proteins matched to mass spectra from the contractile vacuole data set. This Table includes all proteins with above 1% false discovery rate and a total protein probability > 0.95. (PDF) [file pone.0018013.s002.pdf]

Table S2. Peptide list for all proteins matched to mass spectra from the *T. cruzi* contractile vacuole data set. Included here are all proteins with above a 1% false discovery rate and a total protein probability > 0.95. Data for each protein were collated from TcruziDB.org and GenBank. If annotated descriptions of gene products differ from the specific name used in the text, the annotated protein name is given in parenthesis.

Tc#	Protein Accession	Name	SCORE	Spectra	# of Peptides	% Coverage	Peptide	Peptide Score
Tc00.1047053510101.140	EAN99374.1	pyruvate phosphate dikinase, putative	2332	384	44	49	CCVSGCGDLVIK +3 Carbamidomethyl (C)	69
							ELLGGK	38
							ELTEEYLVLFQR	43
							FDTDLTAK	48
							FVYDSYR	48
							FVYDSYRR	28
							GDFETILEWCR +Carbamidomethyl (C)	52
							GDFLGIFR	28
							GILTAR	15
							GLAASPGAAVGQIVFDADSAK	130
							GLIYAGQLK	36
							DDAGSFLR	49
							GLPQEVVEQVK	73
							GLPVTIR	21
							GTKFDTDLTAK	53
							HYCSLGIYSK +Carbamidomethyl (C)	55
							IAVQK	18
							IDAIR	17
							IDPLQVDHLMHPNIEPGAAK	23
							KFVYYFGGQK	54
							LETSPEDLAGMDAAR	97
							LLCEIR +Carbamidomethyl (C)	20
							DLKELTEEYLVLFQR	85
							LLDPPLHEFVPHEDAAQAELAK	55
							LQSPDLK	30
							MGICGEHGGDPTTIGFCHK +2 Carbamidomethyl (C)	28
							QFTLSGR	29
							SATGVAFSR	34
							SFGAEGVGLCR +Carbamidomethyl (C)	75
							SPSTGENFFFGEYLVNAQGEDVVAGIR	129
							TAEETLAAAGQR	87
							TEHMFFEGSR	34
							TKGLPQEVVEQVK	55
							DPFESIDQEGVGELMR	97
							TPQQIGQSLSLR	88
							VDYIVGTMIEVPR	55
							VGLNYVSCSPFR +Carbamidomethyl (C)	79
							VIPEIMIPLVGK	81
							VPVATVAAAHAALK	109
							EDFEEAIGHMK	29
							EELTFTK	26
							EGDYITLDGSK	66
							EGLITK	30
							EGLITKEEAVLR	16
Tc00.1047053506297.190	EAN98352.1	pyruvate phosphate dikinase, putative	2245	382	43	48	CCVSGCGDLVIK +3 Carbamidomethyl (C)	69
							ELLGGK	38

							ELTEEYLVLFQR	43
							FDTDLTAK	48
							FVYDSYR	48
							FVYDSYRR	28
							GDFETILEWCR +Carbamidomethyl (C)	52
							GDFLGIFR	28
							GILTAR	15
							GLAASPGAAVGQIVFDADSAK	130
							GLIYAGQLK	36
							DDAGSFLR	49
							GLPQEVVEQVK	73
							GLPVTIR	21
							GTKFDTDLTAK	53
							HYCSLGIYSK +Carbamidomethyl (C)	55
							IAVQK	18
							IDAIR	17
							IDPLQVDHLMHPNIEPGAAK	23
							KFVYYFGGQK	54
							LETSPEDLAGMDAAR	97
							LLCEIR +Carbamidomethyl (C)	20
							DLKELTEEYLVLFQR	85
							LLDPPLHEFVPHEDAAQAELAK	55
							LQSPDLK	30
							MGICGEHGGDPTTIGFCHK +2 Carbamidomethyl (C)	28
							QFTLSGR	29
							SATGVAFSR	34
							SFGAEGVGLCR +Carbamidomethyl (C)	75
							SPSTGENFFFGEYLVNAQGEDVVAGIR	129
							TAEETLAAAGQR	87
							TEHMFFEGSR	34
							TKGLPQEVVEQVK	55
							DPFESIDQEGVGELVR	91
							TPQQIGQSLSLR	88
							VDYIVGTMIEVPR	55
							VGLNYVSCSPFR +Carbamidomethyl (C)	79
							VPVATVAAAHAALK	109
							EDFEEAIGHMK	29
							EELTFTK	26
							EGDYITLDGSK	66
							EGLITK	30
							EGLITKEEAVLR	16
Tc00.1047053507029.30	EAN84370.1	heat shock 70 kDa protein, mitochondrial precursor, putative	1408	80	21	36	DAAVDLK	46
							QFFGR	22
							QLTEWK	19
							QYSPSQVGAFVLEK	93
							SQTFSTAADNQTQVGIK	98
							STGIDLSNER	58
							TLLAELR	23
							TQNITITASGGLSK	94
							TTPSVVAFK	16
							VIEAVK	42
							VLENTEGFR	66
							DAGTIAGLNVIR	102
							VSNAVVTCPAYFNDAQR +Carbamidomethyl (C)	92
							VVNEPTAAALAYGLDK	108

							ELVEVR	23
							ETAENFLGR	46
							FEDSNIQHDIK	52
							GLVLLDVTPLSLGIETLGGVFTR	97
							GVNPDEAVALGAATLGGVLR	126
							KVSNAVVTCPAYFNDAQR +Carbamidomethyl (C)	99
							QAITNPQSTFFAVK	86
Tc00.1047053507641.290	EAN97657.1	chaperonin HSP60, mitochondrial precursor	1186	61	17	42	AAVQEGIVPGGGVALLR	95
							KVTSTENIVQVATISANGDEELGR	60
							LPAHTIVLNAGK	71
							LVGEEGSGLELDAENFDPAILGTVK	112
							NVIIEQSYGAPK	79
							TGVQIIR	30
							TNDLAGDGTTTSAVLVASVFSESLR	157
							VELLR	19
							VLENNDVTVGYDAQR	91
							AELEDAFVLVSAK	34
							AIEFK	19
							ALDSLLGDSSLTADQR	101
							AVGVILQSVAEQSR	81
							AVSAVATTLGPK	88
							DGVITTQDGK	19
							GLIDGETSDYNR	67
							GYISPYFVTDAK	63
Tc00.1047053506839.30	EAN90344.1	hypothetical protein, conserved	857	24	15	17	AAFFISPILR	47
							ITFFDLSETSVTSR	105
							ITPTVEEVEIR	53
							IVLDK	25
							LDEVDDDTQFK	48
							NTHIVSLDLSHNR	22
							VIAFPNNNITSR	59
							DALYLYEDPSK	51
							DLVYR	19
							DNTNLLELNLGNCGFGDK +Carbamidomethyl (C)	65
							DSQVPFTDSACQLLCASLVK +2 Carbamidomethyl (C)	88
							GVELLAEALR	85
							IDANENIPEVVLK	65
							IGNFSAQNLTKTLFEK	24
							INDEAALSLLNTIR	101
Tc00.1047053506585.40	EAN87966.1	glucose-regulated protein 78, putative	779	52	12	25	DAGTIAGLNVVR	88
							NSLESVAYSLR	83
							VEVDSLTEGFDFSEK	100
							VQQLIR	29
							EIAETYLGEK	31
							FELSGIPPAPR	35
							GINPDEAVAYGAAVQAAVLTGESEVGGR	127
							IINEPTAAAIAYGLNK	103
							ITPSVVAFTETER	94
							LLSYEVVADKDGKPK	16
							LQSVTNPIIQK	58
							NQLPQNPHNTIYAIK	15
Tc00.1047053511215.119	EAN92318.1	69 kDa paraflagellar rod protein, putative	647	27	10	25	AQELLSSVEGGTK	99
							VCGLQLSVR +Carbamidomethyl (C)	65
							AQLAAIEK	35
							AQLLEHLVELVADK	98

							AQVEEELEMLKDK	30
							EASGAVGPADQQQPAVPEVTDVTLEAAR	55
							IIGQTEDENKPFGR	66
							KVEYQQFLDVCGQHK +Carbamidomethyl (C)	57
							TSCLSNEEFIQDLHVSDWSETQK +Carbamidomethyl (C)	49
							TVSFTGTIDNAIAK	93
Tc00.1047053506247.220	EAN98485.1	histidine ammonia-lyase, putative	645	31	10	27	ALDMLAIGVHELGNISER	79
							VILDGCSLTPDVLYALGYEK +Carbamidomethyl (C)	85
							ATNSTLNPDIHR	64
							EGLALINGTQFISALGAEAVVR	100
							ELGVEPITLAAK	41
							FESTIIPPHQLEELQLNLIR	58
							GATIEISDEAVAR	79
							QTVYGINTGFGK	91
							SGAVWK	22
							SVSPPWEEDR	26
Tc00.1047053510215.10	EAN81600.1	NADH-dependent fumarate reductase, putative	628	42	10	11	AKEVADEVLR	35
							VVAEAVAFSR	82
							FVNELDLR	49
							GVFDPATGPIIEALR	66
							LGGNSLLECVVFGR +Carbamidomethyl (C)	65
							LYTSIFDWK	62
							NTLGDYEQLSK	71
							RPIFGLFGAGEVTGGVHGGNR	82
							SGLGGSTDPGLVR	50
							SGLQLGQFIAIR	66
Tc00.1047053508741.229	EAN99221.1	hypothetical protein, conserved	624	42	11	48	ATLEPSTCNVNLTASYLFQGHK +Carbamidomethyl (C)	48
							YAYAGYALAVR	65
							YAYAGYALAVRTNKLR	20
							GQGWTGTLGFETACVLFK +Carbamidomethyl (C)	100
							LFVNPMATSDGK	62
							LVDNWTAAVTLDK	85
							NKEWAVVYIAK	37
							NLKPGVLLTHS	39
							NYDLTLGCR +Carbamidomethyl (C)	59
							SLTLYK	25
							SYTTSFATPIPK	84
Tc00.1047053505763.19	EAN82405.1	P-type H+-ATPase, putative	559	18	8	15	ADVGIAVQGATDAAR	93
							DGAWQQLDAALLVPGDLVK	97
							FSVTK	19
							GEVDGTVQYTGQNTFFGK	92
							GLTTAEAEELLAK	73
							SVLVLAALAAK	90
							TNEKFSVTK	20
							VDVNDLPDDLGEK	75
Tc00.1047053508737.210	EAN95832.1	hypothetical protein, conserved	556	36	10	43	ATLEPSTCNVNLTASYLCQGHK +2 Carbamidomethyl (C)	64
							YAYAGYALAVRTNKLR	20
							GQGWTGTLGFETACVLFK +Carbamidomethyl (C)	100
							LFVNPMATSDGK	62
							LVDNWTAAVTLDK	85
							NKEWAVVYIAK	37
							NLKPGVLLTHS	39
							NYDLTLGCR +Carbamidomethyl (C)	59
							SLTLYK	25
							YAYAGYALAVR	65

Tc00.1047053509445.39	EAN82723.1	glutamate dehydrogenase, putative	536	43	11	28	ALLLK	25
							WQPFVNPQPK	54
							YQELLTR	43
							AVQLFTGGLSDSSYGLCYTEDEVTNHVK +Carbamidomethyl (C)	103
							FKGSVGPVFTLVDGK	50
							GFLCAK +Carbamidomethyl (C)	19
							GSVGPVFTLVDGK	63
							GYTTDDGTTSVYTAK	76
							LGETDIK	24
							LNSLR	24
							QLASHVFLEQR	55
Tc00.1047053506943.50	EAN90286.1	glyceraldehyde 3-phosphate dehydrogenase, putative	518	19	9	36	AAAVNIIPSTTGAAK	80
							AVGMVIPSTQGK	41
							GILGYTDEELVSADFINDNR	98
							IVSWYDNEWGYSHR	21
							KVVISAPASGGAK	54
							LGVEYVIESTGLFTAK	87
							TVDGVSVK	20
							VPTPDVSVVDLTFTAAR	92
							VVDLVR	45
Tc00.1047053505843.10	EAN84271.1	glutamate dehydrogenase, putative	508	44	11	46	ANILASDVFK	59
							YILFHYTPK	55
							YVKDIIDR	39
							DIIDR	28
							ENTPEFYK	36
							ENTPEFYKK	26
							GGVTSSSLEVYSGLALLDEEHEK	51
							IIVEGANLFISQDAR	96
							LALER	21
							REFEAIWR	32
							TLISDTLSEK	65
Tc00.1047053511903.40	EAN96072.1	hypothetical protein, conserved	493	27	9	23	ADKDFSSLLSR	36
							HACLGGVYALK +Carbamidomethyl (C)	52
							IGFLGLGTESSTDNSAGSIIVR	123
							INVAAALEKPVNLTR	57
							LILNYDVDPAR	59
							VALKDWCDWTGNDWK +Carbamidomethyl (C)	45
							VPQLVELDTK	52
							VSNVVGESFR	45
							YFEELCSK +Carbamidomethyl (C)	24
Tc00.1047053503849.60	EAN88381.1	NADH-dependent fumarate reductase, putative	482	21	7	12	FAFCVLNDAAVR +Carbamidomethyl (C)	61
							FVNELDLR	49
							LGGNSLLECVVFGR +Carbamidomethyl (C)	65
							NTLGDYEQLSK	71
							RPIFGLFGAGEVTGGVHGGNR	82
							SGLGGSTDPGLVR	50
							VYGVEVLQDEGVASR	104
Tc00.1047053411235.9	EAN81053.1	alpha tubulin, putative	482	42	6	16	AVFLDLEPTVVDEIR	97
							DVNAAVATIK	60
							FDGALNVDLTEFQTNLVPYPR	124
							LIGQVVSALTASLR	113
							RTIQFVDWSPTGFK	17
							TIQFVDWSPTGFK	71
Tc00.1047053509237.130	EAN92788.1	cytoskeleton-associated protein CAP5.5, putative	479	13	7	13	FEPSEGPYFVLPR	79
							GYGGVNELLGK	39

							SDVFAQALSEEYR	78
							TVLLDDFLPTVNEVPCFAR +Carbamidomethyl (C)	62
							VLDDPSELWVSLLQK	74
							VLFVVEDAAEVIRPLQK	49
							YLEAGNIVLLNTPAGK	98
Tc00.1047053507547.90	EAN88964.1	glycosomal phosphoenolpyruvate carboxykinase, putative	468	20	8	19	AIGLNPK	21
							FGAVAENCVLDKR +Carbamidomethyl (C)	46
							HATFYGEQLAEK	80
							IVDTDDVRENVDWGK	21
							NLIGDDEHVWTDR	55
							NLLSPELVQWALK	84
							TGEIDFYDESICK +Carbamidomethyl (C)	76
							VWLLNTGYAGGR	85
Tc00.1047053510943.50	EAN95227.1	delta-1-pyrroline-5-carboxylate dehydrogenase, putative	465	14	7	16	ASGSNDKPGSPLFLTR	53
							DLAGVAFTGSTK	93
							ESVTDCPIVIGGK +Carbamidomethyl (C)	48
							GAFEFQGQK	50
							SSPSTYSVIAGGGYDK	91
							YAAGNYYINDK	54
							YALTGSIFAQDR	76
Tc00.1047053509129.10	EAN81235.1	hypothetical protein, conserved	455	30	8	36	DYDLTLGCR : Carbamidomethyl (C8)	49
							GQGWTGTLGFETACVLFK : Carbamidomethyl (C14)	100
							LFVNPMATSDGK	62
							LVDNWTAAVTLDK	85
							NKEWAVVYIAK	37
							NLKPGVLLTHS	39
							SLTLYK	25
							SYTTSFVTPIPK	48
Tc00.1047053504087.20	EAN86342.1	hypothetical protein	450	15	8	4	ASTTAGVLFLPFR	66
							ELADACPDVGVLLVESWK +Carbamidomethyl (C)	40
							LSPLGLCEAEEASAR +Carbamidomethyl (C)	90
							RPTFVVYR	47
							SAGLALHPLLLYSESVLIPQR	45
							SITGPVLGADR	28
							SYGFPTSSLIVSSK	49
							VFQSSIAIPWSAGK	85
Tc00.1047053508535.10	EAN85469.1	NADH-dependent fumarate reductase, putative	448	15	8	9	GLEHTVPYTLR	30
							LGGNSLLECVVFGR +Carbamidomethyl (C)	65
							SCGGILIER +Carbamidomethyl (C)	43
							SDAESTLTEEEVER	23
							SGLGGSTVPSLVR	48
							SGLYLGQFIAIR	49
							SLLEPVESELAQVSIK	91
							SVVSNAIIEQGDEYPYSGGSK	99
Tc00.1047053511277.290	EAO00045.1	aconitase, putative	441	10	7	11	IHPLTAANYLASPPLVVAYALSGR	68
							LVGEGQTGPFTIYFPTNEK	95
							NCDEFDITSK +Carbamidomethyl (C)	44
							VDIDFNEEPIAK	66
							VVIAESFER	66
							YETLPFSIR	20
							YQQENIPLVIIAGK	82
Tc00.1047053506211.160	EAN97607.1	ADP,ATP carrier protein 1, mitochondrial precursor, putative	435	21	8	30	GAGANILR	18
							GIAGAGVLSGVDALKPIYVR	98
							GNLSNVLR	16
							LGFFEEFMVGGVAAGVSK	112

							LLVQNQGEMIK	47
							TEGLYSLWR	49
							TVAAPIER	18
							YFPTQALNFAFK	77
Tc00.1047053509683.10	EAN93482.1	hypothetical protein	432	14	8	4	ASSTAGVLFLPFR	48
							ELADACPDVGVLLVESWK +Carbamidomethyl (C)	40
							LSPLGLCEAEEASAR +Carbamidomethyl (C)	90
							RPTFVVYR	47
							SAGLALHPLLLYSESVLIPQR	45
							SITGPVLGADR	28
							SYGFPTSSLIVSSK	49
							VFQSSIAIPWSAGK	85
Tc00.1047053508555.60	EAN87963.1	cytoskeleton-associated protein CAP5.5, putative	418	12	6	10	ATVPEEVLER	50
							FEPSEGPYFVLPR	79
							GYGGVNELLGK	39
							SDVFAQALSEEYR	78
							VLDDPSELWVSLLQK	74
							YLEAGNIVLLNTPAGK	98
Tc00.1047053509617.20	EAN87979.1	paraflagellar rod protein 3, putative	418	25	6	12	CTGLVEELVSEGCAAVK +2 Carbamidomethyl (C)	79
							SQLDATQLAQVPTR	96
							TQLAQLEK	39
							VLQDLK	25
							VVSFTQMIDNAIAK	107
							WNLTEAYDLAK	72
Tc00.1047053510149.80	EAN92058.1	ABC transporter, putative	415	11	9	7	DALIDHLSPGEHLR	28
							DGALCVRDR	15
							LLDALGLTSQK	36
							LYAGLR	20
							SAQASLSHYGDLLVAGK	56
							TSSSTEGEECVLEGVVGR +Carbamidomethyl (C)	97
							TTAVGILAGEVVPTSGSAYINHLSVLR	44
							VFPTVAEAEK	33
							YIALALTSAQGK	86
Tc00.1047053506337.70	EAN93176.1	2-oxoglutarate dehydrogenase E1 component, putative	397	6	6	9	EQLQPVLDALK	17
							FGADGAESLVVGLR	46
							FLQAVAEDVDTPAYSPAER	122
							KPLILFFSK	51
							LDLASFGFTPDDYNR	78
							YSGELIASGVITPQQQTAK	83
Tc00.1047053509351.10	EAN81376.1	delta-1-pyrroline-5-carboxylate dehydrogenase, putative	396	9	6	29	ASGSNDKPGSPLFLTR	53
							GAFEFQGQK	50
							SSPSTYSVIAGGGCYK +Carbamidomethyl (C)	87
							TEGWFVQPTIIESK	76
							YAAGNYYINDK	54
							YALTGSIFAQDR	76
Tc00.1047053506491.20	EAN86336.1	myosin heavy chain, putative	391	20	8	2	ALEGENAALAALLAAK	82
							EADNEKLAEELAQR	57
							EAVIEGAEADASK	41
							GSLQEVQGALR	71
							LAEDLAQR	43
							LAEELAQR	48
							LTEELAQR	29
							NDEINRLK	20
Tc00.1047053504153.310	EAN99073.1	heat shock protein, putative	387	18	6	10	ALLYVPQSHTEK	66
							EELAENLGTIAGSGSK	96

							FIQEYGPFLK	59
							GAVDSESIPLNVSR	72
							SDIDYPLVSLEEYR	68
							YNFHFNPK	26
Tc00.1047053511909.40	EAN87573.1	succinate dehydrogenase flavoprotein, putative	386	8	6	13	AFGGQSIHFGGK	52
							AITLEVLAGR	21
							LGANSLLDIVVFGK	93
							NLLTNALLTITGAAVR	96
							SKYTILATGGYGR	21
							SPVWNSNLIEALELR	103
Tc00.1047053507089.270	EAN96941.1	dihydrolipoyl dehydrogenase, putative	385	13	7	21	CAPTLDEDVTNALVGALAK +Carbamidomethyl (C)	53
							GEGSFETAHSIR	65
							GLTGGVEYLFK	43
							GTLGGTCLNVGCIPSK +2 Carbamidomethyl (C)	68
							RPFTGGLGLDK	51
							TIIATGSEPTELPFLPFDEK	77
							VVLSSTGALALPR	28
Tc00.1047053511289.70	EAN90413.1	ADP,ATP carrier protein 1, mitochondrial precursor, putative	362	19	8	29	GAGANILR	18
							GNLSNVLR	16
							LDRPYTGVTDCFVHTMK +Carbamidomethyl (C)	25
							LGFFEEFMVGGVAAGVSK	112
							LLVQNQGEMIK	47
							TEGLYSLWR	49
							TVAAPIER	18
							YFPTQALNFAFK	77
Tc00.1047053509551.30	EAN92689.1	mitochondrial phosphate transporter, putative	361	13	7	23	DLYANLAGEQAAK	32
							GIFSGFK	34
							SGFPFK	17
							STLGLGTSGGTGK	73
							STLGLGTSGGTGKK	29
							VQTSPSGTFPTAFGAALAAMR	135
							VVLSEDGCGSK +Carbamidomethyl (C)	41
Tc00.1047053509999.90	EAN93060.1	carnitine/choline acetyltransferase, putative	359	12	5	13	APLPVNTNPAILIK	55
							LDTFLAGSAVTLQNALVEANK	125
							NAGETQPSVAASIVYGIAK	84
							TSILPLKPADTVK	27
							WVGDVLQSIESK	68
Tc00.1047053510837.20	EAN82710.1	glutamamyl carboxypeptidase, putative	349	31	6	21	AEFEDAEIVITPR	48
							ANLWATLPGDGGVTK	64
							DETPSFEGSEEAPITK	81
							GGIILSGHTDVVPVDGQK	33
							LVAFDTTSR	51
							NSNLELIHYCK +Carbamidomethyl (C)	72
Tc00.1047053507689.30	EAN85497.1	glutamamyl carboxypeptidase, putative	348	31	6	21	AEFEDAEIVITPR	48
							ANLWATLPGDGGVTK	64
							DETPSFEGSEEAPFTK	80
							GGIILSGHTDVVPVDGQK	33
							LVAFDTTSR	51
							NSNLELIHYCK +Carbamidomethyl (C)	72
Tc00.1047053508153.340	EAO00202.1	6-phospho-1-fructokinase, putative	343	6	5	12	EITVGLQDDVR	48
							ELEAISLVR	73
							SHAAPLNEVTQEDLK	80
							TIDNDLSFSHR	66
							YGGTILGSSR	76
Tc00.1047053509499.14	EAN84888.1	tryparedoxin peroxidase, putative	342	10	6	26	DYGVLIEEQGISLR	80

							GLFIIDDK	46
							HITVNDLPVGR	56
							IQDISLNDYK	68
							TATVR	15
							VVQAFQYVDK	77
Tc00.1047053503899.119	EAN90957.1	trypanothione/tryparedoxin dependent peroxidase 2, putative	323	14	6	31	ATIPGLFGTK	46
							FSPGASVEDIEK	52
							FSPGASVEDIEKK	66
							GGYETATTLYNK	67
							GHPLLIYNVASR	63
							KLLPLLGGAR	29
Tc00.1047053509961.70	EAN90957.1	dispersed gene family protein 1 (DGF-1, pseudogene), putative	323	10	8	3	ASLYVVGWR	19
							DGDCFAPLTTAVSDCK +2 Carbamidomethyl (C)	68
							ESPSDAFAYAYPR	46
							EVSYDGVFPEK	28
							HCVLVGDVQLR +Carbamidomethyl (C)	34
							SAGIIGK	23
							VVASEGAVLR	37
							YGPALVLDGVR	68
Tc00.1047053509585.10	EAN84848.1	dynein heavy chain, putative	321	6	5	2	AKPLVEEALAALDTLDK	92
							DGILSMIFR	36
							EAGVQVAALQEVLQR	77
							LGFTYDNER	27
							YDALLFEVIGR	89
Tc00.1047053508209.120	EAN91214.1	10 kDa heat shock protein, putative	317	11	5	53	AGVLIPEQVAGK	58
							DWTPTVK	23
							TLAAK	34
							VNDTVLLPEFGGSSVK	88
							VNEGTVVAVAAATK	114
Tc00.1047053510679.40	EAN85276.1	hypothetical protein	299	30	4	13	FADANEENVPAAQVNR	82
							HIDASLLSR	32
							IEVPLAEPPLYLWCR +Carbamidomethyl (C)	95
							SNSEEYPDILFVPTLER	90
Tc00.1047053508209.100	EAN91212.1	10 kDa heat shock protein, putative	292	14	4	50	AGVLIPEQVAGK	58
							DWTPTVK	23
							VDDTVLLPEFGGSSVK	96
							VNEGTVVAVAAATK	114
Tc00.1047053507187.9	EAN82246.1	dispersed gene family protein 1 (DGF-1), putative	271	6	5	4	DGDCFAPLTTAVSDCK +2 Carbamidomethyl (C)	68
							ESPSDAFAYAYPR	46
							EVSYDGVFPEK	28
							GASCLPFGVPDTVVPPLPER +Carbamidomethyl (C)	61
							YGPALVLDGVR	68
Tc00.1047053508827.40	EAN92888.1	acyl-CoA dehydrogenase, putative	268	5	3	6	ELFESLLEEASK	80
							ISTLYEGTTGIQSLDFIGR	121
							SDVNKELFESLLEEASK	67
Tc00.1047053506249.70	EAN84815.1	ABC transporter, putative	267	6	4	9	FGSTSGIQFQELFR	75
							GSVAQFDAVEQNK	18
							ILCGLTGTALPSR +Carbamidomethyl (C)	47
							ILLLDEPTSGLDSVTSVK	127
Tc00.1047053506445.60	EAN91964.1	mitochondrial DNA topoisomerase II, putative	250	6	4	4	DNKVIDTNR	16
							IDIVNATDDSLAK	89
							LLDSVFDSQEVEWR	87
							LVPWAVGYQGEVR	58
Tc00.1047053508741.170	EAN99198.1	hypothetical protein, conserved	245	6	3	10	GTLTACNAGLALER : Carbamidomethyl (C6)	77
							LIGDVANPLVGPDGATAIFGPQK	109

							LLGDVVVICGR : Carbamidomethyl (C9)	58
Tc00.1047053509463.30	EAN85395.1	3-ketoacyl-CoA thiolase, putative	244	7	4	14	GSPLFIDKK	36
							IPVNTGGGLLAFGHPVGATGVK	42
							SIDDFTFPCLFAK +Carbamidomethyl (C)	55
							SLAAASGNLYEDPPDATR	111
Tc00.1047053510797.30	EAN85334.1	hypothetical protein, conserved	243	8	4	8	EGGVELEPAQELPTDAFTLLR	78
							LLELYDQR	35
							LVLICDYDEFTR +Carbamidomethyl (C)	60
							SIAADSPLNIALLPEQVR	70
Tc00.1047053508177.10	EAN91138.1	hypothetical protein, conserved	239	5	4	7	FFVTGGNDR	26
							LALESVIGFGGR	73
							LVFAVNDAHKR	55
							TGEVNSLSISTDGR	85
Tc00.1047053511441.10	EAN83138.1	calpain cysteine peptidase, putative	237	19	6	5	AFLDQKPEGVPLR	53
							EIAALEESMNAR	72
							EIAALEESMNARAQELAR	41
							ICELYPEGIRDPVVPEK +Carbamidomethyl (C)	22
							QLLEK	26
							SWYPLLLEK	23
Tc00.1047053504949.30	EAN82006.1	succinate dehydrogenase, putative	234	17	5	32	EQFLGPAVLLQSYR	76
							FLNPAYASK	53
							NAAPKPETVFTAK	26
							PSAPLTGEVAR	26
							VPPPLDATIIKE	53
Tc00.1047053511531.50	EAN86006.1	glucosamine-6-phosphate isomerase, putative	231	4	4	19	AIAVAR	21
							IVISQDSDAVADYVASYIVDR	116
							SLNDETIASNAR	46
							TNFFDFVDIPEENR	48
Tc00.1047053506563.40	EAN94839.1	beta tubulin, putative	230	13	4	10	FPGQLNSDLR	46
							INVYFDEATGGR	67
							LAVNLVPFPR	50
							NSSYFIEWIPNNIK	67
Tc00.1047053507927.20	EAN87685.1	mitochondrial oligo_U binding protein TBRGG1, putative	227	5	5	9	AAPHLFVPEEPSLQLIQR	45
							AIDTVGFHTDEQVGEILESVAGYR	28
							CAALAEALAEQK +Carbamidomethyl (C)	16
							EFLEEFLGTVK	45
							SDETTWQELTNAVLSK	93
Tc00.1047053507009.10	EAN90052.1	Gim5A protein, putative	226	11	4	20	ALLPEDAEKK	40
							GVLNSGLTR	20
							LSLLLNALSSK	60
							NAGCDSLAESLGNLSK +Carbamidomethyl (C)	106
Tc00.1047053509701.10	EAN81265.1	trifunctional enzyme alpha subunit, mitochondrial precursor-like protein, putative	226	11	3	7	AGAVNVVIPAEDR	65
							GGLLQYADHR	50
							LLASDGSVGLPECLLGVLPGAGGTVR +Carbamidomethyl (C)	111
Tc00.1047053510395.10	EAN81916.1	ATP synthase, alpha chain, mitochondrial precursor, putative	216	10	3	15	SPVNYNLLTGFK	71
							TSIAVSTIINQVR	68
							VDTGAPNIVSR	77
Tc00.1047053508737.100	EAN95823.1	ATP-dependent Clp protease subunit, heat shock protein 78, putative	214	7	5	9	ADSFLTHDSVTASDIAQVIAR	17
							FQSVLVTEPTVEETISVLR	72
							IAERQDEYDALEAR	20
							LVQSVLLNR	54
							TAIVEGLAQR	51
Tc00.1047053510003.20	EAN87164.1	hypothetical protein, conserved	211	18	5	3	GLQDAEALLR	73
							RPEELAVIR	61
							RPEELAVIRIEGDK	40

							VLVYQLAQK	20
							VLVYQLAQKYFPK	17
Tc00.1047053509777.130	EAN92382.1	hypothetical protein, conserved	210	6	3	7	DELYLPVR	27
							LSAFNDCPDAAADYPFK +Carbamidomethyl (C)	110
							SALFFTFPLQGYPER	73
Tc00.1047053509215.40	EAN86719.1	cyclophilin, putative	206	23	5	22	FPDESFAGR	63
							GYEVIEK	29
							HVVFGQITK	41
							LPFYPINPK	36
							QPILITDCGEVK +Carbamidomethyl (C)	37
Tc00.1047053511575.130	EAN91522.1	folate/pteridine transporter, putative	205	4	3	6	ASAAPAAAFVFLTSFTK	91
							ESLSAQEPVLK	52
							ICDNIDVDGR +Carbamidomethyl (C)	62
Tc00.1047053510773.20	EAN91609.1	vacuolar-type proton translocating pyrophosphatase 1, putative	204	12	3	4	AADVGADLVGK	65
							EITDALDAAGNTTAAIGK	113
							NVYVISR	26
Tc00.1047053506577.120	EAN95037.1	sterol C-24 reductase, putative	197	5	4	9	AFPQLPWGHIENPR	62
							GELFVDGWYR	29
							ISPEESYNLVVER	83
							LVPYK	23
Tc00.1047053508707.310	EAN98870.1	hypothetical protein, conserved	197	7	5	7	CVLEKPIEPSTPEIHDLK +Carbamidomethyl (C)	21
							NDWVAGEPK	41
							NVLTEGIPVR	34
							VLDLSINEIGGTVDFLSK	79
							YVPTLEDVGK	22
Tc00.1047053508815.179	EAN93755.1	dynein heavy chain, putative	194	5	4	2	TMTQLSKTFK	19
							VNVVAVGPTGTGK	52
							YLEPIFSSEDIALQLPR	92
							YLPAEGIYEFINEDFR	31
Tc00.1047053508503.20	EAN86560.1	cytochrome c oxidase subunit V, putative	191	4	3	17	DADVLR	30
							GAASLLESLYR	74
							GADIPDHVFQTPAVIER	87
Tc00.1047053506275.20	EAN88322.1	hslvu complex proteolytic subunit-like, putative	188	7	4	20	AAVELAK	22
							ALIDVDGYDAEK	71
							KGDTVVLIGDR	30
							VGEFPQQLTR	66
Tc00.1047053509799.140	EAN92352.1	hypothetical protein, conserved	187	6	4	7	GQNNPLILNLR	43
							IIVAANELEIPAVQR	61
							TIAAK	34
							TVLSATLKPK	49
Tc00.1047053511389.150	EAN92450.1	thiolase protein-like protein, putative	182	5	4	12	GLAALPAFFK	48
							LGTAAVSGLLR	31
							LVAPATLSYGLAR	75
							NGETVEFVKDEYIQSDIEK	28
Tc00.1047053506223.80	EAN91087.1	ATP-dependent zinc metallopeptidase, putative	180	5	3	6	AYALELAER	21
							EAAPCVVFIDEIDAIGSR +Carbamidomethyl (C)	76
							TINQLLAELDGLSSK	83
Tc00.1047053508241.130	EAN91484.1	hypothetical protein, conserved	173	7	5	5	EIVELVVSK	20
							ELSPEGLAAFLR	62
							ILSKK	15
							KEELLHLPLDSIITR	17
							TPSEVLPGAGLLALK	59
Tc00.1047053506563.79	EAN88765.1	calpain-like cysteine peptidase (pseudogene), putative	167	11	3	20	GDEIVK	32
							GFDEGNGLLFR	74
							VTFGEDCDIK +Carbamidomethyl (C)	61

Tc00.1047053509911.74	EAN91357.1	hypothetical protein, conserved	163	5	3	17	ADQQLQLILDK	70
							GEYTIPTADILR	52
							QCYVNVPATK +Carbamidomethyl (C)	41
Tc00.1047053511277.170	EAO00034.1	ATP-dependent zinc metallopeptidase, putative	163	3	2	8	ELADLTPGVSPATIATIVNEAALQSGIR	52
							SSAPAIVFIDEIDAIGSR	111
Tc00.1047053508231.40	EAN96755.1	hypothetical protein, conserved	161	11	3	11	FGQAIINCR +Carbamidomethyl (C)	67
							LVSLYSQNEETSR	78
							SVAGLSR	16
Tc00.1047053507941.150	EAN90974.1	histone H4, putative	160	8	3	31	ILYGYA	32
							ISGVIYDEVR	54
							TVTAVDVVNALR	75
Tc00.1047053506563.170	EAN96755.1	calpain-like cysteine peptidase (pseudogene), putative	159	8	4	27	TAANEQEFR	58
							TAANEQEFRL	63
							VDDPEVGKEDDVGK	22
							VTGWTVDALEAR	16
Tc00.1047053508981.39	EAN84570.1	trifunctional enzyme alpha subunit, mitochondrial precursor-like protein	159	3	2	5	ACVAIIASAK +Carbamidomethyl (C)	48
							LLASDGSVGLPECLLGVLPGAGGTVR +Carbamidomethyl (C)	111
Tc00.1047053506989.190	EAN96800.1	lipophosphoglycan biosynthetic protein, putative	157	5	3	5	AELEEHLGSLGTSGTKR	41
							HIYFLTGDSVK	49
							LGILEDANNR	67
Tc00.1047053511217.90	EAN96478.1	hypothetical protein, conserved	152	6	4	3	ENWVSLIQGR	41
							GLPFITPLYTR	27
							IPPFGTTIGIGER	46
							SVAEPIGTVEQVR	38
Tc00.1047053507501.10	EAN84826.1	retrotransposon hot spot (RHS) protein, putative	151	4	3	3	DILLLILGSR	63
							DILLLILGSRGVLASK	37
							GYIIYDVAK	51
Tc00.1047053510533.210	EAN95652.1	hypothetical protein, conserved (pseudogene)	148	18	4	2	GLQEVSEQAEDLQR	88
							LEAKNTDKTK	20
							NLEEELHK	15
							QLEELRAENEELR	25
Tc00.1047053506147.80	EAN95743.1	hypothetical protein, conserved	146	2	2	17	ALDEEIK	21
							VAFGAANDVVGEEGLNEASVR	125
Tc00.1047053506551.10	EAN81429.1	hypothetical protein, conserved	145	3	2	4	LLYGEWYTR	59
							TGEQFVEVLATGPQK	86
Tc00.1047053508999.250	EAN93121.1	calpain-like cysteine peptidase (pseudogene), putative	142	6	3	26	FFVGDVTGFESK	60
							NTGTWAFFNNSR	63
							YYRRLIPPSAATF	19
Tc00.1047053511211.160	EAN95886.1	heat shock protein 70 (HSP70), putative	140	5	3	8	IINEPTAAAIAYGLDK	22
							SINPDEAVAYGAAVQAFILTGGK	53
							TTPSYVAFTDTER	65
Tc00.1047053504147.70	EAN97979.1	hypothetical protein, conserved	139	4	3	24	EANIEQGVAFLK	74
							VLTGELHFK	30
							YVAEEAVNANWKPADVKK	35
Tc00.1047053507093.260	EAN98703.1	ABC transporter, putative	136	6	2	3	LACIGYAGER +Carbamidomethyl (C)	67
							LSNVSFAYPTR	69
Tc00.1047053505945.20	EAN87195.1	ribonuclease mar1, putative	135	19	2	12	LPPDAHVFSK	44
							VANSANCVFVANR +Carbamidomethyl (C)	91
Tc00.1047053509203.40	EAN88868.1	glycosomal membrane protein, putative	132	4	3	11	ALAALDAVECR +Carbamidomethyl (C)	80
							SALINLTK	29
							SVFAK	23
Tc00.1047053503837.10	EAN84707.1	hypothetical protein, conserved	131	3	2	4	TGETICEGAVADGNDLR +Carbamidomethyl (C)	112
							VQLVDDLFR	19
Tc00.1047053507711.200	EAN98015.1	hypothetical protein, conserved	131	3	3	2	CPLFVSGVDPR +Carbamidomethyl (C)	30
							ELFCVLSIPR +Carbamidomethyl (C)	40

							VPVGWTFITGGK	61
Tc00.1047053510353.30	EAN83974.1	paraflagellar rod component Par4, putative	128	3	2	5	EEVLQLETK	41
							ISLEGELQNTLVELEANQK	87
Tc00.1047053508723.70	EAN91302.1	hypothetical protein, conserved	127	4	3	2	ILYDPVAYALIAVAK	59
							LLGEGVFPEK	35
							LYFFENEASLIR	33
Tc00.1047053511003.190	EAN94197.1	hypothetical protein, conserved	126	6	3	11	GGVPIIFPQFGNR	38
							GPLPAHGFAR	31
							SAISLLDQAR	57
Tc00.1047053457251.10	EAN81369.1	3-oxo-5-alpha-steroid 4-dehydrogenase, putative	124	8	3	11	DLGPQVGYR	43
							ELESLFVHK	27
							LFISLFAAHFVK	54
Tc00.1047053509797.40	EAN86703.1	isoleucyl-tRNA synthetase, putative	120	2	2	2	SVSGGGPLENFDEELNFSK	87
							YEPLFPYFK	33
Tc00.1047053506755.20	EAN96891.1	paraflagellar rod component, putative	118	2	2	7	DNLSALKPHQPPDLAR	46
							GVSGVINALNATQDAGEQLFQSVEK	72
Tc00.1047053508647.200	EAN96402.1	triosephosphate isomerase, putative	118	2	2	8	FQIAAQNAITR	68
							LGADIAAQLR	50
Tc00.1047053506839.70	EAN90346.1	NADH dehydrogenase, putative	116	4	2	4	IQPALATLPNR	35
							LPLPTLAAVASR	81
Tc00.1047053506201.170	EAN91538.1	hypothetical protein, conserved	115	4	3	4	ELEEALVALSAEK	38
							LADELEQK	48
							LAEELEQK	29
Tc00.1047053506977.60	EAN90032.1	hypothetical protein, conserved	112	1	1	15	TNHGTAAFNEGETAGPQ	112
Tc00.1047053507711.60	EAN98001.1	hypothetical protein, conserved	111	1	1	2	NIANEAFLLENDLIR	111
Tc00.1047053509051.20	EAN84309.1	amastin, putative	111	3	2	13	ACNSPNYDWR +Carbamidomethyl (C)	55
							LSPGTCYTLWGTR +Carbamidomethyl (C)	56
Tc00.1047053503769.40	EAN84925.1	cytochrome c oxidase VII, putative	109	7	3	15	ARVPNLSAFSLKWIYSAK	17
							DAVDTVR	41
							VPNLSAFSLK	51
Tc00.1047053503793.10	EAN81565.1	2-oxoglutarate dehydrogenase subunit, putative	106	1	1	5	LEQLSPFPWEQVADVLEK	106
Tc00.1047053504069.80	EAN89661.1	ATP synthase F1 subunit gamma protein, putative	106	1	1	5	LVAVEGQLTNISTLK	106
Tc00.1047053509053.70	EAN91460.1	p22 protein precursor, putative	106	5	2	8	LALDDSVEGNR	87
							QWLYKGPK	19
Tc00.1047053503887.40	EAN85439.1	hypothetical protein, conserved	105	2	2	11	EKLDAAGVETDAQ	72
							LDAAGVETDA	33
Tc00.1047053508275.9	EAN82251.1	dynein heavy chain, putative	103	4	2	1	LQAEAEGIAAR	31
							VLTPILLALTNPK	72
Tc00.1047053504089.50	EAN90808.1	flagellar radial spoke component, putative	102	4	2	5	LLPDVTPQQILAGR	44
							WPGAVAFAAEGGK	58
Tc00.1047053508045.70	EAN92392.1	hypothetical protein, conserved	102	2	2	4	AAWSK	15
							LPLECILEVDSLR +Carbamidomethyl (C)	87
Tc00.1047053509233.180	EAN95983.1	ATPase beta subunit, putative	102	2	1	3	LADQAAEDTILTTGIK	102
Tc00.1047053509965.394	EAN98408.1	amastin, putative	98	3	2	15	GFETLAGR	26
							GYLDGGK	27
							LSCVTAWGVK : Carbamidomethyl (C3)	45
Tc00.1047053507047.150	EAN94531.1	hypothetical protein, conserved	97	3	3	7	EAYGQNWEEFLQLR	61
							LNRILSVDCER	15
							YYTWGPAQLK	21
Tc00.1047053509679.9	EAN81004.1	leucyl-tRNA synthetase, putative	97	3	2	10	AVGEGALPQEYTLVK	61
							LLCLEAAAQER +Carbamidomethyl (C)	36
Tc00.1047053506355.10	EAN92970.1	hexose transporter, putative	97	3	2	4	QSPIEVATPGNR	50
							WVYSDEECK +Carbamidomethyl (C)	47
Tc00.1047053506657.40	EAN87278.1	ADP/ATP translocase, putative	97	3	2	6	MTLLSTSVR	33
							QDGITAFWAGLR	64

Tc00.1047053503893.30	EAN93234.1	hypothetical protein, conserved	95	26	3	15	FQTDSDWTIEK	34
							QTVLQTLTSR	40
							SIYTY	21
Tc00.1047053511809.130	EAN91806.1	40S ribosomal protein S15, putative	95	1	1	11	GLEIDPLLALSEEEFK	95
Tc00.1047053509733.170	EAN96827.1	transitional endoplasmic reticulum ATPase, putative	95	3	2	3	AAAPCVLFFDELDSVAR +Carbamidomethyl (C)	73
							VAIIK	22
Tc00.1047053506587.70	EAN88974.1	hypothetical protein, conserved	95	6	2	9	EGFVTCDVR : Carbamidomethyl (C6)	34
							SGSYELSDLLGLK	62
Tc00.1047053511733.90	EAN88212.1	hypothetical protein, conserved	93	1	1	15	IPTGVSTSSSPSKDPVK	93
Tc00.1047053507053.180	EAN95853.1	hypothetical protein, conserved	92	2	2	4	DAGATIPQVIASFFSR	70
							SGTVHLLNAANGEIER	22
Tc00.1047053508547.160	EAN95112.1	hypothetical protein, conserved	92	4	3	5	DLESLSHDTQNTEEHIK	27
							EALSSIEQTLR	50
							SCEESLWR +Carbamidomethyl (C)	15
Tc00.1047053509109.30	EAN94820.1	hypothetical protein, conserved	92	4	2	3	HSQNLQLAR	19
							YPLLAVPVVQELLR	73
Tc00.1047053503413.4	EAN83891.1	hypothetical protein, conserved	89	1	1	2	ALESSDPAGGEAEPWQNSVR	89
Tc00.1047053511773.110	EAN89238.1	retrotransposon hot spot (RHS) protein, putative	89	2	1	2	YSAASSIVDILDGFSDR	89
Tc00.1047053508173.264	EAN96436.1	hypothetical protein, conserved	89	3	2	6	FLIFADAVSR	53
							VFPNHGFTSR	36
Tc00.1047053510855.10	EAN83377.1	peptide methionine sulfoxide reductase, putative	89	1	1	7	AFGDAQVVTSLEK	89
Tc00.1047053506025.14	EAN86477.1	ribosomal protein S29, putative	88	4	2	31	ENAANIGFSK	48
							KYELNVCR +Carbamidomethyl (C)	40
Tc00.1047053510529.30	EAN84533.1	hypothetical protein, conserved	88	5	2	2	VANLQQLEQALK	31
							VVPGVDEDELLR	57
Tc00.1047053445777.10	EAN80690.1	retrotransposon hot spot (RHS) protein, putative	87	5	2	10	GYIIYDVAK	51
							MGAVNDALLGIKPNDAEK	36
Tc00.1047053503815.10	EAN82998.1	alkyl-dihydroxyacetone phosphate synthase, putative	87	1	1	2	LQQPFINQTFLEELR	87
Tc00.1047053508719.30	EAN87059.1	hypothetical protein, conserved	87	5	3	13	AQKKAK	20
							HVPSSFLLK	17
							SVGLTAALSPK	50
Tc00.1047053511151.90	EAN90136.1	glycerol-3-phosphate dehydrogenase, putative	87	2	1	2	TVLNAGGPFSEEVQK	87
Tc00.1047053506219.40	EAN82739.1	hypothetical protein, conserved	85	6	2	8	ILPESLVANK	46
							TAGHINHALLNSCLR +Carbamidomethyl (C)	39
Tc00.1047053511029.20	EAN81680.1	kinetoplast DNA-associated protein, putative	85	4	2	9	LSALK	23
							VSPYSIFLQELAR	62
Tc00.1047053509153.120	EAN93479.1	acyl-CoA dehydrogenase, putative	85	3	2	10	AFDFTRPPVAIGAVAVAQR	51
							GIVFEDVVIPEANVLGKPGDGFK	34
Tc00.1047053504103.20	EAN90017.1	hypothetical protein, conserved	80	1	1	1	ATAAQVAAVISSFGR	80
Tc00.1047053509317.80	EAN87341.1	hypothetical protein, conserved	80	3	1	9	NWDYSQVNEGIWK	80
Tc00.1047053506893.100	EAN85988.1	hypothetical protein, conserved	80	1	1	5	TLIVLEGIDGVGK	80
Tc00.1047053509793.50	EAN85214.1	hypothetical protein, conserved	78	2	2	9	AQKKAK	20
							SVGLTAALSPR	58
Tc00.1047053506583.60	EAN84717.1	mitochondrial elongation factor G, putative	78	2	1	2	NATQELWETLLPK	78
Tc00.1047053506503.140	EAN95091.1	extracellular receptor, putative	75	2	2	4	ACSVLLGPGR +Carbamidomethyl (C)	60
							QDELFLLGSREAVNVLAFLQGSFQPLVK	15
Tc00.1047053503571.19	EAN83327.1	hypothetical protein, conserved	74	1	1	1	LTDEQLVEVR	74
Tc00.1047053504125.50	EAN91369.1	mitochondrial carrier protein, putative	74	6	1	5	ALNNLTGSATGVQK	74
Tc00.1047053511071.130	EAN92157.1	basal body component, putative	74	1	1	0.9	LSEVCEALEQER : Carbamidomethyl (C5)	74
Tc00.1047053506519.130	EAN91257.1	inosine-5'-monophosphate dehydrogenase, putative	74	3	1	2	IGVGPGSICTTR +Carbamidomethyl (C)	74
Tc00.1047053503959.10	EAN87033.1	hypothetical protein, conserved	73	1	1	3	LLVLDLPLVAPAFIAK	73
Tc00.1047053506445.110	EAN91969.1	nucleobase transporter, putative	71	1	1	2	ASLPQDYSGIR	71
Tc00.1047053511635.10	EAN83533.1	histone H2B, putative	71	4	1	12	IVNSFVNDLFER	71
Tc00.1047053503999.30	EAN91585.1	hypothetical protein, conserved	70	4	2	2	LDELAAAVAK	55
							SSATIIEEILRK	15

Tc00.1047053510155.70	EAN98261.1	heat shock protein 70 (HSP70), putative	70	2	2	2	NDIETLILHYK	52
							VTVRILGGKNK	18
Tc00.1047053433273.10	EAN80780.1	dynein heavy chain, putative	69	1	1	3	SLLDTWSGILR	69
Tc00.1047053504153.250	EAN99067.1	hypothetical protein, conserved	69	2	1	3	STVNAIESVVEK	69
Tc00.1047053508153.1100	EAO00253.1	MP99, putative	66	2	1	1	AELIEQIVPVFTR	66
Tc00.1047053503843.40	EAN84765.1	chaperone DnaJ protein, putative	65	1	1	2	LFLAALPQAIFFR	65
Tc00.1047053506679.100	EAN98186.1	40S ribosomal protein S18, putative	64	1	1	8	AGTLTAEELER	64
Tc00.1047053506983.39	EAN85448.1	calpain-like cysteine peptidase, putative	64	5	2	17	DNGNGLLFR	18
							LNFEANPVAK	46
Tc00.1047053509045.20	EAN81815.1	co-chaperone GrpE, putative	63	2	2	7	EVLYR	20
							VSTEEIESNK	43
Tc00.1047053508719.40	EAN87060.1	kinetoplast DNA-associated protein, putative	62	1	1	10	NNPALSGLPISER	62
Tc00.1047053510089.210	EAN94958.1	hypothetical protein, conserved	62	1	1	6	ALGEYYFQR	62
Tc00.1047053504147.120	EAN97983.1	60S ribosomal protein L22, putative	61	1	1	8	YFNIQDQEEA	61
Tc00.1047053506375.90	EAN90829.1	hypothetical protein, conserved	61	2	2	13	EVTLYPIVNFAAPHYFR	40
							QNPPGLVCTIR +Carbamidomethyl (C)	21
Tc00.1047053507777.20	EAN84069.1	hypothetical protein, conserved	60	8	1	11	ALKAEEAPVEK	60
Tc00.1047053506401.70	EAN99410.1	vacuolar protein sorting protein 18, putative	60	5	3	2	AVEIR	27
							HMLWKRLATATAK	16
							TYWLKYASILMRFCPCR +Carbamidomethyl (C)	17
Tc00.1047053508153.270	EAO00223.1	heat shock protein 20, putative	60	3	1	8	LPSPLEEGSVK	60
Tc00.1047053504037.30	EAN82434.1	60S ribosomal protein L12, putative	59	1	1	9	AVGGEVPATASLAPK	59
Tc00.1047053507713.30	EAN82629.1	heat shock protein 85, putative	59	1	1	2	GVVDSEDLPLNISR	59
Tc00.1047053503903.60	EAN85769.1	hypothetical protein, conserved	58	1	1	9	NDVDITDPK	58
Tc00.1047053510119.20	EAN84978.1	elongation factor 1-alpha (EF-1-alpha), putative	58	16	1	2	IGGIGTVPVGR	58
Tc00.1047053506933.60	EAN90161.1	mitochondrial RNA binding protein, putative	57	3	1	8	GFGFIEDDTDKK	57
Tc00.1047053508413.68	EAN87014.1	kinetoplastid membrane protein KMP-11	57	3	1	10	FAELLEQQK	57
Tc00.1047053509561.20	EAN90705.1	flagellum-adhesion glycoprotein, putative	57	1	1	2	QQLVIQDFFISR	57
Tc00.1047053407477.50	EAN83385.1	cytochrome c oxidase VIII (COX VIII), putative	57	8	1	7	GSDVWAADGK	57
Tc00.1047053504427.64	EAN97645.1	hypothetical protein, conserved	56	3	1	12	LQENYDAGVER	56
Tc00.1047053506295.70	EAN91006.1	hypothetical protein, conserved	56	4	1	8	RLEYETVESK	56
Tc00.1047053508719.60	EAN87062.1	kinetoplast DNA-associated protein, putative	55	3	1	9	NNPALSGLPVAK	55
Tc00.1047053511529.160	EAN93883.1	enoyl-CoA hydratase/isomerase family protein, putative	55	1	1	3	LLWEDFTNTR	55
Tc00.1047053506963.14	EAN89730.1	40S ribosomal protein S27, putative	55	1	1	15	GFFDSDLSYPTVR	55
Tc00.1047053508153.130	EAO00193.1	enoyl-CoA hydratase, mitochondrial precursor, putative	55	1	1	4	GAVVTLTLNRPK	55
Tc00.1047053508699.130	EAN90805.1	cation transporter, putative	55	2	1	5	LSPFLFVLGK	55
Tc00.1047053506195.110	EAN97253.1	malate dehydrogenase, putative	55	1	1	4	LFGVTTLDLVR	55
Tc00.1047053503449.14	EAN83027.1	hypothetical protein, conserved	53	1	1	5	LLELYPSGK	53
Tc00.1047053504153.160	EAN99058.1	carboxypeptidase, putative	52	1	1	3	AWELENLLPEEFVER	52
Tc00.1047053506579.10	EAN89676.1	ABC transporter, putative	52	2	1	1	QDEFLESQLK	52
Tc00.1047053507715.34	EAN90878.1	hypothetical protein, conserved	52	1	1	6	YLASGEYFR	52
Tc00.1047053511751.200	EAN96655.1	epsilon-adaptin, putative	52	4	3	2	FLSARKANLR	17
							KKLTVR	20
							QIGEHRLSR	15
Tc00.1047053504105.130	EAN97848.1	calcium channel protein, putative	51	4	3	2	ALTGGRTPQELEDKNR	15
							DDNAMYEEALLFDR	18
							LPGLYQPAIDEK	18
Tc00.1047053507711.300	EAN98025.1	hypothetical protein, conserved	50	1	1	10	TPNFGLQVPER	50
Tc00.1047053511071.190	EAN92161.1	hypothetical protein, conserved	49	1	1	8	FFEDVPDAWSNEK	49
Tc00.1047053504069.50	EAN89658.1	peroxin 14, putative	48	1	1	3	VSSAVQFLHDSR	48
Tc00.1047053504163.60	EAN87520.1	hypothetical protein, conserved	48	1	1	6	LTAEQLQDQIR	48
Tc00.1047053506163.50	EAN88337.1	hypothetical protein, conserved	48	1	1	3	QGLGSEVAEVK	48
Tc00.1047053455721.9	EAN81337.1	cytochrome c oxidase subunit 10, putative	47	2	1	9	INNNPTVGQFR	47
Tc00.1047053506949.50	EAN87335.1	cytochrome c, putative	46	1	1	10	HSGTVEGFAYSK	46
Tc00.1047053506779.120	EAN93692.1	hypothetical protein, conserved	46	3	1	5	GGFTSPSFLR	46

Tc00.1047053506789.240	EAN98073.1	hypothetical protein, conserved	46	2	1	5	NANLITNVAGGK	46
Tc00.1047053506789.140	EAN98063.1	hypothetical protein, conserved	46	1	1	2	DAPVGAGVFLHR	46
Tc00.1047053504427.60	EAN97625.1	hypothetical protein, conserved	45	1	1	5	LAQPYFAAVLHELPFVR	45
Tc00.1047053506755.260	EAN96915.1	mitochondrial RNA-binding protein 2, putative	45	2	1	4	DSFVVLDYHR	45
Tc00.1047053508999.260	EAN98666.1	calpain-like cysteine peptidase (pseudogene), putative	45	1	1	8	TAANEQDFRL	45
Tc00.1047053506753.240	EAN93538.1	hypothetical protein, conserved	44	1	1	5	LPIPAEYVR	44
Tc00.1047053509611.170	EAN92954.1	hypothetical protein, conserved	44	1	1	2	GDTILLFLLR	44
Tc00.1047053504013.100	EAN88197.1	ribosomal protein S19, putative	43	1	1	7	APQNPDWYYIR	43
Tc00.1047053504035.84	EAN92237.1	hypothetical protein, conserved	43	2	1	10	GKECTVFYR +Carbamidomethyl (C)	43
Tc00.1047053506755.250	EAN96914.1	mitochondrial import inner membrane translocase subunit Tim17, putative	43	1	1	7	IIDALGFAQH	43
Tc00.1047053506475.116	EAN95349.1	glutaredoxin, putative	43	1	1	10	RGELSATLER	43
Tc00.1047053506629.40	EAN94726.1	hypothetical protein, conserved	43	1	1	3	LLPVSAPTPADYFFGR	43
Tc00.1047053509537.50	EAN89980.1	hypothetical protein, conserved	42	4	1	0	FGLQQQPVSR	42
Tc00.1047053399373.9	EAN82602.1	retrotransposon hot spot (RHS) protein, putative	42	1	1	2	FTISTNTEDVLFK	42
Tc00.1047053505169.10		polyubiquitin (pseudogene), putative	42	8	1	3	TLADYNIQK	42
Tc00.1047053408437.20	EAN82295.1	hypothetical protein, conserved	41	2	1	7	QETLDSLEK	41
Tc00.1047053511229.30	EAN87301.1	hypothetical protein, conserved	41	1	1	5	AQFHQAATHISLYR	41
Tc00.1047053506715.50	EAN84023.1	SNARE 2.2 (hypothetical protein)	37	5	2	10	EVEMFNDK	18
							KILANIKRPLVER	19
								25
								20
								18
								18
								17
								17
								17
								16
								16
								16
								16
								16
								15
								15
