# Supplementary material for: Identification of Contractile Vacuole Proteins in Trypanosoma cruzi
Source: PLoS One. 2011 Mar 18;6(3):e18013. doi: 10.1371/journal.pone.0018013 (PMC3060929; doi:10.1371/journal.pone.0018013)
Supplement: Table S3 — Peptides identified in the proteomic analysis of the subcellular fraction that map to DGF-1 proteins in epimastigotes. This Table lists the peptides identified in the proteomic analysis that map to DGF-1 proteins in epimastigotes. (PDF) [file pone.0018013.s003.pdf]

Table S3. Peptides identified in the proteomic analysis of the subcellular fraction that map to DGF-1 proteins in epimastigotes. The proteins denoted with an asterisk were present in the selected group or 220 proteins identified with high confidence.

Gene ID number	Peptides
Tc00.1047053509961.70*	ASLYVVGWR; DGDCFAPLTTAVSDCK; ESPSDAFAYAYPR; EVSYDGVFPEK; HCVLVGDVQLR; SAGIIGK; VVASEGAVLR; YGPALVLDGVR
Tc00.1047053507153.30	DGDCFAPLTTAVSDCK; FVGVEGSAASSLVR; SKLVCGGGSCAAILVER; VVASEGAVLR; YGPALVLDGVR
Tc00.1047053507187.9*	DGDCFAPLTTAVSDCK; ESPSDAFAYAYPR; EVSYDGVFPEK; GASCLPFGVPDTVVPPLPER; YGPALVLDGVR
Tc00.1047053506005.10	DGDCFAPLTTAVSDCK; ESPSDAFAYAYPR; FVGVEGSAASSLVR; YGPALVLDGVR
Tc00.1047053510847.70	ASLYVVGWR; DGDCFAPLTTAVSDCK; FVGVEGSAASSLVR; YGPALVLDGVR
Tc00.1047053510235.10	FVGVEGSAASSLVR; SAGIIGK; YGPALVLDGVR
Tc00.1047053510497.199	DGDCFAPLTTAVSDCK; VSNTVLRSLHAGGSAVYVGGGVK; YGPALVLDGVR
Tc00.1047053511475.50	DGDCFAPLTTAVSDCK; MGSTGLSVPLR; YGPALVLDGVR
Tc00.1047053507167.169	MWCAVGACPHSR; VVASEGAVLR; YGPALVLDGVR
Tc00.1047053503495.10	ENGVTFRDR; VVASEGAVLR; YGPALVLDGVR
Tc00.1047053509735.84	DAVGVLLVGGVALHSR; EGDCFAPLTTAVIDCR; SVTPTWTPR; YGPALVLDGVR
Tc00.1047053506339.100	DGDCFAPLTTAVSDCK; DPQLGMQLSFVLVSK; LTGSVLTIAR
Tc00.1047053507643.50	FTVTRPIPRLGLGLR; LPDTRITTLSVVMLR; YGPALVLDGVR
Tc00.1047053510713.20	DGDCFAPLTTAVSDCK; DVVVDGGSVLQIVSGIFR; SAGIIGK
Tc00.1047053508139.210	EGDCFAPLTTAVIDCR; SAGIIGK; YGPALVLDGVR
Tc00.1047053507179.20	SNTIVESPAR; YGPALVLDGVR
Tc00.1047053507243.9	FVGVEGSAASSLVR; HGPVLVLDGVR
Tc00.1047053504639.19	DSSVDMCLGR; SMQSAVGACPHSRHR; YGPALVLDGVR

Tc00.1047053510391.10	ASSLLVSNVKAHATK; EGDCFAPLTTAVIDCR; YGPALVLDGVR
Tc00.1047053509397.9	GLCFVK; SKLVCGGGSCAAILVER; YGPALVLDGVR
Tc00.1047053509805.260	GHAGFRYGPALVLDGVR; RADCCAER; YGPALVLDGVR
Tc00.1047053508283.10	GHAGFRYGPALVLDGVR; NGGYFDVEK; YGPALVLDGVR
Tc00.1047053510971.10	LLYPVGYWHPAAEQR; YGPALVLDGVR
Tc00.1047053511771.70	QSFSLSVLNSSALVIR; YGPALVLDGVR
Tc00.1047053506183.20	EGDCFAPLTKAISDCK; YGPALVLDGVR
Tc00.1047053506173.140	MWCAVGACPRSRHPVLR; YGPALVLDGVR
Tc00.1047053507911.30	LTGSVLTIAR; YGPALVLDGVR
Tc00.1047053508163.190	CNAVAVSGGCK; YGPALVLDGVR
Tc00.1047053509925.70	FVVGCLMLNGQALQPMDYR; YGPALVLDGVR
Tc00.1047053509503.9	EDSRTTSAVYYNGVHLR; YGPALVLDGVR
Tc00.1047053509815.120	SQILVAGSK; YGPALVLDGVR
Tc00.1047053436137.9	HCGGLGADCGKTRCVPR; YGPALVLDGVR
Tc00.1047053509845.9	EGDCFAPLTTAVIDCR; SVTPTWTPR; VVASEGAVLR
Tc00.1047053510271.20	ATVGGSQYVPTTRGHAKSR; SNTIVESPAR
Tc00.1047053506651.9	FVVGCLTVAGR; SNTIVESPAR
Tc00.1047053510367.10	ALSSAICVLR
Tc00.1047053506885.489	LFNSNTIVALPAR
Tc00.1047053506045.19	QSLSLSVLNNSAVVIRGNAVLGGLK
Tc00.1047053433717.10	AHATRYDAFGLYSTGTLTLVR
