# Supplementary material for: Identification of Contractile Vacuole Proteins in Trypanosoma cruzi
Source: PLoS One. 2011 Mar 18;6(3):e18013. doi: 10.1371/journal.pone.0018013 (PMC3060929; doi:10.1371/journal.pone.0018013)
Supplement: Table S4 — Signal peptide (SP) and transmembrane domain (TM) predictions for proteins in the T. cruzi CV dataset. This Table lists SP and TM predictions for proteins in the CV dataset. (PDF) [file pone.0018013.s004.pdf]

Table S4. Signal peptide (SP) and transmembrane domain (TM) predictions for proteins in the *Trypanosoma cruzi* contractile vacuole dataset. Final predictions are based on consensus among 2 or more prediction algorithms. If TM domains are predicted, the final result given is the median of the number of predicted TM from the different algorithms. If annotated descriptions of gene products differ from the specific name used in the text, the annotated protein name is given in parenthesis.

		TMHMM	HMMTOP	Polyphobius		SignalP3	Final Prediction	
**Gene ID**	**Description**	**TM**	**TM**	**TM**	**SP**	**SP**	**SP**	**TM**
Tc00.1047053511909.40	succinate dehydrogenase flavoprotein, putative	0	1	0	N	N	N	0
Tc00.1047053511903.40	hypothetical protein, conserved	0	0	0	N	N	N	0
Tc00.1047053511773.110	retrotransposon hot spot (RHS) protein, putative	0	0	0	N	Y	N	0
Tc00.1047053511751.200	epsilon-adaptin, putative	0	1	0	N	N	N	0
Tc00.1047053511733.90	hypothetical protein, conserved	0	0	0	N	N	N	0
Tc00.1047053511575.130	folate/pteridine transporter, putative	12	12	12	N	N	N	12
Tc00.1047053511531.50	glucosamine-6-phosphate isomerase, putative	0	0	0	N	N	N	0
Tc00.1047053511529.160	enoyl-CoA hydratase/isomerase family protein, putative	0	0	0	N	N	N	0
Tc00.1047053511527.70	myosin heavy chain, putative	0	1	0	N	N	N	0
Tc00.1047053511441.10	calpain cysteine peptidase, putative	0	1	0	N	N	N	0
Tc00.1047053511389.150	thiolase protein-like protein, putative	0	2	0	N	N	N	0
Tc00.1047053511289.70	ADP,ATP carrier protein 1, mitochondrial precursor, putative	3	4	2	N	N	N	3
Tc00.1047053511277.290	aconitase, putative	0	0	0	N	N	N	0
Tc00.1047053511277.170	ATP-dependent zinc metallopeptidase, putative	1	1	1	N	N	N	1
Tc00.1047053511269.60	vacuolar protein sorting complex subunit, putative	0	0	0	N	N	N	0
Tc00.1047053511229.30	hypothetical protein, conserved	2	3	2	N	N	N	2
Tc00.1047053511217.90	hypothetical protein, conserved	2	2	1	N	Y	N	2
Tc00.1047053511215.119	69 kDa paraflagellar rod protein, putative	0	0	0	N	N	N	0
Tc00.1047053511211.20	coatomer gamma subunit, putative	0	0	0	N	N	N	0
Tc00.1047053511159.7	neurobeachin/beige protein, putative	0	4	5	N	N	N	4
Tc00.1047053511151.90	glycerol-3-phosphate dehydrogenase, putative	0	1	0	Y	N	N	0
Tc00.1047053511071.190	hypothetical protein, conserved	4	4	4	N	Y	N	4
Tc00.1047053511029.20	kinetoplast DNA-associated protein, putative	0	0	0	N	N	N	0
Tc00.1047053511003.190	hypothetical protein, conserved	0	0	0	N	N	N	0
Tc00.1047053510943.50	delta-1-pyrroline-5-carboxylate dehydrogenase, putative	0	0	0	N	N	N	0
Tc00.1047053510855.10	peptide methionine sulfoxide reductase, putative	0	0	0	N	N	N	0
Tc00.1047053510837.20	glutamamyl carboxypeptidase, putative	0	0	0	N	N	N	0
Tc00.1047053510797.30	hypothetical protein, conserved	0	0	0	N	N	N	0
Tc00.1047053510773.20	vacuolar-type proton translocating pyrophosphatase 1, putative	16	17	13	Y	Y	Y	16
Tc00.1047053510679.40	hypothetical protein	0	0	0	N	N	N	0
Tc00.1047053510533.210	hypothetical protein, conserved (pseudogene)	0	0	0	N	N	N	0
Tc00.1047053510529.30	hypothetical protein, conserved	0	0	0	Y	Y	Y	0
Tc00.1047053510395.10	ATP synthase, alpha chain, mitochondrial precursor, putative	0	3	0	Y	N	N	0
Tc00.1047053510353.30	paraflagellar rod component Par4, putative	0	0	0	N	N	N	0
Tc00.1047053510215.10	NADH-dependent fumarate reductase, putative	0	0	0	N	N	N	0
Tc00.1047053510155.70	heat shock protein 70 (HSP70), putative	1	2	0	Y	N	N	1
Tc00.1047053510149.80	ABC transporter, putative	12	12	14	N	Y	N	12
Tc00.1047053510119.20	elongation factor 1-alpha (EF-1-alpha), putative	0	0	0	N	N	N	0
Tc00.1047053510101.140	pyruvate phosphate dikinase, putative	0	0	0	N	N	N	0
Tc00.1047053510089.210	hypothetical protein, conserved	0	1	0	N	N	N	0
Tc00.1047053510003.20	hypothetical protein, conserved	0	2	0	N	N	N	0
Tc00.1047053509999.90	carnitine/choline acetyltransferase, putative	0	0	0	N	N	N	0
Tc00.1047053509961.70	dispersed gene family protein 1 (DGF-1, pseudogene), putative	2	1	1	Y	Y	Y	1
Tc00.1047053509911.74	hypothetical protein, conserved	0	1	1	N	N	N	1
Tc00.1047053509805.40	Golvesin-1 (methyltransferase, putative)	0	0	0	Y	N	N	0
Tc00.1047053509799.140	hypothetical protein, conserved	4	4	4	N	Y	N	4
Tc00.1047053509797.40	isoleucyl-tRNA synthetase, putative	0	0	3	N	N	N	0
Tc00.1047053509793.50	hypothetical protein, conserved	0	0	0	Y	N	N	0
Tc00.1047053509777.130	hypothetical protein, conserved	1	3	2	N	N	N	2
Tc00.1047053509733.170	transitional endoplasmic reticulum ATPase, putative	0	0	0	N	N	N	0
Tc00.1047053509701.10	trifunctional enzyme alpha subunit, mitochondrial precursor-like protein, put	0	1	0	N	N	N	0
Tc00.1047053509683.10	hypothetical protein	2	2	1	Y	Y	Y	2
Tc00.1047053509679.9	leucyl-tRNA synthetase, putative	0	0	0	N	N	N	0
Tc00.1047053509617.20	paraflagellar rod protein 3, putative	0	0	0	N	N	N	0
Tc00.1047053509611.170	hypothetical protein, conserved	3	3	3	N	Y	N	3
Tc00.1047053509601.70	vacuolar proton translocating ATPase subunit A, putative	6	7	7	N	N	N	7
Tc00.1047053509585.10	dynein heavy chain, putative	0	0	0	N	N	N	0
Tc00.1047053509561.20	flagellum-adhesion glycoprotein, putative	1	2	1	Y	Y	Y	1
Tc00.1047053509551.30	mitochondrial phosphate transporter, putative	1	4	1	N	N	N	1
Tc00.1047053509537.50	hypothetical protein, conserved	0	0	0	N	N	N	0
Tc00.1047053509499.14	tryparedoxin peroxidase, putative	0	0	0	N	N	N	0
Tc00.1047053509463.30	3-ketoacyl-CoA thiolase, putative	0	1	0	N	N	N	0
Tc00.1047053509445.39	glutamate dehydrogenase, putative	0	0	0	Y	Y	Y	0

Tc00.1047053509351.10	delta-1-pyrroline-5-carboxylate dehydrogenase, putative	0	0	0	N	N		N	0
Tc00.1047053509317.80	hypothetical protein, conserved	2	2	2	N	N		N	2
Tc00.1047053509237.130	cytoskeleton-associated protein CAP5.5, putative	0	1	0	N	N		N	0
Tc00.1047053509233.180	ATPase beta subunit, putative	0	1	0	N	N		N	0
Tc00.1047053509215.40	cyclophilin, putative	1	1	0	Y	Y		Y	1
Tc00.1047053509203.40	glycosomal membrane protein, putative	3	4	2	N	N		N	3
Tc00.1047053509153.120	acyl-CoA dehydrogenase, putative	0	0	0	Y	N		N	0
Tc00.1047053509109.30	hypothetical protein, conserved	0	0	0	N	N		N	0
Tc00.1047053509053.70	p22 protein precursor, putative	0	0	0	Y	N		N	0
Tc00.1047053509051.20	amastin, putative	4	4	4	N	N		N	4
Tc00.1047053509045.20	co-chaperone GrpE, putative	0	0	0	N	N		N	0
Tc00.1047053508999.260	calpain-like cysteine peptidase (pseudogene), putative	0	0	0	N	N		N	0
Tc00.1047053508999.250	calpain-like cysteine peptidase (pseudogene), putative	0	0	0	N	N		N	0
Tc00.1047053508981.39	trifunctional enzyme alpha subunit, mitochondrial precursor-like protein	0	0	0	Y	Y		Y	0
Tc00.1047053508827.40	acyl-CoA dehydrogenase, putative	0	0	0	N	N		N	0
Tc00.1047053508815.179	dynein heavy chain, putative	0	0	0	N	N		N	0
Tc00.1047053508741.229	hypothetical protein, conserved	0	0	0	N	N		N	0
Tc00.1047053508737.210	hypothetical protein, conserved	0	0	0	N	N		N	0
Tc00.1047053508737.100	ATP-dependent Clp protease subunit, heat shock protein 78, putative	0	0	0	Y	Y		Y	0
Tc00.1047053508723.70	hypothetical protein, conserved	0	1	0	Y	Y		Y	0
Tc00.1047053508719.60	kinetoplast DNA-associated protein, putative	0	0	0	N	N		N	0
Tc00.1047053508719.40	kinetoplast DNA-associated protein, putative	0	0	0	N	N		N	0
Tc00.1047053508719.30	hypothetical protein, conserved	0	0	0	Y	N		N	0
Tc00.1047053508707.310	hypothetical protein, conserved	0	0	0	N	N		N	0
Tc00.1047053508699.130	cation transporter, putative	3	3	3	N	N		N	3
Tc00.1047053508647.200	triosephosphate isomerase, putative	0	0	0	N	N		N	0
Tc00.1047053508555.60	cytoskeleton-associated protein CAP5.5, putative	0	1	0	N	N		N	0
Tc00.1047053508547.160	hypothetical protein, conserved	0	0	0	N	N		N	0
Tc00.1047053508535.10	NADH-dependent fumarate reductase, putative	0	0	0	Y	Y		Y	0
Tc00.1047053508503.20	cytochrome c oxidase subunit V, putative	0	0	0	Y	N		N	0
Tc00.1047053508413.68	kinetoplastid membrane protein KMP-11	0	0	0	N	N		N	0
Tc00.1047053508275.9	dynein heavy chain, putative	0	0	0	N	N		N	0
Tc00.1047053508241.130	hypothetical protein, conserved	0	0	0	N	N		N	0
Tc00.1047053508231.40	hypothetical protein, conserved	0	3	4	N	N		N	3
Tc00.1047053508209.120	10 kDa heat shock protein, putative	0	0	0	N	N		N	0
Tc00.1047053508177.10	hypothetical protein, conserved	0	1	0	N	N		N	0
Tc00.1047053508173.264	hypothetical protein, conserved	0	3	2	N	N		N	2
Tc00.1047053508153.340	6-phospho-1-fructokinase, putative	0	2	0	N	N		N	0
Tc00.1047053508153.270	heat shock protein 20, putative	0	0	0	N	N		N	0
Tc00.1047053508153.130	enoyl-CoA hydratase, mitochondrial precursor, putative	0	0	0	N	N		N	0
Tc00.1047053508153.1100	MP99, putative	0	0	0	N	N		N	0
Tc00.1047053508045.70	hypothetical protein, conserved	4	2	3	Y	Y		Y	3
Tc00.1047053507927.20	mitochondrial oligo_U binding protein TBRGG1, putative	0	2	0	N	N		N	0
Tc00.1047053507777.20	hypothetical protein, conserved	0	0	0	N	N		N	0
Tc00.1047053507739.110	myosin IB heavy chain, putative	0	1	0	N	N		N	0
Tc00.1047053507715.34	hypothetical protein, conserved	1	1	1	N	N		N	1
Tc00.1047053507713.30	heat shock protein 85, putative	0	0	0	N	N		N	0
Tc00.1047053507711.60	hypothetical protein, conserved	0	0	0	N	N		N	0
Tc00.1047053507711.300	hypothetical protein, conserved	0	1	1	N	N		N	1
Tc00.1047053507711.200	hypothetical protein, conserved	2	2	1	Y	Y		Y	2
Tc00.1047053507689.30	glutamamyl carboxypeptidase, putative	0	0	0	N	N		N	0
Tc00.1047053507641.290	chaperonin HSP60, mitochondrial precursor	0	0	0	N	N		N	0
Tc00.1047053507625.183	SNARE 2.1 (hypothetical protein, conserved)	1	2	1	N	Y		N	1
Tc00.1047053507547.90	glycosomal phosphoenolpyruvate carboxykinase, putative	0	0	0	N	N		N	0
Tc00.1047053507501.10	retrotransposon hot spot (RHS) protein, putative	0	0	0	N	N		N	0
Tc00.1047053507187.9	dispersed gene family protein 1 (DGF-1), putative	0	0	0	N	N		N	0
Tc00.1047053507093.260	ABC transporter, putative	5	5	5	N	N		N	5
Tc00.1047053507089.270	dihydrolipoyl dehydrogenase, putative	0	0	0	N	N		N	0
Tc00.1047053507053.180	hypothetical protein, conserved	2	4	1	Y	Y		Y	2
Tc00.1047053507047.150	hypothetical protein, conserved	0	0	0	N	N		N	0
Tc00.1047053507029.30	heat shock 70 kDa protein, mitochondrial precursor, putative	0	0	0	Y	N		N	0
Tc00.1047053507009.10	Gim5A protein, putative	0	2	1	N	N		N	1
Tc00.1047053506989.190	lipophosphoglycan biosynthetic protein, putative	1	2	0	Y	Y		Y	1
Tc00.1047053506983.39	calpain-like cysteine peptidase, putative	0	0	0	N	N		N	0
Tc00.1047053506977.60	hypothetical protein, conserved	0	1	0	N	N		N	0
Tc00.1047053506963.14	40S ribosomal protein S27, putative	0	0	0	N	N		N	0
Tc00.1047053506957.110	SNF-7-like protein, putative	0	0	0	N	N		N	0
Tc00.1047053506949.50	cytochrome c, putative	0	0	0	N	N		N	0
Tc00.1047053506933.60	mitochondrial RNA binding protein, putative	0	0	0	N	N		N	0
Tc00.1047053506839.70	NADH dehydrogenase, putative	0	0	0	N	N		N	0
Tc00.1047053506839.30	hypothetical protein, conserved	0	0	0	N	N		N	0
Tc00.1047053506779.120	hypothetical protein, conserved	3	4	3	N	N		N	3

Tc00.1047053506755.260	mitochondrial RNA-binding protein 2, putative	0	1	0	N	N		N	0
Tc00.1047053506755.250	mitochondrial import inner membrane translocase subunit Tim17, putative	3	3	2	N	N		N	3
Tc00.1047053506755.20	paraflagellar rod component, putative	0	0	0	N	N		N	0
Tc00.1047053506753.240	hypothetical protein, conserved	0	2	0	N	N		N	0
Tc00.1047053506715.50	SNARE 2.2 (hypothetical protein)	1	1	1	N	N		N	1
Tc00.1047053506657.40	ADP/ATP translocase, putative	0	6	1	N	N		N	1
Tc00.1047053506585.40	glucose-regulated protein 78, putative	0	1	0	Y	Y		Y	0
Tc00.1047053506583.60	mitochondrial elongation factor G, putative	0	0	0	N	N		N	0
Tc00.1047053506579.10	ABC transporter, putative	6	7	7	N	N		N	7
Tc00.1047053506577.120	sterol C-24 reductase, putative	9	9	8	N	N		N	9
Tc00.1047053506563.79	calpain-like cysteine peptidase (pseudogene), putative	0	0	0	N	N		N	0
Tc00.1047053506563.40	beta tubulin, putative	0	0	0	N	N		N	0
Tc00.1047053506563.170	calpain-like cysteine peptidase (pseudogene), putative	0	0	0	N	N		N	0
Tc00.1047053506551.10	hypothetical protein, conserved	14	14	14	N	N		N	14
Tc00.1047053506519.130	inosine-5'-monophosphate dehydrogenase, putative	0	0	0	N	N		N	0
Tc00.1047053506503.140	extracellular receptor, putative	2	2	1	Y	Y		Y	2
Tc00.1047053506491.20	myosin heavy chain, putative	0	0	0	N	N		N	0
Tc00.1047053506445.60	mitochondrial DNA topoisomerase II, putative	0	1	0	N	N		N	0
Tc00.1047053506445.110	nucleobase transporter, putative	9	11	11	N	Y		N	11
Tc00.1047053506401.70	vacuolar protein sorting protein 18, putative	0	0	0	N	N		N	0
Tc00.1047053506355.60	small G-protein, putative	0	0	0	N	N		N	0
Tc00.1047053506355.10	hexose transporter, putative	12	12	11	N	N		N	12
Tc00.1047053506337.70	2-oxoglutarate dehydrogenase E1 component, putative	0	0	0	Y	N		N	0
Tc00.1047053506297.190	pyruvate phosphate dikinase, putative	0	0	0	N	N		N	0
Tc00.1047053506295.70	hypothetical protein, conserved	1	1	1	N	N		N	1
Tc00.1047053506289.80	Rab32 (small GTP-binding protein, putative)	0	0	0	N	N		N	0
Tc00.1047053506249.70	ABC transporter, putative	5	6	7	N	N		N	6
Tc00.1047053506247.220	histidine ammonia-lyase, putative	0	0	0	N	N		N	0
Tc00.1047053506223.80	ATP-dependent zinc metallopeptidase, putative	1	1	1	N	N		N	1
Tc00.1047053506219.40	hypothetical protein, conserved	1	2	0	Y	Y		Y	1
Tc00.1047053506211.160	ADP,ATP carrier protein 1, mitochondrial precursor, putative	3	4	2	N	N		N	3
Tc00.1047053506167.50	clathrin heavy chain, putative	0	0	0	N	N		N	0
Tc00.1047053506163.50	hypothetical protein, conserved	0	0	0	N	N		N	0
Tc00.1047053506147.80	hypothetical protein, conserved	0	2	2	Y	N		N	2
Tc00.1047053506025.50	V-H+-ATPase subunit B (vacuolar ATP synthase subunit B, putative)	0	0	0	N	N		N	0
Tc00.1047053506025.14	ribosomal protein S29, putative	0	0	0	N	N		N	0
Tc00.1047053505945.20	ribonuclease mar1, putative	0	0	0	N	N		N	0
Tc00.1047053505843.10	glutamate dehydrogenase, putative	0	0	0	N	N		N	0
Tc00.1047053505763.19	P-type H+-ATPase, putative	3	5	4	N	N		N	4
Tc00.1047053504949.30	succinate dehydrogenase, putative	0	0	0	N	N		N	0
Tc00.1047053504427.64	hypothetical protein, conserved	0	0	0	N	N		N	0
Tc00.1047053504427.60	hypothetical protein, conserved	4	6	3	Y	Y		Y	4
Tc00.1047053504163.60	hypothetical protein, conserved	0	0	0	N	N		N	0
Tc00.1047053504153.310	heat shock protein, putative	0	1	0	N	N		N	0
Tc00.1047053504153.250	hypothetical protein, conserved	6	6	5	N	N		N	6
Tc00.1047053504153.160	carboxypeptidase, putative	0	0	0	N	N		N	0
Tc00.1047053504147.70	hypothetical protein, conserved	0	1	0	Y	Y		Y	0
Tc00.1047053504147.120	60S ribosomal protein L22, putative	0	0	0	N	N		N	0
Tc00.1047053504125.50	mitochondrial carrier protein, putative	2	6	2	N	Y		N	2
Tc00.1047053504105.130	calcium channel protein, putative	20	22	22	N	N		N	22
Tc00.1047053504103.20	hypothetical protein, conserved	0	5	0	Y	Y		Y	0
Tc00.1047053504089.50	flagellar radial spoke component, putative	0	1	0	N	N		N	0
Tc00.1047053504087.20	hypothetical protein	2	2	1	Y	Y		Y	2
Tc00.1047053504069.80	ATP synthase F1 subunit gamma protein, putative	0	0	0	N	N		N	0
Tc00.1047053504069.50	peroxin 14, putative	0	1	0	N	N		N	0
Tc00.1047053504037.30	60S ribosomal protein L12, putative	0	0	0	N	N		N	0
Tc00.1047053504035.84	hypothetical protein, conserved	1	1	1	N	N		N	1
Tc00.1047053504013.100	ribosomal protein S19, putative	0	0	0	N	N		N	0
Tc00.1047053503999.30	hypothetical protein, conserved	2	2	1	N	Y		N	2
Tc00.1047053503959.10	hypothetical protein, conserved	0	2	0	N	Y		N	0
Tc00.1047053503903.60	hypothetical protein, conserved	0	0	1	N	N		N	0
Tc00.1047053503899.119	trypanothione/tryparedoxin dependent peroxidase 2, putative	0	0	0	N	N		N	0
Tc00.1047053503893.30	hypothetical protein, conserved	2	0	2	N	N		N	2
Tc00.1047053503887.40	hypothetical protein, conserved	0	1	1	Y	Y		Y	1
Tc00.1047053503849.60	NADH-dependent fumarate reductase, putative	0	3	0	Y	Y		Y	0
Tc00.1047053503843.40	chaperone DnaJ protein, putative	0	5	0	Y	N		N	0
Tc00.1047053503837.10	hypothetical protein, conserved	14	14	14	N	Y		N	14
Tc00.1047053503815.10	alkyl-dihydroxyacetone phosphate synthase, putative	0	0	0	N	N		N	0
Tc00.1047053503793.10	2-oxoglutarate dehydrogenase subunit, putative	0	0	0	N	N		N	0
Tc00.1047053503769.40	cytochrome c oxidase VII, putative	0	1	1	N	N		N	1
Tc00.1047053503571.19	hypothetical protein, conserved	2	2	1	Y	Y		Y	2
Tc00.1047053503455.30	Golvesin-2 (hypothetical protein, conserved)	0	0	0	N	N		N	0

Tc00.1047053503449.30	AP180 (clathrin coat assembly protein, putative)	0	1	0	N	N		N	0
Tc00.1047053503449.14	hypothetical protein, conserved	0	0	0	Y	Y		Y	0
Tc00.1047053503413.4	hypothetical protein, conserved	0	0	0	N	N		N	0
Tc00.1047053457251.10	3-oxo-5-alpha-steroid 4-dehydrogenase, putative	4	6	6	N	N		N	6
Tc00.1047053455721.9	cytochrome c oxidase subunit 10, putative	0	0	0	N	N		N	0
Tc00.1047053445777.10	retrotransposon hot spot (RHS) protein, putative	0	0	0	N	N		N	0
Tc00.1047053433273.10	dynein heavy chain, putative	0	2	0	N	N		N	0
Tc00.1047053411235.9	alpha tubulin, putative	0	0	0	N	N		N	0
Tc00.1047053408437.20	hypothetical protein, conserved	0	0	0	N	N		N	0
Tc00.1047053399373.9	retrotransposon hot spot (RHS) protein, putative	0	0	0	N	Y		N	0
Tc00.1047053506475.116	glutaredoxin, putative	0	1	0	N	N		N	0
Tc00.1047053508209.100	10 kDa heat shock protein, putative	0	0	0	N	N		N	0
Tc00.1047053509965.394	amastin, putative	4	4	4	N	Y		N	4
Tc00.1047053507941.150	histone H4, putative	0	0	0	N	N		N	0
Tc00.1047053506789.140	hypothetical protein, conserved	0	0	0	N	N		N	0
Tc00.1047053508741.170	hypothetical protein, conserved	0	0	0	N	N		N	0
Tc00.1047053506195.110	malate dehydrogenase, putative	0	0	0	N	N		N	0
Tc00.1047053506587.70	hypothetical protein, conserved	2	2	2	N	N		N	2
Tc00.1047053506679.100	40S ribosomal protein S18, putative	0	0	0	N	N		N	0
Tc00.1047053506789.240	hypothetical protein, conserved	1	1	3	Y	N		N	1
Tc00.1047053511211.160	heat shock protein 70 (HSP70), putat	0	0	0	N	N		N	0
Tc00.1047053511809.130	40S ribosomal protein S15, putative	0	0	0	N	N		N	0
Tc00.1047053506201.170	hypothetical protein, conserved	0	0	0	N	N		N	0
Tc00.1047053506275.20	hslvu complex proteolytic subunit-lik	0	0	0	N	N		N	0
Tc00.1047053506893.100	hypothetical protein, conserved	0	0	0	N	N		N	0
Tc00.1047053509129.10	hypothetical protein, conserved	0	0	0	N	N		N	0
Tc00.1047053407477.50	cytochrome c oxidase VIII (COX VIII),	1	1	1	N	N		N	1
Tc00.1047053505169.10	null	2	2	2	N	N		N	2
Tc00.1047053506943.50	glyceraldehyde 3-phosphate dehydrogen	0	0	0	N	N		N	0
Tc00.1047053506629.40	hypothetical protein, conserved	0	0	0	Y	N		N	0
Tc00.1047053511071.130	basal body component, putative	0	0	0	N	N		N	0
Tc00.1047053511635.10	histone H2B, putative	0	0	0	N	N		N	0
