# Supplementary material for: Identification of Contractile Vacuole Proteins in Trypanosoma cruzi
Source: PLoS One. 2011 Mar 18;6(3):e18013. doi: 10.1371/journal.pone.0018013 (PMC3060929; doi:10.1371/journal.pone.0018013)
Supplement: Table S5 — Annotated proteins identified by mass spectrometry in enriched CV fractions. This Table lists the known proteins identified in CV fractions according to their potential function. (PDF) [file pone.0018013.s005.pdf]

Table S5 - Annotated proteins identified by mass spectrometry in enriched CV fractions. When present, experimentally determined locations and their respective references are given. Predicted locations, as determined using five protein localization programs (see Additional file 6) are given for comparison. Abbreviations used: S, secreted. C, cytosol. M, mitochondrion. N, nucleus. PM, plasma membrane. G, Golgi complex. ER, endoplasmic reticulum, P, peroxisome. L, lysosome. CYSK, cytoskeleton.

Notes: (*) The assignment of the protein cellular location is based on: a. publications describing the location of the protein in *T cruzi;* b. publications describing the location of conserved pathways in trypanosomatids or in other eukaryotes; c. gene ontology annotation found in GeneBank. d. in silico prediction based in targeting signals. When the location is unknown or uncertain, an **X** is displayed in the table. An asterisk indicates when the location of the protein in *T. cruzi* has been experimentally demonstrated. Genes corresponding to gene products not previously detected in proteomic studies or epimastigotes are in red. Genes whose gene products are studied in this work or are known to be present in the CV of this or other organisms are in green.

PubMed Reference: The references correspond to work in which the gene, protein, or activity of the protein has been described either in *T. cruzi* or in other trypanosomatids. When no reference in trypanosomatids is available, the reference corresponds to the identification of the gene, protein or activity in other species. N/A : references not available in trypanosomatids

Name	Tc #	Protein Accession	Predicted Location	Experimentally Determined Location	Reference
**AMINO ACID AND POLYAMINE METABOLISM**					
histidine ammonia-lyase, putative	Tc00.1047053506247.220	EAN98485		X	Atwood JA 3rd, Weatherly DB, Minning TA, Bundy B, Cavola C, Opperdoes FR, Orlando R, Tarleton RL. The *Trypanosoma cruzi* proteome. *Science.* 2005 Jul 15;309(5733):473-6.
glutamate dehydrogenase, putative	Tc00.1047053509445.39	EAN82723	M	C / M*	P Barderi, O Campetella, A C Frasch, J A Santomé, U Hellman, U Pettersson, and J J Cazzulo. The NADP+-linked glutamate dehydrogenase from *Trypanosoma cruzi*: sequence, genomic organization and expression. *Biochem J.* 1998 March 1; 330(Pt 2): 951-58.
	Tc00.1047053505843.10	EAN84271	M	C / M*	Same as previous
delta-1-pyrroline-5-carboxylate reductase, putative	Tc00.1047053510943.50	EAN95227		X	N/A. Tomenchok, D.M., and Brandiss, M.C. Gene-enzyme relationships in the proline biosynthetic pathway of *Saccharomyces cerevisiae. J Bacteriol*. 1987 Dec;169(12):5364-72.
	Tc00.1047053509351.10	EAN81376		X	N/A. Same as previous
peptide methionine sulfoxide reductase, putative	Tc00.1047053510855.10	EAN83377		X	N/A. Schmidt A, Clayton CE, Krauth-Siegel RL. Silencing of the thioredoxin gene in *Trypanosoma brucei brucei. Mol Biochem Parasitol.* 2002 Nov-Dec;125(1-2):207-10.
**NUCLEOSIDE, NUCLEOTIDE & NUCLEIC ACID METABOLISM**					
inosine-5'-monophosphate dehydrogenase, putative	Tc00.1047053506519.130	EAN91257	C	X	Wilson K, Berens RL, Sifri CD, Ullman B. Amplification of the inosinate dehydrogenase gene in *Trypanosoma brucei gambiense* due to an increase in chromosome copy number. *J Biol Chem.* 1994 Nov 18;269(46):28979-87.
**CARBOHYDRATE METABOLISM**					
glucosamine-6-phosphate isomerase, putative	Tc00.1047053511531.50	EAN86006	C	glycosome	N/A. Natarajan K, Datta A. Molecular cloning and analysis of the NAG1 cDNA coding for glucosamine-6-phosphate deaminase from *Candida albicans. J Biol Chem.* 1993 May 5;268(13):9206-14. // Opperdoes FR, Szikora JP. In silico prediction of the glycosomal enzymes of *Leishmania major* and trypanosomes. *Mol Biochem Parasitol.* 2006 Jun;147(2):193-206.
pyruvate phosphate dikinase, putative	Tc00.1047053510101.140	EAN99374		glycosome	Maldonado RA, Fairlamb AH. Cloning of a pyruvate phosphate dikinase from *Trypanosoma cruzi. Mol Biochem Parasitol.* 2001 Feb;112(2):183-91.
	Tc00.1047053506297.190	EAN98352		glycosome	Same as previous
alkyl-dihydroxyacetone phosphate synthase, putative	Tc00.1047053503815.10	EAN82998		glycosome	Zomer AW, Opperdoes FR, van den Bosch H. Alkyl dihydroxyacetone phosphate synthase in glycosomes of *Trypanosoma brucei. Biochim Biophys Acta.* 1995 Jul 13;1257(2):167-73/ Zomer AW, Michels PA, Opperdoes FR. Molecular characterisation of *Trypanosoma brucei* alkyl dihydroxyacetone-phosphate synthase. *Mol Biochem Parasitol.* 1999 Oct 25;104(1):55-66.
lipophosphoglycan biosynthetic protein, putative (HSP90)	Tc00.1047053506989.190	EAN96800	S	ER	Descoteaux A, Avila HA, Zhang K, Turco SJ, Beverley SM. Leishmania LPG3 encodes a GRP94 homolog required for phosphoglycan synthesis implicated in parasite virulence but not viability. *EMBO J.* 2002 Sep 2;21(17):4458-69.
**LIPID, FATTY ACID AND STEROL METABOLISM**					
sterol C-24 reductase, putative	Tc00.1047053506577.120	EAN95037		X	N/A, although ergosterol is present in *T. cruzi*. Docampo R, Moreno SN, Turrens JF, Katzin AM, Gonzalez-Cappa SM, Stoppani AO. Biochemical and ultrastructural alterations produced by miconazole and econazole in *Trypanosoma cruzi. Mol Biochem Parasitol.* 1981 Jul;3(3):169-80.
3-oxo-5-alpha-steroid 4-dehydrogenase, putative	Tc00.1047053457251.10	EAN81369		X	N/A, in trypanosomatids, although NADPH cytochrome c reductase activities are present. Kuwahara T, White RA Jr, Agosin M. NADPH-cytochrome c reductases of *Trypanosoma cruzi. Biochem Biophys Res Commun.* 1984 Oct 15;124(1):121-4.
acyl-CoA dehydrogenase, putative	Tc00.1047053508827.40	EAN92888	M	X	N/A. Matsubara Y, Kraus JP, Yang-Feng TL, Francke U, Rosenberg LE, Tanaka K. Molecular cloning of cDNAs encoding rat and human medium-chain acyl-CoA dehydrogenase and assignment of the gene to human chromosome 1. *Proc Natl Acad Sci U S A.* 1986 Sep;83(17):6543-7.
	Tc00.1047053509153.120	EAN93479	M	X	Same as previous

enoyl-CoA hydratase/isomerase family protein, putative	Tc00.1047053511529.160	EAN93883		glycosome	Wiemer EA, IJlst L, van Roy J, Wanders RJ, Opperdoes FR. Identification of 2-enoyl coenzyme A hydratase and NADP(+)-dependent 3-hydroxyacyl-CoA dehydrogenase activity in glycosomes of procyclic *Trypanosoma brucei. Mol Biochem Parasitol*. 1996 Nov 12;82(1):107-11.
enoyl-CoA hydratase, mitochondrial precursor, putative	Tc00.1047053508153.130	EAO00193		X	N/A. Kanazawa M, Ohtake A, Abe H, Yamamoto S, Satoh Y, Takayanagi M, Niimi H, Mori M, Hashimoto T.. Molecular cloning and sequence analysis of the cDNA for human mitochondrial short-chain enoyl-CoA hydratase. *Enzyme Protein*. 1993;47(1):9-13.
3-ketoacyl-CoA thiolase, putative	Tc00.1047053509463.30	EAN85395	M	glycosome	Opperdoes FR, Szikora JP. In silico prediction of the glycosomal enzymes of *Leishmania major* and trypanosomes. *Mol Biochem Parasitol*. 2006 Jun;147(2):193-206.
thiolase protein-like protein, putative	Tc00.1047053511389.150	EAN92450	M	glycosome	same as previous
Trifunctional enzyme alpha subunit, mitochondrial precursor-like protein, putative	Tc00.1047053508981.39	EAN84570	M	X	N/A. IJlst L, Ruiter JP, Hoovers JM, Jakobs ME, Wanders RJ. Common missense mutation G1528C in long-chain 3-hydroxyacyl-CoA dehydrogenase deficiency. Characterization and expression of the mutant protein, mutation analysis on genomic DNA and chromosomal localization of the mitochondrial trifunctional protein alpha subunit gene. *J Clin Invest*. 1996 Aug 15;98(4):1028-33.
	Tc00.1047053509701.10	EAN81265		X	Same as previous
**THIOL METABOLISM**					
tryparedoxin peroxidase, putative	Tc00.1047053509499.14	EAN84888	M	C / M	Lopez JA, Carvalho TU, de Souza W, Flohé L, Guerrero SA, Montemartini M, Kalisz HM, Nogoceke E, Singh M, Alves MJ, Colli W. Evidence for a trypanothione-dependent peroxidase system in *Trypanosoma cruzi. Free Radic Biol Med.* 2000 Mar 1;28(5):767-72.
trypanothione/tryparedoxin dependent peroxidase 2, putative	Tc00.1047053503899.119	EAN90957		glycosome/M	Hillebrand H, Schmidt A, Krauth-Siegel RL. A second class of peroxidases linked to the trypanothione metabolism. *J Biol Chem.* 2003 Feb 28;278(9):6809-15.
glutaredoxin, putative	Tc00.1047053506475.116	EAN95349		X	Filser M, Comini MA, Molina-Navarro MM, Dirdjaja N, Herrero E, Krauth-Siegel RL. Cloning, functional analysis, and mitochondrial localization of *Trypanosoma brucei* monothiol glutaredoxin-1. *Biol Chem*. 2008 Jan;389(1):21-32.
**ENERGY METABOLISM**					
aconitase, putative	Tc00.1047053511277.290	EAO00045	M	M	Piacenza L, Irigoín F, Alvarez MN, Peluffo G, Taylor MC, Kelly JM, Wilkinson SR, Radi R. Mitochondrial superoxide radicals mediate programmed cell death in *Trypanosoma cruzi*: cytoprotective action of mitochondrial iron superoxide dismutase overexpression. *Biochem J.* 2007 Apr 15;403(2):323-34.
2-oxoglutarate dehydrogenase subunit, putative	Tc00.1047053503793.10	EAN81565		M	N/A. Repetto B, Tzagoloff A. Structure and regulation of KGD1, the structural gene for yeast alpha-ketoglutarate dehydrogenase. *Mol Cell Biol.* 1989 Jun;9(6):2695-705.
2-oxoglutarate dehydrogenase E1 component, putative	Tc00.1047053506337.70	EAN93176	M	M	same as above
dihydrolipoyl dehydrogenase, putative	Tc00.1047053507089.270	EAN96941		M	Schöneck R, Billaut-Mulot O, Numrich P, Ouaissi MA, Krauth-Siegel RL. Cloning, sequencing and functional expression of dihydrolipoamide dehydrogenase from the human pathogen *Trypanosoma cruzi. Eur J Biochem.* 1997 Feb 1;243(3):739-47. // /// Portela MP, Stopopani AO. Lipoamide dehydrogenase from *Trypanosoma cruzi:* some properties and cellular localization. *Biochem Int.* 1991 May;24(1):147-55.
succinate dehydrogenase flavoprotein, putative	Tc00.1047053504949.30	EAN82006		M	Christmas PB, Turrens JF. Separation of NADH-fumarate reductase and succinate dehydrogenase activities in *Trypanosoma cruzi. FEMS Microbiol Lett.* 2000 Feb 15;183(2):225-8.
	Tc00.1047053511909.40	EAN87573	M	M	Same as previous
mitochondrial malate dehydrogenase, putative	Tc00.1047053506195.110	EAN97253		M*	Cannata JJ, Cazzulo JJ. Glycosomal and mitochondrial malate dehydrogenases in epimastigotes of *Trypanosoma cruzi. Mol Biochem Parasitol.* 1984 Apr;11:37-49.
NADH-dependent fumarate reductase, putative	Tc00.1047053510215.10	EAN81600		glycosome/ M	Besteiro S, Biran M, Biteau N, Coustou V, Baltz T, Canioni P, Bringaud F. Succinate secreted by *Trypanosoma brucei* is produced by a novel and unique glycosomal enzyme, NADH-dependent fumarate reductase. *J Biol Chem*. 2002 Oct 11;277(41):38001-12. // Coustou V, Besteiro S, Rivière L, Biran M, Biteau N, Franconi JM, Boshart M, Baltz T, Bringaud F. A mitochondrial NADH-dependent fumarate reductase involved in the production of succinate excreted by procyclic *Trypanosoma brucei. J Biol Chem.* 2005 Apr 29;280(17):16559-70.
	Tc00.1047053508535.10	EAN85469.1		glycosome/ M	Same as previous
	Tc00.1047053503849.60	EAN88381		glycosome/ M	Same as previous
glycerol-3-phosphate dehydrogenase, putative	Tc00.1047053511151.90	EAN90136	M	glycosome	Concepcion JL, Acosta H, Quiñones W, Dubourdieu M. A alpha-glycerophosphate dehydrogenase is present in *Trypanosoma cruzi glycosomes. Mem Inst Oswaldo Cruz*. 2001 Jul;96(5):697-701.
carnitine/choline acetyltransferase, putative	Tc00.1047053509999.90	EAN93060	M	X	N/A. Elgersma Y, van Roermund CW, Wanders RJ, Tabak HF. Peroxisomal and mitochondrial carnitine acetyltransferases of Saccharomyces cerevisiae are encoded by a single gene. *EMBO J.* 1995 Jul 17;14(14):3472-9.
**ELECTRON CARRIERS AND TRANSPORT**					
NADH dehydrogenase, putative	Tc00.1047053506839.70	EAN90346	M	M	Fang J, Beattie DS. Identification of a gene encoding a 54 kDa alternative NADH dehydrogenase in *Trypanosoma brucei. Mol Biochem Parasitol.* 2003 Mar;127(1):73-7.
cytochrome c, putative	Tc00.1047053506949.50	EAN87335	M	M	Torri AF, Hajduk SL. Posttranscriptional regulation of cytochrome c expression during the developmental cycle of *Trypanosoma brucei. Mol Cell Biol*. 1988 Nov;8(11):4625-33.
cytochrome c oxidase subunit V, putative	Tc00.1047053508503.20	EAN86560		M	Mayho M, Fenn K, Craddy P, Crosthwaite S, Matthews K. Post-transcriptional control of nuclear-encoded cytochrome oxidase subunits in *Trypanosoma brucei*: evidence for genome-wide conservation of life-cycle stage-specific regulatory elements. *Nucleic Acids Res.* 2006;34(18):5312-24.
cytochrome c oxidase, subunit VII, putative	Tc00.1047053503769.40	EAN84925		M	Same as previous
cytochrome c oxidase, subunit VIII (COX VIII), putative	Tc00.1047053407477.50	EAN83385		M	Same as previous
cytochrome c oxidase subunit 10, putative	Tc00.1047053455721.9	EAN81337		M	Same as previous
**ATP-PROTON MOTIF FORCE INTERCONVERSION**					

ATP synthase F1 subunit gamma protein, putative	Tc00.1047053504069.80	EAN89661		M	Cataldi de Flombaum MA, Stoppani AO. Influence of efrapeptin, aurovertin and citreoviridin on the mitochondrial adenosine triphosphatase from *Trypanosoma cruzi. Mol Biochem Parasitol.* 1981 Jul;3(3):143-55. // Brown B SV, Stanislawski A, Perry QL, Williams N. Cloning and characterization of the subunits comprising the catalytic core of the *Trypanosoma brucei* mitochondrial ATP synthase. *Mol Biochem Parasitol.* 2001 Apr 6;113(2):289-301.
ATP synthase, alpha chain, mitochondrial precursor, putative	Tc00.1047053510395.10	EAN81916	M	M	Same as previous
ATPase beta subunit, putative	Tc00.1047053509233.180	EAN95983	M	M	Same as previous
vacuolar ATP synthase, subunit B, putative	Tc00.1047053506025.50	EAN86474		acidocalcisome/CV, inconclusive	Similar to subunit B of the vacuolar H+-ATPase.// Zhong L, Lu HG, Moreno SN, Docampo R. Ca2+ content and expression of an acidocalcisomal calcium pump are elevated in intracellular forms of *Trypanosoma cruzi. Mol Cell Biol.* 1998 Apr;18(4):2309-23. Rohloff P, Montalvetti A, Docampo R. Acidocalcisomes and the contractile vacuole complex are involved in osmoregulation in *Trypanosoma cruzi. J Biol Chem.* 2004 Dec 10;279(50):52270-81
vacuolar proton translocating ATPase, subunit a, putative	Tc00.1047053509601.70	EAN92926		acidocalcisome/CV, inconclusive	Similar to subunit a of the vacuolar H+-ATPase. Same as previous
vacuolar-type proton translocating pyrophosphatase 1, putative	Tc00.1047053510773.20	EAN91609		acidocalcisome/CV*	Scott DA, de Souza W, Benchimol M, Zhong L, Lu HG, Moreno SN, Docampo R.Presence of a plant-like proton-pumping pyrophosphatase in acidocalcisomes of *Trypanosoma cruzi. J Biol Chem.* 1998 Aug 21;273(34):22151-8. // Hill JE, Scott DA, Luo S, Docampo R. Cloning and functional expression of a gene encoding a vacuolar-type proton-translocating pyrophosphatase from *Trypanosoma cruzi. Biochem J*. 2000 Oct 1;351(Pt 1):281-8.
**GLYCOLYSIS / GLUCONEOGENESIS**					
glyceraldehyde 3-phosphate dehydrogenase, putative	Tc00.1047053506943.50	EAN90286		glycosome	Kendall G, Wilderspin AF, Ashall F, Miles MA, Kelly JM. *Trypanosoma cruzi* glycosomal glyceraldehyde-3-phosphate dehydrogenase does not conform to the 'hotspot' topogenic signal model. *EMBO J.* 1990 Sep;9(9):2751-8.
triosephosphate isomerase, putative	Tc00.1047053508647.200	EAN96402		glycosome	Ostoa-Saloma P, Garza-Ramos G, Ramírez J, Becker I, Berzunza M, Landa A, Gómez-Puyou A, Tuena de Gómez-Puyou M, Pérez-Montfort R. Cloning, expression, purification and characterization of triosephosphate isomerase from *Trypanosoma cruzi. Eur J Biochem.* 1997 Mar 15;244(3):700-5.
glycosomal phosphoenolpyruvate carboxykinase, putative	Tc00.1047053507547.90	EAN88964		glycosome*	Linss J, Goldenberg S, Urbina JA, Amzel LM. Cloning and characterization of the gene encoding ATP-dependent phospho-enol-pyruvate carboxykinase in *Trypanosoma cruzi*: comparison of primary and predicted secondary structure with host GTP-dependent enzyme. *Gene*. 1993 Dec 22;136(1-2):69-77.
**CELLULAR STRUCTURE, MOTILITY, AND ORGANIZATION**					
beta tubulin, putative	Tc00.1047053506563.40	EAN94839	C	CYSK/ flagellar and sub-pellicular microtubules*	De Lima AR, Medina R, Uzcanga GL, Noris Suárez K, Contreras VT, Navarro MC, Arteaga R, Bubis J. Tight binding between a pool of the heterodimeric alpha/beta tubulin and a protein kinase CK2 in *Trypanosoma cruzi* epimastigotes. *Parasitology*. 2006 Apr;132(Pt 4):511-23. .// Vaughan S, Attwood T, Navarro M, Scott V, McKean P, Gull K. New tubulins in protozoal parasites. *Curr Biol. 2000* Apr 6;10(7):R258-9.
alpha tubulin, putative	Tc00.1047053411235.9	EAN81053		CYSK/ flagellar and sub-pellicular microtubules*	same as previous /// Souto-Padron T, Cunha e Silva NL, de Souza W. Acetylated alpha-tubulin in *Trypanosoma cruzi:* immunocytochemical localization. *Mem Inst Oswaldo Cruz*. 1993 88(4):517-28.
myosin heavy chain, putative	Tc00.1047053506491.20	EAN86336		CYSK	Doberstein SK, Baines IC, Wiegand G, Korn ED, Pollard TD. Inhibition of contractile vacuole function in vivo by antibodies against myosin-I. *Nature*. 1993 Oct 28;365(6449):841-3.// Oliveira MF, Bijovsky AT, Carvalho TU, de Souza W, Alves MJ, Colli W. A monoclonal antibody to *Trypanosoma cruzi* trypomastigotes recognizes a myosin tail epitope. *Parasitol Res*. 2001 Dec;87(12):1043-9.
myosin IB heavy chain, putative (Myosin V-2)	Tc00.1047053507739.110	EAN89650		CYSK	Same as previous
cytoskeleton-associated protein CAP5.5, putative	Tc00.1047053509237.130	EAN92788		CYSK	Hertz-Fowler C, Ersfeld K, Gull K. CAP5.5, a life-cycle-regulated, cytoskeleton-associated protein is a member of a novel family of calpain-related proteins in *Trypanosoma brucei*. *Mol Biochem Parasitol.* 2001 Aug;116(1):25-34. Erratum in: Mol Biochem Parasitol 2001 Sep 28;117(1):119.
	Tc00.1047053508555.60	EAN87963		CYSK	Same as previous
dynein heavy chain, putative	Tc00.1047053509585.10	EAN84848	C	CYSK	Branche C, Kohl L, Toutirais G, Buisson J, Cosson J, Bastin P. Conserved and specific functions of axoneme components in trypanosome motility. *J Cell Sci*. 2006 Aug 15;119(Pt 16):3443-55.
	Tc00.1047053508815.179	EAN93755		CYSK	Same as previous
	Tc00.1047053508275.9	EAN82251	C	CYSK	Same as previous
	Tc00.1047053433273.10	EAN80780		CYSK	Same as previous
myosin heavy chain, putative (Myosin V-1)	Tc00.1047053511527.70	EAN87803		CYSK	Doberstein SK, Baines IC, Wiegand G, Korn ED, Pollard TD. Inhibition of contractile vacuole function in vivo by antibodies against myosin-I. *Nature*. 1993 Oct 28;365(6449):841-3.// Oliveira MF, Bijovsky AT, Carvalho TU, de Souza W, Alves MJ, Colli W. A monoclonal antibody to *Trypanosoma cruzi* trypomastigotes recognizes a myosin tail epitope. *Parasitol Res.* 2001 Dec;87(12):1043-9.
flagellar radial spoke component, putative	Tc00.1047053504089.50	EAN90808		flagellum	Ralston KS, Lerner AG, Diener DR, Hill KL. Flagellar motility contributes to cytokinesis in *Trypanosoma brucei* and is modulated by an evolutionarily conserved dynein regulatory system. *Eukaryot Cell*. 2006 Apr;5(4):696-711.
flagellum-adhesion glycoprotein, putative	Tc00.1047053509561.20	EAN90705	S	flagellum	Cooper R, de Jesus AR, Cross GA. Deletion of an immunodominant *Trypanosoma cruzi* surface glycoprotein disrupts flagellum-cell adhesion. *J Cell Biol.* 1993 Jul;122(1):149-56.
69 kDa paraflagellar rod protein, putative	Tc00.1047053511215.119	EAN92318		flagellum*	Beard, C. A,, Sahorio, J. L., Tewari, D., Krieglstein, K. G., Henschen,A. H., andManning, J. E. Evidence for two distinct major protein components, PAR 1 and PAR 2, in the paraflagellar rod of *Trypanosoma cruzi*. Complete nucleotide sequence of PAR. *J. Biol. Chem.* 1992 267, 21656-21662 // Fouts DL, Stryker GA, Gorski KS, Miller MJ, Nguyen TV, Wrightsman RA, Manning JE. Evidence for four distinct major protein components in the paraflagellar rod of *Trypanosoma cruzi. J Biol Chem.* 1998 Aug 21;273(34):21846-55.
paraflagellar rod protein 3, putative	Tc00.1047053509617.20	EAN87979	C	flagellum*	Same as previous

paraflagellar rod component Par4, putative	Tc00.1047053510353.30	EAN83974		flagellum*	Same as previous
paraflagellar rod component, putative	Tc00.1047053506755.20	EAN96891		flagellum*	Same as previous
kinetoplastid membrane protein KMP-11	Tc00.1047053508413.68	EAN87014		flagellum/ flagellar pocket/ CYSK*	M C Thomas , J L García-Pérez , C Alonso , M C López. Molecular characterization of KMP11 from *Trypanosoma cruzi*: a cytoskeleton-associated protein regulated at the translational level. *DNA Cell Biol*. 2000 19 (1):47-57.
Basal body component, putative	Tc00.1047053511071.130	EAN92157		basal body, N, flagellum	Dilbeck V, Berberof M, Van Cauwenberge A, Alexandre H, Pays E. Characterization of a coiled coil protein present in the basal body of *Trypanosoma brucei. J Cell Sci.* 1999 Dec;112 (Pt 24):4687-94.
**CELL ADHESION / SURFACE**					
amastin, putative	Tc00.1047053509051.20	EAN84309		PM	Teixeira SM, Russell DG, Kirchhoff LV, Donelson JE. differentially expressed gene family encoding amastin, a surface protein of *Trypanosoma cruzi* amastigotes. *J Biol Chem*. 1994 Aug 12;269(32):20509-16.
	Tc00.1047053509965.394	EAN98408		PM	same as previous
extracellular receptor, putative	Tc00.1047053506503.140	EAN95091		PM	N/A. Contains two pfam01094 (receptor family binding region) found in a wide range of receptors and bacterial amino acid binding proteins
**REPLICATION, TRANSCRIPTION, DNA REPAIR, AND DNA BINDING**					
histone H4, putative	Tc00.1047053507941.150	EAN90974.1		N*	Alsford S, Horn D. Trypanosomatid histones. Mol Microbiol. 2004 Jul;53(2):365-72. /// da Cunha JP, Nakayasu ES, de Almeida IC, Schenkman S. Post-translational modifications of *Trypanosoma cruzi* histone H4. *Mol Biochem Parasitol.* 2006 Dec;150(2):268-77.
histone H2B, putative	Tc00.1047053511635.10	EAN83533		N*	Alsford S, Horn D. Trypanosomatid histones. Mol Microbiol. 2004 Jul;53(2):365-72. /// da Cunha JP, Nakayasu ES, de Almeida IC, Schenkman S. Post-translational modifications of *Trypanosoma cruzi* histone H4. *Mol Biochem Parasitol.* 2006 Dec;150(2):268-77. /// Toro GC, Wernstedt C, Hellman U, Galanti N. Presence of histone H2B in *Trypanosoma cruzi* chromatin. *Biol Res.* 1993;26(1-2):41-6.
kinetoplast DNA-associated protein, putative	Tc00.1047053511029.20	EAN81680	M	kinetoplast*	Zavala-Castro JE, Acosta-Viana K, Guzmán-Marín E, Rosado-Barrera ME, Rosales-Encina JL. Stage specific kinetoplast DNA-binding proteins in *Trypanosoma cruzi. Acta Trop.* 2000 Sep 18;76(2):139-46. /// Zavala-Castro JE, Acosta-Viana K, Baylon-Pacheco L, González-Robles A, Guzmán-Marín E, Rosales-Encina JL. Kinetoplast DNA-binding protein profile in the epimastigote form of *Trypanosoma cruzi. Arch Med Res.* 2002 May-Jun;33(3):250-6.
	Tc00.1047053508719.40	EAN87060		kinetoplast*	Same as previous
	Tc00.1047053508719.60	EAN87062		kinetoplast*	Same as previous
mitochondrial DNA topoisomerase II, putative	Tc00.1047053506445.60	EAN91964		M	Melendy, T., Sheline, C., and Ray, D.S. Localization of a type II DNA topoisomerase to two sites at the periphery of the kinetoplast DNA of *Crithidia fasciculata. Cell* 55:1083-1088, 1988.
Mitochondrial oligo_U binding protein TBRGG1, putative	Tc00.1047053507927.20	EAN87685		M	Vanhamme L, Perez-Morga D, Marchal C, Speijer D, Lambert L, Geuskens M, Alexandre S, Ismaïli N, Göringer U, Benne R, Pays E. *Trypanosoma brucei* TBRGG1, a mitochondrial oligo(U)-binding protein that co-localizes with an in vitro RNA editing activity. *J Biol Chem*. 1998 Aug 21;273(34):21825-33.
Ribonuclease mar1, putative	Tc00.1047053505945.20	EAN87195		N	Caruthers J, Zucker F, Worthey E, Myler PJ, Buckner F, Van Voorhuis W, Mehlin C, Boni E, Feist T, Luft J, Gulde S, Lauricella A, Kaluzhniy O, Anderson L, Le Trong I, Holmes MA, Earnest T, Soltis M, Hodgson KO, Hol WG, Merritt EA. Crystal structures and proposed structural/functional classification of three protozoan proteins from the isochorismatase superfamily. *Protein Sci.* 2005 Nov;14(11):2887-94.
**TRANSCRIPTION, RNA PROCESSING, AND RNA BINDING**					
Mitochondrial RNA-binding protein 2, putative	Tc00.1047053506755.260	EAN96915	M	M	N/A
Mitochondrial RNA-binding protein	Tc00.1047053506933.60	EAN90161		M	N/A
p22 protein precursor, putative	Tc00.1047053509053.70	EAN91460	M	M	Hayman ML, Miller MM, Chandler DM, Goulah CC, Read LK. The trypanosome homolog of human p32 interacts with RBP16 and stimulates its gRNA binding activity. *Nucleic Acids Res*. 2001 Dec 15;29(24):5216-25.
MP99, putative	Tc00.1047053508153.1100	EAO00253		M	Panigrahi AK, Gygi SP, Ernst NL, Igo RP Jr, Palazzo SS, Schnaufer A, Weston DS, Carmean N, Salavati R, Aebersold R, Stuart KD. Association of two novel proteins, TbMP52 and TbMP48, with the *Trypanosoma brucei* RNA editing complex. *Mol Cell Biol.* 2001 Jan;21(2):380-9.
Isoleucyl-tRNA synthetase, putative	Tc00.1047053509797.40	EAN86703		X	N/A. Ribas de Pouplana L, Geslain R. Not just because it is there: aminoacyl-tRNA synthetases gain control of the cell. *Mol Cell.* 2008 Apr 11;30(1):3-4.
Leucyl-tRNA synthetase, putative	Tc00.1047053509679.9	EAN81004		X	N/A. Same as previous
**TRANSLATION AND PROTEIN SYNTHESIS**					
ribosomal protein S19, putative	Tc00.1047053504013.100	EAN88197		ER	N/A. Suzuki K, Olvera J, Wool IG. The primary structure of rat ribosomal protein S19. *Biochimie.* 1990 Apr;72(4):299-302.
40S ribosomal protein S27, putative	Tc00.1047053506963.14	EAN89730		ER	N/A. Chan YL, Suzuki K, Olvera J, Wool IG. Zinc finger-like motifs in rat ribosomal proteins S27 and S29. *Nucleic Acids Res.* 1993 Feb 11;21(3):649-55.
ribosomal protein S29, putative	Tc00.1047053506025.14	EAN86477		ER	N/A. same as previous
40S ribosomal protein S15, putative	Tc00.1047053511809.130	EAN91806		C	Cassola C, De Gaudenzi JG, Frasch C. Recruitment of mRNAs to cytoplasmic ribonucleoprotein granules in trypanosomes. *Mol Microbiol.* 2007 Aug; 65(3):655-670.

40S ribosomal protein S18, putative	Tc00.1047053506679.100	EAN98186			Same as previous
60S ribosomal protein L12, putative	Tc00.1047053504037.30	EAN82434		ER	N/A. Suzuki K, Olvera J, Wool IG. The primary structure of rat ribosomal protein L12. *Biochem Biophys Res Commun.* 1990 Oct 15;172(1):35-41.
60S ribosomal protein L22, putative	Tc00.1047053504147.120	EAN97983		ER	N/A. Chan YL, Wool IG. The primary structure of rat ribosomal protein L22. *Biochim Biophys Acta*. 1995 Jan 2;1260(1):113-5.
methyltransferase, putative (Golvesin 1)	Tc00.1047053509805.40	EAN98089		X	N/A. Kressler D, Rojo M, Linder P, Cruz J. Spb1p is a putative methyltransferase required for 60S ribosomal subunit biogenesis in *Saccharomyces cerevisiae. Nucleic Acids Res*. 1999 Dec 1;27(23):4598-608.
**TRANSLATION FACTORS**					
elongation factor 1-alpha (EF-1-alpha), putative	Tc00.1047053510119.20	EAN84978		C	Billaut-Mulot O, Fernandez-Gomez R, Loyens M, Ouaissi A. *Trypanosoma cruzi* elongation factor 1-alpha: nuclear localization in parasites undergoing apoptosis. *Gene*. 1996 Sep 26;174(1):19-26.
mitochondrial elongation factor G, putative	Tc00.1047053506583.60	EAN84717		M	N/A. Hammarsund M, Wilson W, Corcoran M, Merup M, Einhorn S, Grandér D, Sangfelt O. Identification and characterization of two novel human mitochondrial elongation factor genes, hEFG2 and hEFG1, phylogenetically conserved through evolution. *Hum Genet.* 2001 Nov;109(5):542-50.
**PROTEIN FOLDING AND STABILIZATION**					
10 kDa heat shock protein, putative	Tc00.1047053508209.120	EAN91214	M	Several compartments	Zamora-Veyl FB, Kroemer M, Zander D, Clos J. Stage-specific expression of the mitochondrial co-chaperonin of *Leishmania donovani*, CPN10. *Kinetoplastid Biol Dis*. 2005 Apr 29;4(1):3.
	Tc00.1047053508209.100	EAN91214		Several compartments	same as previous
Heat shock protein 20, putative	Tc00.1047053508153.270	EAO00223		X	N/A. Shimada T, Kondo M, Hara-Nishimura I, Nishimura M. Chloroplasts have a novel Cpn10 in addition to Cpn20 as co-chaperonins in *Arabidopsis thaliana. J Biol Chem*. 2001 Aug 10;276(32):29688-94.
chaperonin HSP60, mitochondrial precursor	Tc00.1047053507641.290	EAN97657	M	X	Sullivan MA, Olson CL, Winquist AG, Engman DM. Expression and localization of *Trypanosoma cruzi* hsp60. *Mol Biochem Parasitol*. 1994 Dec;68(2):197-208.
heat shock protein 70 (HSP70), putative	Tc00.1047053511211.160	EAN95886		X	de Marval MG, Souto-Padron T, Gottesdiener K, Silva R, van der Ploeg LH, Rondinelli E. Heat shock proteins in *Trypanosoma cruzi*: identification and localization of HSP70 and HSP60 proteins and structure of HSP60 genes (brief report). *Biol Res.* 1993;26(1-2):313-4.
	Tc00.1047053510155.70	EAN98261		X	Same as previous
heat shock 70 kDa protein, mitochondrial precursor, putative	Tc00.1047053507029.30	EAN84370	M	X	Same as previous
heat shock protein 85, putative	Tc00.1047053507713.30	EAN82629	C	Several compartments	Muhich ML, Hsu MP, Boothroyd JC. Heat-shock disruption of trans-splicing in trypanosomes: effect on Hsp70, Hsp85 and tubulin mRNA synthesis. *Gene*. 1989 Oct 15;82(1):169-75
heat shock protein, putative	Tc00.1047053504153.310	EAN99073	M	X	Same as previous
ATP-dependent Clp protease subunit, heat shock protein 78, putative	Tc00.1047053508737.100	EAN95823	M	X	Li Z, Lindsay ME, Motyka SA, Englund PT, Wang CC. Identification of a bacterial-like HslVU protease in the mitochondria of *Trypanosoma brucei* and its role in mitochondrial DNA replication. *PLoS Pathog*. 2008 Apr 18;4(4):e1000048.
chaperone DnaJ protein, putative	Tc00.1047053503843.40	EAN84765		X	Tibbetts RS, Jensen JL, Olson CL, Wang FD, Engman DM. The DnaJ family of protein chaperones in *Trypanosoma cruzi. Mol Biochem Parasitol.* 1998 Mar 15;91(2):319-26.
glucose-regulated protein 78, putative (BiP)	Tc00.1047053506585.40	EAN87966	S	ER	Tibbetts RS, Kim IY, Olson CL, Barthel LM, Sullivan MA, Winquist AG, Miller SD, Engman DM. Molecular cloning and characterization of the 78-kilodalton glucose-regulated protein of *Trypanosoma cruzi. Infect Immun.* 1994 Jun;62(6):2499-507.
co-chaperone GrpE, putative	Tc00.1047053509045.20	EAN81815	M	M	N/A. Moro F, Taneva SG, Velázquez-Campoy A, Muga A. GrpE N-terminal domain contributes to the interaction with Dnak and modulates the dynamics of the chaperone substrate binding domain. *J Mol Biol.* 2007 Dec 7;374(4):1054-64.
Cyclophilin, putative	Tc00.1047053509215.40	EAN86719	S	X	Búa J, Fichera LE, Fuchs AG, Potenza M, Dubin M, Wenger RO, Moretti G, Scabone CM, Ruiz AM. Anti-*Trypanosoma cruzi* effects of cyclosporin A derivatives: possible role of a P-glycoprotein and parasite cyclophilins. *Parasitology.* 2008 Feb;135(2):217-28.
**PROTEOLYSIS**					
glutamamyl carboxypeptidase, putative	Tc00.1047053510837.20	EAN82710		X	N/A. Silink M, Reddel R, Bethel M, Rowe PB. Gamma-glutamyl hydrolase conjugase. Purification and properties of the bovine hepatic enzyme. *J Biol Chem.* 1975 Aug 10;250(15):5982-94.
	Tc00.1047053507689.30	EAN85497		X	Same as previous
carboxypeptidase, putative	Tc00.1047053504153.160	EAN99058		L	Parussini F, García M, Mucci J, Agüero F, Sánchez D, Hellman U, Aslund L, Cazzulo JJ. Characterization of a lysosomal serine carboxypeptidase from *Trypanosoma cruzi. Mol Biochem Parasitol.* 2003 Sep;131(1):11-23.
calpain cysteine peptidase, putative	Tc00.1047053511441.10	EAN83138		reservosome, PM*	Bontempi E, Martinez J, Cazzulo JJ. Subcellular localization of a cysteine proteinase from *Trypanosoma cruzi. Mol Biochem Parasitol.* 1989 Feb;33(1):43-7.
calpain-like cysteine peptidase, putative	Tc00.1047053506983.39	EAN85448		reservosome, PM*	Same as previous
	Tc00.1047053508999.260			reservosome, PM*	Same as previous
	Tc00.1047053506563.79			reservosome, PM*	Same as previous
	Tc00.1047053506563.170			reservosome, PM*	Same as previous

	Tc00.1047053508999.250			reservosome, PM*	Same as previous
ATP-dependent zinc metallopeptidase, putative	Tc00.1047053506223.80	EAN91087	M	X	N/A. Gakh O, Cavadini P, Isaya G. Mitochondrial processing peptidases. *Biochim Biophys Acta*. 2002 Sep 2;1592(1):63-77.
	Tc00.1047053511277.170	EAO00034		X	Same as previous
hslvu complex proteolytic subunit-like, putative	Tc00.1047053506275.20	EAN88322		M	Li Z, Lindsay ME, Motyka SA, Englund PT, Wang CC. Identification of a bacterial-like HslVU protease in the mitochondria of *Trypanosoma brucei* and its role in mitochondrial DNA replication. *PLoS Pathog*. 2008 Apr 18;4(4):e1000048. /// Panigrahi AKY, Ogata A, Zikova A, Anupama RA, Dalley N, Acestor PJ, Myler and Stuart KD. A comprehensive analysis of *Trypanosoma brucei* mitochondrial proteome. 2009. *Proteomics* 9(2): 434-50.
**TRANSPORT PROTEINS - INTRACELLULAR TRAFFIC**					
P-type H+-ATPase, putative	Tc00.1047053505763.19	EAN82405	PM	PM	Luo S, Scott DA, Docampo R. *Trypanosoma cruzi* H+-ATPase 1 (TcHA1) and 2 (TcHA2) genes complement yeast mutants defective in H+ pumps and encode plasma membrane P-type H+-ATPases with different enzymatic properties. *J Biol Chem.* 2002 Nov 15;277(46):44497-506.
calcium channel protein, putative (TPC)	Tc00.1047053504105.130	EAN97848	PM	PM*	Ruben L, Akins CD, Haghighat NG, Xue L. Calcium influx in *Trypanosoma brucei* can be induced by amphiphilic peptides and amines. *Mol Biochem Parasitol.* 1996 Oct 30;81(2):191-200.
small G-protein, putative (Rab32)	Tc00.1047053506289.80	EAN96691		X	Same as previous
small G-protein, putative (Rab23)	Tc00.1047053506355.60	EAN92973	C	X	Same as previous
ABC transporter, putative	Tc00.1047053510149.80	EAN92058		X	Dallagiovanna B, Gamarro F, Castanys S. Molecular characterization of a P-glycoprotein-related tcpgp2 gene in *Trypanosoma cruzi. Mol Biochem Parasitol.* 1996 Jan;75(2):145-57.
	Tc00.1047053506249.70	EAN84815		X	Same as previous
	Tc00.1047053507093.260	EAN98703	M	X	Same as previous
	Tc00.1047053506579.10	EAN89676		X	Same as previous
hexose transporter, putative	Tc00.1047053506355.10	EAN92970	PM	X	Tetaud E, Bringaud F, Chabas S, Barrett MP, Baltz T. Characterization of glucose transport and cloning of a hexose transporter gene in *Trypanosoma cruzi. Proc Natl Acad Sci U S A.* 1994 Aug 16;91(17):8278-82. /// Tetaud E, Barrett MP, Bringaud F, Baltz T. Kinetoplastid glucose transporters. *Biochem J.* 1997 Aug 1;325 (Pt 3):569-80.
nucleobase transporter, putative	Tc00.1047053506445.110	EAN91969		PM	Sanchez MA, Tryon R, Green J, Boor I, Landfear SM. Six related nucleoside/nucleobase transporters from *Trypanosoma brucei* exhibit distinct biochemical functions. *J Biol Chem.* 2002 Jun 14;277(24):21499-504.
folate/pteridine transporter, putative	Tc00.1047053511575.130	EAN91522		PM	Klaus SM, Kunji ER, Bozzo GG, Noiriel A, de la Garza RD, Basset GJ, Ravanel S, Rébeillé F, Gregory JF 3rd, Hanson AD. Higher plant plastids and cyanobacteria have folate carriers related to those of trypanosomatids. *J Biol Chem.* 2005 Nov 18;280(46):38457-63.
transitional endoplasmic reticulum ATPase, putative	Tc00.1047053509733.170	EAN96827	C	ER	N/A. Zhang L, Ashendel CL, Becker GW, Morré DJ. Isolation and characterization of the principal ATPase associated with transitional endoplasmic reticulum of rat liver. *J Cell Biol.* 1994 Dec;127(6 Pt 2):1871-83.
cation transporter, putative	Tc00.1047053508699.130	EAN90805		X	Iizumi K, Mikami Y, Hashimoto M, Nara T, Hara Y, Aoki T. Molecular cloning and characterization of ouabain-insensitive Na(+)-ATPase in the parasitic protist, *Trypanosoma cruzi. Biochim Biophys Acta*. 2006 Jun;1758(6):738-46.
mitochondrial phosphate transporter, putative	Tc00.1047053509551.30	EAN92689		M	N/A. Ferreira GC, Pratt RD, Pedersen PL. Energy-linked anion transport. Cloning, sequencing, and characterization of a full length cDNA encoding the rat liver mitochondrial proton/phosphate symporter. *J Biol Chem.* 1989 Sep 15;264(26):15628-33.
mitochondrial carrier protein, putative	Tc00.1047053504125.50	EAN91369		M	Colasante C, Alibu VP, Kirchberger S, Tjaden J, Clayton C, Voncken F. Characterization and developmentally regulated localization of the mitochondrial carrier protein homologue MCP6 from *Trypanosoma brucei. Eukaryot Cell.* 2006 Aug;5(8):1194-205.
Gim5A protein, putative	Tc00.1047053507009.10	EAN90052		glycosome	Maier A, Lorenz P, Voncken F, Clayton C. An essential dimeric membrane protein of trypanosome glycosomes. *Mol Microbiol.* 2001 Mar;39(6):1443-51.
ADP,ATP carrier protein 1, mitochondrial precursor, putative	Tc00.1047053506211.160	EAN97607		M	Opperdoes FR, Rijke DD, Borst P. Reactions involved in energy transfer in trypanosomes-I. Characterization of the mitochondrial adenine nucleotide translocator and the ATPase of *Crithidia luciliae. Comp Biochem Physiol B.* 1976;54(1):7-12. /// Colasante C, Alibu VP, Kirchberger S, Tjaden J, Clayton C, Voncken F. Characterization and developmentally regulated localization of the mitochondrial carrier protein homologue MCP6 from *Trypanosoma brucei. Eukaryot Cell*. 2006 Aug;5(8):1194-205.
	Tc00.1047053511289.70	EAN90413		M	Same as previous
ADP/ATP translocase, putative	Tc00.1047053506657.40	EAN87278		M	Same as previous
clathrin coat assembly protein, putative (AP180)	Tc00.1047053503449.30	EAN83025	C	X	Corrêa JR, Atella GC, Menna-Barreto RS, Soares MJ. Clathrin in *Trypanosoma cruzi:* in silico gene identification, isolation, and localization of protein expression sites. *J Eukaryot Microbiol*. 2007 May-Jun;54(3):297-302.
clathrin heavy chain, putative	Tc00.1047053506167.50	EAN87927	C	X	Same as previous
coatomer gamma subunit, putative	Tc00.1047053511211.20	EAN95873	G	C	Maier AG, Schulreich S, Bremser M, Clayton C. Binding of coatomer by the PEX11 C-terminus is not required for function. *FEBS Lett.* 2000 Nov 3;484(2):82-6.

peroxin 14, putative	Tc00.1047053504069.50	EAN89658		glycosome	Flaspohler JA, Rickoll WL, Beverley SM, Parsons M. Functional identification of a Leishmania gene related to the peroxin 2 gene reveals common ancestry of glycosomes and peroxisomes. *Mol Cell Biol*. 1997 Mar;17(3):1093-101.
mitochondrial import inner membrane translocase subunit Tim17, putative	Tc00.1047053506755.250	EAN96914	M	M	Singha UK, Peprah E, Williams S, Walker R, Saha L, Chaudhuri M. Characterization of the mitochondrial inner membrane protein translocator Tim17 from *Trypanosoma brucei. Mol Biochem Parasitol.* 2008 May;159(1):30-43.
vacuolar protein sorting protein 18, putative	Tc00.1047053506401.70	EAN99410	C	X	Lu S, Suzuki T, Iizuka N, Ohshima S, Yabu Y, Suzuki M, Wen L, Ohta N. *Trypanosoma brucei* vacuolar protein sorting 41 (VPS41) is required for intracellular iron utilization and maintenance of normal cellular morphology. *Parasitology.* 2007 Oct;134(Pt 11):1639-47.
vacuolar protein sorting complex subunit, putative	Tc00.1047053511269.60	EAN87604		X	Same as previous
SNF-7-like protein, putative	Tc00.1047053506957.110	EAN93195		X	N/A. Tu J, Vallier LG Carlson M. Molecular and genetic analysis of the SNF7 gene in *Saccharomyces cerevisiae. Genetics* 135 (1), 17-23 (1993)
epsilon-adaptin, putative	Tc00.1047053511751.200	EAN96655		X	Fralish BH, Tarleton RL. Genetic immunization with LYT1 or a pool of trans-sialidase genes protects mice from lethal *Trypanosoma cruz*i infection. Vaccine. 2003 Jun 20;21(21-22):3070-80// Allen CL, Liao D, Chung WL, Field MC. Dileucine signal-dependent and AP-1-independent targeting of a lysosomal glycoprotein in *Trypanosoma brucei. Mol Biochem Parasitol.* 2007 Dec;156(2):175-90.
glycosomal membrane protein, putative	Tc00.1047053509203.40	EAN88868		glycosome	Aman RA, Wang CC. Identification of two integral glycosomal membrane proteins in *Trypanosoma brucei. Mol Biochem Parasitol*. 1987 Aug;25(1):83-92.
**SIGNAL TRANSDUCTION**					
neurobeachin/beige protein, putative	Tc00.1047053511159.7	EAN84625	PM	X	N/A. Wang X, Herberg FW, Laue MM, Wullner C, Hu B, Petrasch-Parwez E, Kilimann MW. Neurobeachin: A protein kinase A-anchoring, beige/Chediak-higashi protein homolog implicated in neuronal membrane traffic. *J Neurosci.* 2000 Dec 1;20(23):8551-65.
**OTHERS**					
retrotransposon hot spot (RHS) protein, putative	Tc00.1047053507501.10	EAN84826		X	Bringaud F, Biteau N, Melville SE, Hez S, El-Sayed NM, Leech V, Berriman M, Hall N, Donelson JE, Baltz T. A new, expressed multigene family containing a hot spot for insertion of retroelements is associated with polymorphic subtelomeric regions of *Trypanosoma brucei. Eukaryot Cell.* 2002 Feb;1(1):137-51. Erratum in: *Eukaryot Cell* 2002 Apr;1(2):305.
	Tc00.1047053511773.110	EAN89238	M	X	same as previous
	Tc00.1047053445777.10	EAN80690		X	same as previous
	Tc00.1047053399373.9	EAN82602		X	same as previous
Dispersed gene family protein 1(DGF-1), putative	Tc00.1047053507187.9	EAN82246		X	Lander N, Bernal C, Diez N, Añez N, Docampo R, Ramírez JL. Localization and developmental regulation of a dispersed gene family-1 (DGF-1) protein in *Trypanosoma cruzi. Infect Immun.* 2010 Jan;78(1):231-40
Dispersed gene family protein 1 (DGF-1, pseudogene), putative	Tc00.1047053509961.70			X	Same as previous
