# Supplementary material for: Identification of Contractile Vacuole Proteins in Trypanosoma cruzi
Source: PLoS One. 2011 Mar 18;6(3):e18013. doi: 10.1371/journal.pone.0018013 (PMC3060929; doi:10.1371/journal.pone.0018013)
Supplement: Table S6 — Predicted subcellular locations for proteins in the T. cruzi CV fraction from five targeting prediction servers. This Table includes a list of high confidence (1% false discovery rate, protein group probability >0.95) and low confidence spectral matches curated from 1% false discovery rate dataset guided by CV literature. (PDF) [file pone.0018013.s006.pdf]

Table S6. Predicted subcellular locations for proteins in the *T. cruzi* contractile vacuole fraction from five targeting prediction servers. Included in this list are high confidence (1% false discovery rate, protein group probability >0.95) and low confidence spectral matches curated from 1% false discovery rate dataset guided by contractile vacuole literature. Final predictions are given when there is consensus between two or more prediction servers. *S*, secreted. *C*, cytosol. *M*, mitochondrion. *N*, nucleus. *PM*, plasma membrane. *G*, Golgi complex. *ER*, endoplasmic reticulum, *P*, peroxisome. *L*, lysosome. *CYSK*, cytoskeleton. Threshold probabilities and confidences were set as follows : targetP ($RI_M \leq 3$, $RI_S \leq 4$), pTarget ≥80%, SLP-LOCAL, PA-Sub ≥ 3, WoLfPsort ≥ 80%*. If annotated descriptions of gene products differ from the specific name used in the text, the annotated protein name is given in parenthesis.

Tc#	Name	TargetP Location	TargetP Reliability	pTarget Location	pTarget Confidence	SLP-LOCAL Location	SLP-LOCAL Reliability	PA Sub Location	WoLf PSORT Location	Final Prediction
Tc00.1047053506949.50	cytochrome c, putative			M	100	N C	3	M		M
Tc00.1047053510943.50	delta-1-pyrroline-5-carboxylate dehydrogenase, putative							M		
Tc00.1047053509805.40	Golvesin-1 (methyltransferase, putative)							N		
Tc00.1047053507641.290	chaperonin HSP60, mitochondrial precursor	M	2	M	87.6			M		M
Tc00.1047053506355.60	small G-protein, putative			C	93.9	N C	3	C		C
Tc00.1047053506337.70	2-oxoglutarate dehydrogenase E1 component, putative	M	1	M	100			M		M
Tc00.1047053503899.119	trypanothione/tryparedoxin dependent peroxidase 2, putative			C	87.6			M		
Tc00.1047053509051.20	amastin, putative	S	3						PM	
Tc00.1047053506519.130	inosine-5'-monophosphate dehydrogenase, putative			C	93.9			C		C
Tc00.1047053510533.210	hypothetical protein, conserved (pseudogene)			N	100	N C	3	C		C N
Tc00.1047053511289.70	ADP,ATP carrier protein 1, mitochondrial precursor, putative							M		
Tc00.1047053509551.30	mitochondrial phosphate transporter, putative							M		
Tc00.1047053511773.110	retrotransposon hot spot (RHS) protein, putative	M	2	G	81.4				M	M
Tc00.1047053509317.80	hypothetical protein, conserved			PM	93.9					
Tc00.1047053511151.90	glycerol-3-phosphate dehydrogenase, putative	M	3	M	87.6			M	M	M
Tc00.1047053510395.10	ATP synthase, alpha chain, mitochondrial precursor, putative	M	2	M	100			M	M	M
Tc00.1047053509601.70	vacuolar proton translocating ATPase subunit A, putative								PM	
Tc00.1047053509733.170	transitional endoplasmic reticulum ATPase, putative			C	100	N C	5	C		C
Tc00.1047053506167.50	clathrin heavy chain, putative			C	100			C		C
Tc00.1047053506219.40	hypothetical protein, conserved	S	3	M	100	S	3	ER		S
Tc00.1047053506579.10	ABC transporter, putative					N C	4		PM	
Tc00.1047053511159.7	neurobeachin/beige protein, putative			PM	87.6	N C	6		PM	PM
Tc00.1047053508231.40	hypothetical protein, conserved									
Tc00.1047053508699.130	cation transporter, putative			G	100	N C	4		PM	
Tc00.1047053503815.10	alkyl-dihydroxyacetone phosphate synthase, putative					N C	5	P		
Tc00.1047053509617.20	paraflagellar rod protein 3, putative			C	93.9	N C	5	C		C
Tc00.1047053506491.20	myosin heavy chain, putative			C	93.9	N C	5			C
Tc00.1047053509585.10	dynein heavy chain, putative			C	100			C		C
Tc00.1047053507625.183	SNARE2.1 (hypothetical protein, conserved)			ER	81.4			G		
Tc00.1047053508535.10	NADH-dependent fumarate reductase, putative			M	81.4			M		M
Tc00.1047053503449.30	AP180 (clathrin coat assembly protein, putative)			C	100	N C	4	C		C
Tc00.1047053503455.30	Golvesin-2 (hypothetical protein, conserved)			N	100	N C	5	N		N
Tc00.1047053506563.170	calpain-like cysteine peptidase (pseudogene), putative					N C	4			
Tc00.1047053503571.19	hypothetical protein, conserved	S	1	M	87.6					
Tc00.1047053506025.14	ribosomal protein S29, putative			M	100					
Tc00.1047053411235.9	alpha tubulin, putative			C	81.4				CYSK	
Tc00.1047053508275.9	dynein heavy chain, putative			C	100	N C	3	C		C
Tc00.1047053504949.30	succinate dehydrogenase, putative			G	81.4			M		
Tc00.1047053507093.260	ABC transporter, putative			M	93.9			M	PM	M
Tc00.1047053505843.10	glutamate dehydrogenase, putative			M	100			M	CYSK	M
Tc00.1047053504125.50	mitochondrial carrier protein, putative	S	2					M		
Tc00.1047053506563.40	beta tubulin, putative			C	87.6	N C	3	C		C
Tc00.1047053511909.40	succinate dehydrogenase flavoprotein, putative			M	100			M	M	M
Tc00.1047053508153.130	enoyl-CoA hydratase, mitochondrial precursor, putative			P	81.4			M		
Tc00.1047053506715.50	SNARE2.2 (hypothetical protein)									
Tc00.1047053511211.20	coatomer gamma subunit, putative			G	100	N C	8	G		G
Tc00.1047053507713.30	heat shock protein 85, putative			C	93.9	N C	3	C		C
Tc00.1047053509793.50	hypothetical protein, conserved	M	2	N	81.4			N		N
Tc00.1047053509537.50	hypothetical protein, conserved			G	87.6					
Tc00.1047053508707.310	hypothetical protein, conserved			G	93.9			N		
Tc00.1047053511441.10	calpain cysteine peptidase, putative			N	100	N C	5	C		C N
Tc00.1047053504069.80	ATP synthase F1 subunit gamma protein, putative			C	93.9			M		
Tc00.1047053507927.20	mitochondrial oligo_U binding protein TBRGG1, putative	M	2	L	81.4			N		
Tc00.1047053508723.70	hypothetical protein, conserved	S	2					C		

Tc00.1047053506147.80	hypothetical protein, conserved			P	87.6					
Tc00.1047053510119.20	elongation factor 1-alpha (EF-1-alpha), putative			C	93.9			C	C	C
Tc00.1047053506223.80	ATP-dependent zinc metallopeptidase, putative			M	100	N C	5	M		M
Tc00.1047053509999.90	carnitine/choline acetyltransferase, putative			M	100			M	M	M
Tc00.1047053445777.10	retrotransposon hot spot (RHS) protein, putative			C	81.4	N C	4			C
Tc00.1047053510149.80	ABC transporter, putative	S	2						PM	
Tc00.1047053506779.120	hypothetical protein, conserved			PM	93.9	N C	3			
Tc00.1047053506401.70	vacuolar protein sorting protein 18, putative			C	81.4	N C	3	C		C
Tc00.1047053504427.64	hypothetical protein, conserved			N	87.6	N C	4			N
Tc00.1047053504153.250	hypothetical protein, conserved			PM	93.9				PM	PM
Tc00.1047053505763.19	P-type H+-ATPase, putative					N C	3	PM	PM	PM
Tc00.1047053511029.20	kinetoplast DNA-associated protein, putative	M	2	M	87.6			N		M
Tc00.1047053511531.50	glucosamine-6-phosphate isomerase, putative			C	100	N C	3	C	CYSK	C
Tc00.1047053503449.14	hypothetical protein, conserved	M	1	M	87.6				M	M
Tc00.1047053508153.1100	MP99, putative			G	93.9					
Tc00.1047053503413.4	hypothetical protein, conserved			C	100	N C	5	ER		C
Tc00.1047053506755.250	mitochondrial import inner membrane translocase subunit Tim17, putative			M	100			M		M
Tc00.1047053504069.50	peroxin 14, putative			P	100	N C	3			
Tc00.1047053509045.20	co-chaperone GrpE, putative	M	2	M	100			M	M	M
Tc00.1047053503849.60	NADH-dependent fumarate reductase, putative	S	1	M	100			M		M
Tc00.1047053508241.130	hypothetical protein, conserved	M	3	G	87.6				M	M
Tc00.1047053508153.340	6-phospho-1-fructokinase, putative			P	87.6			C		
Tc00.1047053508741.229	hypothetical protein, conserved					N C	3		S	
Tc00.1047053511751.200	epsilon-adaptin, putative	M	3	M	93.9	N C	3	G		M
Tc00.1047053509611.170	hypothetical protein, conserved	S	1	G	87.6			C		
Tc00.1047053511575.130	folate/pteridine transporter, putative			PM	81.4	N C	4		PM	PM
Tc00.1047053508719.40	kinetoplast DNA-associated protein, putative			M	81.4			N		
Tc00.1047053510837.20	glutamamyl carboxypeptidase, putative							C		
Tc00.1047053511229.30	hypothetical protein, conserved			P	87.6					
Tc00.1047053510089.210	hypothetical protein, conserved			M	93.9				M	M
Tc00.1047053511071.190	hypothetical protein, conserved	S	1	ER	81.4				PM	
Tc00.1047053507777.20	hypothetical protein, conserved			C	100	N C	3			C
Tc00.1047053509463.30	3-ketoacyl-CoA thiolase, putative	M	3	M	93.9			M	M	M
Tc00.1047053506295.70	hypothetical protein, conserved			M	87.6					
Tc00.1047053511217.90	hypothetical protein, conserved	S	1	G	87.6	S	4			S
Tc00.1047053503999.30	hypothetical protein, conserved	M	2			M	3	C		M
Tc00.1047053508719.60	kinetoplast DNA-associated protein, putative			M	81.4			N	M	M
Tc00.1047053509683.10	hypothetical protein	S	1	M	87.6					
Tc00.1047053511277.170	ATP-dependent zinc metallopeptidase, putative			M	100	N C	3	M		M
Tc00.1047053507711.200	hypothetical protein, conserved	S	1	L	93.9					
Tc00.1047053511903.40	hypothetical protein, conserved			M	100			C	M	M
Tc00.1047053508737.210	hypothetical protein, conserved					N C	3		S	
Tc00.1047053508815.179	dynein heavy chain, putative			C	100			C		C
Tc00.1047053506983.39	calpain-like cysteine peptidase, putative			C	93.9	N C	4		C	C
Tc00.1047053508503.20	cytochrome c oxidase subunit V, putative			M	87.6					
Tc00.1047053509215.40	cyclophilin, putative	S	1	ER	100	S	3	C		S
Tc00.1047053509777.130	hypothetical protein, conserved									
Tc00.1047053506933.60	mitochondrial RNA binding protein, putative							N		
Tc00.1047053509701.10	trifunctional enzyme alpha subunit, mitochondrial precursor-like protein, putative			P	87.6	N C	3	M		
Tc00.1047053509561.20	flagellum-adhesion glycoprotein, putative	S	1	L	87.6	S	3			S
Tc00.1047053506657.40	ADP/ATP translocase, putative			PM	81.4	N C	5		PM	PM
Tc00.1047053506025.50	V-H+-ATPase subunit B (vacuolar ATP synthase subunit B, putative)			M	100	N C	4	M		M
Tc00.1047053507029.30	heat shock 70 kDa protein, mitochondrial precursor, putative	M	2	M	81.4			M	M	M
Tc00.1047053504103.20	hypothetical protein, conserved	M	2	M	87.6	M	3		M	M
Tc00.1047053506503.140	extracellular receptor, putative	S	1	PM	100			PM		PM
Tc00.1047053508209.120	10 kDa heat shock protein, putative	M	2	M	100			M	M	M
Tc00.1047053508737.100	ATP-dependent Clp protease subunit, heat shock protein 78, putative	M	1	M	81.4			C	M	M
Tc00.1047053507053.180	hypothetical protein, conserved	S	1							
Tc00.1047053506289.80	Rab32 (small GTP-binding protein, putative)			C	93.9			M		
Tc00.1047053509799.140	hypothetical protein, conserved	S	1			S	4			S
Tc00.1047053503959.10	hypothetical protein, conserved	M	2			M	3	M		M
Tc00.1047053507089.270	dihydrolipoyl dehydrogenase, putative			M	100			M		M
Tc00.1047053509153.120	acyl-CoA dehydrogenase, putative	M	2	M	100	M	4	M	M	M
Tc00.1047053503793.10	2-oxoglutarate dehydrogenase subunit, putative					N C	5	M		

Tc00.1047053506989.190	lipophosphoglycan biosynthetic protein, putative	S	1	M	87.6			ER		
Tc00.1047053510215.10	NADH-dependent fumarate reductase, putative			M	100	N C	3	M		M
Tc00.1047053509961.70	dispersed gene family protein 1 (DGF-1, pseudogene), putative			L	93.9			S	PM	
Tc00.1047053506753.240	hypothetical protein, conserved					N C	5			
Tc00.1047053503843.40	chaperone DnaJ protein, putative			N	93.9	N C	5	C		C N
Tc00.1047053510529.30	hypothetical protein, conserved	S	4	G	87.6					
Tc00.1047053457251.10	3-oxo-5-alpha-steroid 4-dehydrogenase, putative			ER	100	N C	3			
Tc00.1047053506957.110	SNF-7-like protein, putative			N	100	N C	5	C		C N
Tc00.1047053508173.264	hypothetical protein, conserved			P	81.4					
Tc00.1047053506583.60	mitochondrial elongation factor G, putative	M	1	M	93.9	M	3	M	M	M
Tc00.1047053504013.100	ribosomal protein S19, putative			N	81.4					
Tc00.1047053507047.150	hypothetical protein, conserved					N C	3	ER		
Tc00.1047053508045.70	hypothetical protein, conserved			M	87.6					
Tc00.1047053510679.40	hypothetical protein			C	87.6					
Tc00.1047053504147.120	60S ribosomal protein L22, putative			P	87.6					
Tc00.1047053511527.70	myosin heavy chain, putative			C	100	N C	4	C		C
Tc00.1047053506755.20	paraflagellar rod component, putative			C	93.9	N C	3	C		C
Tc00.1047053504087.20	hypothetical protein	S	1	M	81.4				PM	
Tc00.1047053508647.200	triosephosphate isomerase, putative			M	100	N C	3	C		C
Tc00.1047053506445.110	nucleobase transporter, putative			PM	93.9				PM	PM
Tc00.1047053507009.10	Gim5A protein, putative			PM	100					
Tc00.1047053508999.260	calpain-like cysteine peptidase (pseudogene), putative					N C	4			
Tc00.1047053511389.150	thiolase protein-like protein, putative	M	2	M	100	M	3	M	M	M
Tc00.1047053507739.110	myosin IB heavy chain, putative			C	87.6			C		C
Tc00.1047053510101.140	pyruvate phosphate dikinase, putative			C	100	N C	5			C
Tc00.1047053504163.60	hypothetical protein, conserved			N	100	N C	3			N
Tc00.1047053509109.30	hypothetical protein, conserved			C	100					
Tc00.1047053510003.20	hypothetical protein, conserved	M	2						M	M
Tc00.1047053509797.40	isoleucyl-tRNA synthetase, putative			M	87.6			C		
Tc00.1047053507501.10	retrotransposon hot spot (RHS) protein, putative			M	93.9					
Tc00.1047053510773.20	vacuolar-type proton translocating pyrophosphatase 1, putative	S	1	M	81.4			M	PM	M
Tc00.1047053508981.39	trifunctional enzyme alpha subunit, mitochondrial precursor-like protein			M	100	M	3	M	M	M
Tc00.1047053503893.30	hypothetical protein, conserved			G	81.4	N C	3			
Tc00.1047053504035.84	hypothetical protein, conserved	M	2							
Tc00.1047053506445.60	mitochondrial DNA topoisomerase II, putative			N	100	N C	3	N		N
Tc00.1047053507547.90	glycosomal phosphoenolpyruvate carboxykinase, putative			G	81.4			C		
Tc00.1047053509203.40	glycosomal membrane protein, putative			P	81.4	N C	3			
Tc00.1047053509679.9	leucyl-tRNA synthetase, putative			ER	81.4			C		
Tc00.1047053510155.70	heat shock protein 70 (HSP70), putative			C	100			ER		
Tc00.1047053504037.30	60S ribosomal protein L12, putative			M	100	N C	3	C		C
Tc00.1047053504105.130	calcium channel protein, putative					N C	3	PM	PM	PM
Tc00.1047053507187.9	dispersed gene family protein 1 (DGF-1), putative			L	93.9				PM	
Tc00.1047053506249.70	ABC transporter, putative					N C	4		PM	
Tc00.1047053506963.14	40S ribosomal protein S27, putative					N C	3		S	
Tc00.1047053509053.70	p22 protein precursor, putative	M	1	M	100			M	M	M
Tc00.1047053508177.10	hypothetical protein, conserved					N C	3	C		C
Tc00.1047053509445.39	glutamate dehydrogenase, putative	M	1					M		M
Tc00.1047053510855.10	peptide methionine sulfoxide reductase, putative			G	87.6				C	
Tc00.1047053509911.74	hypothetical protein, conserved	M	2						M	M
Tc00.1047053509233.180	ATPase beta subunit, putative	M	2	M	100	M	4	M	M	M
Tc00.1047053506585.40	glucose-regulated protein 78, putative	S	1			S	4	ER		S
Tc00.1047053506551.10	hypothetical protein, conserved			PM	81.4				PM	PM
Tc00.1047053507715.34	hypothetical protein, conserved			G	81.4					
Tc00.1047053455721.9	cytochrome c oxidase subunit 10, putative			M	87.6					
Tc00.1047053506563.79	calpain-like cysteine peptidase (pseudogene), putative			C	93.9	N C	4			C
Tc00.1047053507689.30	glutamamyl carboxypeptidase, putative							C		
Tc00.1047053506577.120	sterol C-24 reductase, putative									
Tc00.1047053511003.190	hypothetical protein, conserved			P	81.4	N C	4			
Tc00.1047053508827.40	acyl-CoA dehydrogenase, putative	M	2	M	100			M	M	M
Tc00.1047053508413.68	kinetoplastid membrane protein KMP-11			C	87.6	N C	4	C		C
Tc00.1047053504147.70	hypothetical protein, conserved	S	4	M	81.4					
Tc00.1047053399373.9	retrotransposon hot spot (RHS) protein, putative	M	1	G	87.6					
Tc00.1047053506355.10	hexose transporter, putative			PM	100	N C	4	PM	PM	PM
Tc00.1047053504153.160	carboxypeptidase, putative			G	100			S		

Tc00.1047053511277.290	aconitase, putative	M	3	M	87.6			C	M	M
Tc00.1047053511733.90	hypothetical protein, conserved									
Tc00.1047053507711.60	hypothetical protein, conserved			C	100	N C	8	C		C
Tc00.1047053511529.160	enoyl-CoA hydratase/isomerase family protein, putative			M	87.6			M		M
Tc00.1047053433273.10	dynein heavy chain, putative			G	93.9	N C	3	C		C
Tc00.1047053509499.14	tryparedoxin peroxidase, putative	M	2	M	81.4	M	3	M	M	M
Tc00.1047053503903.60	hypothetical protein, conserved			S	87.6					
Tc00.1047053508719.30	hypothetical protein, conserved	M	2	N	81.4			N		N
Tc00.1047053506211.160	ADP,ATP carrier protein 1, mitochondrial precursor, putative							M		
Tc00.1047053408437.20	hypothetical protein, conserved			PM	87.6					
Tc00.1047053506755.260	mitochondrial RNA-binding protein 2, putative	M	2	G	81.4				M	M
Tc00.1047053506839.70	NADH dehydrogenase, putative							M	M	M
Tc00.1047053506977.60	hypothetical protein, conserved									
Tc00.1047053504153.310	heat shock protein, putative	M	3	M	93.9			M	M	M
Tc00.1047053508999.250	calpain-like cysteine peptidase (pseudogene), putative									
Tc00.1047053509237.130	cytoskeleton-associated protein CAP5.5, putative			C	81.4	N C	3	C		C
Tc00.1047053506297.190	pyruvate phosphate dikinase, putative			C	100	N C	5			C
Tc00.1047053504089.50	flagellar radial spoke component, putative			C	93.9	N C	4			C
Tc00.1047053508547.160	hypothetical protein, conserved			N	87.6	N C	4	C	M	C N
Tc00.1047053506247.220	histidine ammonia-lyase, putative			P	81.4					
Tc00.1047053503837.10	hypothetical protein, conserved			ER	87.6				PM	
Tc00.1047053506839.30	hypothetical protein, conserved			C	93.9			C	CYSK	C
Tc00.1047053508555.60	cytoskeleton-associated protein CAP5.5, putative			N	81.4			C		
Tc00.1047053510353.30	paraflagellar rod component Par4, putative			N	93.9	N C	6	G C		N
Tc00.1047053504427.60	hypothetical protein, conserved	S	1							
Tc00.1047053503769.40	cytochrome c oxidase VII, putative	M	3	M	100					M
Tc00.1047053507711.300	hypothetical protein, conserved					N C	4			
Tc00.1047053503887.40	hypothetical protein, conserved	M	2	M	87.6	M	3			M
Tc00.1047053511215.119	69 kDa paraflagellar rod protein, putative			C	100	N C	5	C		C
Tc00.1047053509351.10	delta-1-pyrroline-5-carboxylate dehydrogenase, putative					N C	3	M		
Tc00.1047053505945.20	ribonuclease mar1, putative			C	81.4					
Tc00.1047053510797.30	hypothetical protein, conserved			C	93.9	N C	3	C		C
Tc00.1047053511269.60	vacuolar protein sorting complex subunit, putative	M	3	C	100			C		C
Tc00.1047053506163.50	hypothetical protein, conserved			M	100					
Tc00.1047053508153.270	heat shock protein 20, putative							C		
Tc00.1047053407477.50	cytochrome c oxidase VIII (COX VIII), putative	M	1	M	87.6				M	M
Tc00.1047053505169.10	null			N	100	N C	7		PM	
Tc00.1047053506195.110	malate dehydrogenase, putative							M		
Tc00.1047053506201.170	hypothetical protein, conserved			N	93.9	N C	4	C		
Tc00.1047053506275.20	hslvu complex proteolytic subunit-like, putative	M	3	M	87.6			C	M	M
Tc00.1047053506475.116	glutaredoxin, putative			P	81.4			C		
Tc00.1047053506587.70	hypothetical protein, conserved	M	2	M	87.6	M	3			M
Tc00.1047053506629.40	hypothetical protein, conserved	M	2					S	M	M
Tc00.1047053506679.100	40S ribosomal protein S18, putative			M	81.4			C		
Tc00.1047053506789.140	hypothetical protein, conserved			C	93.9	N C	6	C		C
Tc00.1047053506789.240	hypothetical protein, conserved	M	2	M	100					M
Tc00.1047053506893.100	hypothetical protein, conserved			C	81.4	N C	3			
Tc00.1047053506943.50	glyceraldehyde 3-phosphate dehydrogenase, putative			C	81.4			C		C
Tc00.1047053507941.150	histone H4, putative			C	87.6	N C	3	N		
Tc00.1047053508209.100	10 kDa heat shock protein, putative	M	2	M	81.4			M	M	M
Tc00.1047053508741.170	hypothetical protein, conserved			C	81.4					
Tc00.1047053509129.10	hypothetical protein, conserved					N C	3			
Tc00.1047053509965.394	amastin, putative	S	3	PM	81.4					
Tc00.1047053511071.130	basal body component, putative			N	100	N C	6	C		
Tc00.1047053511211.160	heat shock protein 70 (HSP70), putative			C	93.9	N C	3			
Tc00.1047053511635.10	histone H2B, putative			N	93.9			N	N	N
Tc00.1047053511809.130	40S ribosomal protein S15, putative			C	87.6			C	C	C

***80% confidence threshholds for WoLFPSORT (animal) predictions were estimated visually using supplemental graphs (Horton et al, 2007; http://wolfpsort.org/empiricalConfidenceByNumNeighbors/animal/statistics_animal.html, 10/24/2008) of % prediction accuracy as a function of number of nearest neighbors. All cytoskeleton (CYSK) and Golgi (G) predictions are provided as no accuracy estimates were made. Confidence of lysosome predictions for WoLFPSORT have a maximal accuracy of ~55% (10 nearest neighbors). All lysosome predictions with ≥ 10 nearest neighbors are reported.**
