# Supplementary material for: Identification of Contractile Vacuole Proteins in Trypanosoma cruzi
Source: PLoS One. 2011 Mar 18;6(3):e18013. doi: 10.1371/journal.pone.0018013 (PMC3060929; doi:10.1371/journal.pone.0018013)
Supplement: Table S7 — Common features of CV proteins identified by the ELM server. This Table list common features of proteins identified in the CV fraction. (PDF) [file pone.0018013.s007.pdf]

Table S7. Common features of CV proteins identified by the ELM server

**Protein**	**CK1 phosphorylation site (MOD_CK1 1)**	**CK2 phosphorylation site (MOD_CK2 1)**	**GSK3 phosphorylation recognition site (MOD_CSK3 1)**	**Tyrosine-based sorting signal responsible for the interaction with μ subunit of adaptor protein complex (TRG ENDOCYTIC 2)**	**Generic motif for N-glycosylation (MOD N-GLC 1).**	**Substrate recognition site that interacts with cyclin and thereby increases phsophorylation by cyclin/cdk complexes. Predicted protein should have the MOD–CDK site. Also used by cyclin inhibitors (LIG CYCLIN 1)**
AP180	SPQSEEN (406-412)<br>SSATDAI (469-475)	QPRSGKE (348-354)<br>AAFSMDE (368-374)<br>(25-31)*<br>(145-151)*	TCGSEIKT (321-328)<br>EKASPPLS (335-342)<br>QPRSGKES (348-355)<br>APVSWEGT(437-444)<br>GTPSVQYS (443-450)<br>VQYSTAAT (447-454)<br>(165-172)*<br>(170-177)*<br>(269-276)*	YAAL (422-425)<br>(216-219)*<br>(227-230)*<br>(259-262)*	(117-122)*	KELY (485-488)
V-H⁺-ATPase, B	SGYSVKP (27-33)	RMLSKQE (10-16)<br>FFRTEFE (228-234)<br>LALTTAE (262-268)<br>(54-60)*<br>(97-103)*<br>(481-487)*	MIQTGISS (159-166)<br>ENGSMEKT (235-242)<br>VILTDMSS (280-287)<br>PDLTGYIT (351-358)	YRDI (140-143)<br>YTDL (313-316)<br>YPPI (374-377)<br>(37-40)*	FNGSGI (122-127)<br>ENGSME (235-240)	RQLYP (371-375)
Rab11	(33-39)*<br>(94-100)*<br>97-103)*	(25-31)*<br>(142-148)*<br>(147-153)*<br>(152-158)*	(18-25)*<br>(87-94)*<br>(90-97)*<br>(142-149)*	(169-172)*	(2-9)*<br>(90-95)*	-
Rab32	(4-10)*<br>(119-125)*	GTETAQE (210-216)<br>GNRTRRE (230-236)<br>(92-98)*<br>(140-146)*	(35-42)*<br>(92-99)*<br>115-122)*	(74-77)*	KNTSGK (221-226)*<br>GNRTRR (230-235)*	(12-16)*

		(160-166)*<br>(180-186)*				
Vamp1	SFKSQFA (84-90)<br>SEITPVV (100-106)<br>STLTRTF (115-121)	LTRTFNE (117-123)	YPRSSEIT (96-103)<br>TLSTLTRT (113-120)	YHFL (51-54)<br>YCCV (61-64)	QNRTVF (70-75)	KMLL (185-188)
Pho1	STESGKD (501-507)<br>SLITIIL (520-526)<br>(32-38)*<br>(105-111)*<br>(114-120)*	DIDSDYE (223-229)<br>LARTNLE (355-361)<br>IQRTLWE (407-413)<br>(32-38)*<br>(65-71)*	INRSRLVT (205-212)<br>LIVSLWIT (286-293)<br>VANSTVAS (323-330)<br>GMLSPIAS (436-443)<br>STESGKDT (501-508)<br>LAHSMHGT (590-597)<br>ALATTFVS (615-622)<br>TFSHTVS (619-626)<br>(98-105)*<br>(110-117)*<br>(111-118)*<br>(129-136)*	YKNV (239-242)<br>YLTF (604-607)<br>YVVL (720-723)<br>(22-25)*	INRSRL (205-210)<br>ANSTVA (324-329)	RSLM (218-221)<br>KMLDI (585-589)<br>(26-30)*<br>(86-90)*<br>(135-139)*
AQP1	-	-	MTFSPGMS (1-8)<br>FLASLYGS (89-96)<br>VNYSRQRS (136-143)<br>LGGSFLAS (202-209)	YQIL (212-215)	VNYSRQ (136-141)	KPLIF (189-193)
VP1	SLTSVFA (192-198)<br>SEFTYMM (372-378)<br>STMSIAL (540-546)<br>SAFTMKS (639-645)	AYSSPQE (163-169)<br>LFGSFGE (349-355)	GGNTKERT (59-66)<br>PLMSADVT (68-75)<br>YVISRRVS (124-131)<br>AVYTNART (206-213)<br>GLFSLFVT (252-259)<br>AAGSAEL (364-371)<br>AELSSEFT (368-375)<br>LFSTVAAT (419-426)<br>DTFTVGTT (439-446)<br>TVGTTATT (442-449)<br>GYTTEYYT (48-475)<br>ACETGAAT (488-495)<br>MAVTIFAS (512-522)<br>STMSIALT (540-547)<br>VAISASNT (7272-734)	YRYM (142-145)<br>YMGL (144-147)<br>YECV (278-281)<br>YPLL (379-382)<br>YHPV (479-482)<br>YFSV (505-508)<br>YGPI (551-554)	ANNSGV (399-404)<br>GNTTAA (582-587)	KLLL (32-35)<br>RQLLF (416-420)<br>RALV (452-455)

POT1	SLPTGLA (97-103)<br>SLTSAYV (150-156)<br>SFLSMVT (234-240)<br>SMVTWNY (237-243)<br>SIVSGVG (323-329)<br>SMVSLAI (453-459)<br>SSFSAQR (576-582)	LNCSGIE (181-187)	GGESNFQT (4-11)<br>FHPTLRRT (23-30)<br>ELSTAVPS (106-113)<br>IITTVFIT (134-141)<br>SLTSAYVT (150-157)<br>AYVTGIVS (154-161)<br>TLTSMCCT (331-338)<br>AIVSSMVS (449-456)<br>GNGSGTAT (544-551<br>TSPTGMMS (569-576)	YVSF (128-131)<br>YPSL (148-151)<br>YANI (243-246)<br>YYLF (317-320)<br>YASL (408-411)	GNATYP (144-149)<br>LNCSGI (181-186)<br>VNASSI (225-230)<br>LNATVT (375-380)<br>GNGSGT (544-549)	RMLLI (403-407)
SNARE2.1	SAVSFLT (71-77)<br>SFLTMCD (74-80)<br>SSRSMNV (135-141)<br>SSMSSRG (167-173<br>SMSSRGG (168-174)	TDTSSAE (20-26)<br>QYGSQVE (102-108)	SFLTMCDT (74-81)<br>VFATANSS (129-136)<br>SSRSMNVS (135-142)<br>GIDSKTGS (160-167)<br>KTGSSMSS (164-171)<br>(17-24)*	YCFI (114-117)<br>(205-208)*	ANSSRS (133-138)<br>MNVSRV (139-144)	KTLL (35-38)
SNARE2.2	SRATALK (75-81)	DIRSDVE (121-127)<br>LQSSALE (152-158)	-	YALV (6-9)<br>YDML (55-58)<br>YMCV (66-69)<br>YVFI (183-186)	-	RPLV (40-43)<br>KFLEV (81-85)<br>RILIY (179-183)
CaM	-	GTITTKE (26-32)<br>QNPTEAE (42-48)<br>MQDSDSE (77-83)<br>DSDSEEE (79-85)<br>EKLTDEE (115-121)<br>(99-105)*	(32-39)*	(139-142)*	-	-
PDEC	SGETSRV (109-115)<br>SRVSPPN (113-119)<br>SSETVVK (236-242)<br>SSKTVEP (718-724)<br>SRETELI (803-809)<br>SEESASM (810-816)<br>SATTLLT (916-922)<br>(20-26)*<br>(29-35)*<br>(59-65)*	EGSSFRE (82-88)<br>LLQSREE (258-264)<br>FFASQEE (341-347)<br>ETPSSVE (561-567)<br>LFPSIEE (588-594)<br>ELRTASE (652-658)<br>RRGSKAE (734-740)<br>RFSTQWE (821-827)<br>LSATLAE (866-872)	RVESGETS (106-113)<br>SGETSRVS (109-116)<br>RSGTPQVS (195-202)<br>EVASRVPS (319-326)<br>RVPSVLQS (323-330)<br>TPSSVEKS (562-569)<br>TGDSRPRS (726-733)<br>RPRSRRGS (730-737)<br>RRGSKAET (734-741)<br>EFKTPCFS (796-803)	YREL (836-839)	(450-455)*<br>(462-467)*	RRLPP (281-285)<br>RGLPV (552-556)<br>RELY (603-606)<br>RQLL (648-651)<br>KRLKP (789-793)<br>RGLHP (895-899)

	(382-388)*<br>(483-489)*	(392-398)*<br>(442-448)*<br>(462-468)*<br>(501-507)*<br>(621-627)*	SRETELIS (803-810)<br>ESASMDVT (812-819)<br>MDVTHRFS (816-823)<br>MSATTLLT (915-922)<br>(67-74)*<br>(426-433)*<br>(430-467)*			

*Excluded ELMs falling inside SMART/PFAM domains and/or scoring poorly with the structural filter (if applicable). Matches in this list are only likely to be of interest if they are accessible in surface-exposed loops.
