# Supplementary material for: Identification of Contractile Vacuole Proteins in Trypanosoma cruzi
Source: PLoS One. 2011 Mar 18;6(3):e18013. doi: 10.1371/journal.pone.0018013 (PMC3060929; doi:10.1371/journal.pone.0018013)
Supplement: Table S8 — Primers used to generate expression constructs of contractile vacuole proteins. This Table includes all primers used in this work. (PDF) [file pone.0018013.s008.pdf]

Table S8. Primers used to generate expression constructs of contractile vacuole proteins.

Name	Gene ID	GFP	Expression vector	Forward (5'-3')	Reverse (5'-3')
V-H^{+}-ATPase Subunit B	Tc00.1047053506025.50	C-terminal	pTEX	GGATCCATGGGCATACATGAGGCAGAGGAG	AAGCTTCTTCCGCTCGGGTTGGCGGTCGTAG
SNARE 2.1	Tc00.1047053507625.183	C-terminal	pTEX	GGATCCATGCTTTTTTTTACTCTTATCGTC	AAGCTTTGCCAAAGCGGCATAGTAAAATATG
SNARE 2.2	Tc00.1047053506715.50	C-terminal	pTEX	GGATCCATGGTGACGATTCGTTACGCCCTTG	AAGCTTATTTCTTTTGCAGCGATTAAAGTTG
AP180	Tc00.1047053503449.30	N-terminal	pTEX	GGATCCATGAATGTGAAAGATTCTAATGAAC	AAGCTTAATGTTATTGGCATGCCAACCCTT
Rab11	Tc00.1047053511407.60	N-terminal	pTEX	GGATCCATGGAAAACACAAATTTGAC	AAGCTTCTAGCAGCACCTACCAGTAT
Rab32	Tc00.1047053506289.80	N-terminal	pTEX	GGATCCATGTCATACTCGAAGAGCAGTG	AAGCTTTTAACAGGAGCAGCCCGACTTTTC
TcPho1	Tc00.1047053508831.60	C-terminal	pTREX	TCTAGAATGAAGTTTGGAAAACGGTTTC	AAGCTTGTTGAAACCTTCAAGCACAAC
CaM	Tc00.1047053507483.60	C-terminal	pTREX	TCTAGAATGGCTGATCAACTGTCCAA	AAGCTTCTTGCTCATCATCATCTTGAC
VAMP1	Tc00.1047053511627.60	N-terminal	pTEX	GGATCCATGGCCATTATATCATCTTTTGTT	AAGCTTCTATTTTTTGCACTTTTTAAAATCC
