# Supplementary figures and images for: Identification of Contractile Vacuole Proteins in Trypanosoma cruzi
Source: PLoS One. 2011 Mar 18;6(3):e18013. doi: 10.1371/journal.pone.0018013 (PMC3060929; doi:10.1371/journal.pone.0018013)

**A**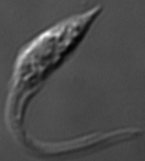**B****AP-180**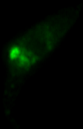**C****CaM**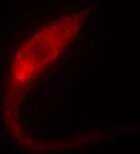**D****Merge**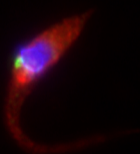

Supplement: Figure S1 — Immunofluorescence microscopy of AP180. A. DIC. B. AP180-GFP. C. Calmodulin. D. Merge. AP180-GFP is shown in green, calmodulin in red, and DAPI in blue. Scale bars = 5 µm. (PDF) [file pone.0018013.s009.pdf]

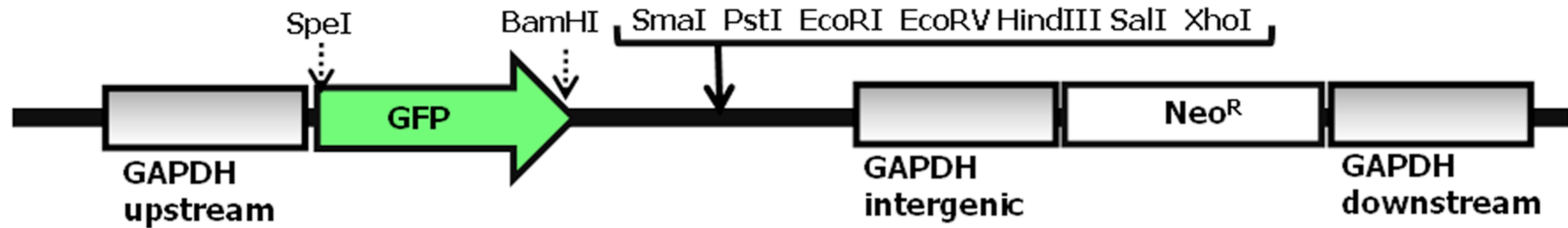

Figure S2

Supplement: Figure S2 — Map of vector GFP-pTEX, an N-terminal GFP fusion vector for T. cruzi. This figure shows that the GFP gene was inserted between SpeI and BamHI. GFP is in frame with both SpeI and BamHI. (PDF) [file pone.0018013.s010.pdf]
